# Dynamic Covalent Functionalization of DNA Origami via Iminoboronate Chemistry

**DOI:** 10.1021/jacs.6c08320

**Published:** 2026-08-05

**Authors:** Alejandro Postigo, Enrique Guerreiro, Joseba Ruiz, M. Sofía Navarrete-Burgos, William Verstraeten, Kerstin Göpfrich, Silvia Hernández-Ainsa, Jesús del Barrio

**Affiliations:** ∇ 82976 Instituto de Nanociencia y Materiales de Aragón (INMA), CSIC-Universidad de Zaragoza, 50009 Zaragoza, Spain; ‡ Certest Biotec S.L., San Mateo de Gállego, 50840 Zaragoza, Spain; § 9144 Heidelberg University, Center for Molecular Biology of Heidelberg University (ZMBH), Berliner Str. 53, 69120 Heidelberg, Germany; ∥ Cluster of Excellence SynthImmune, Heidelberg University, 69117 Heidelberg, Germany

## Abstract

Reversible functionalization of DNA nanostructures is essential for their integration into dynamic biological constructs and biomedical applications. Current strategies typically rely on strand displacement or stimuli-responsive sequences, while approaches based on dynamic covalent chemistry remain largely unexplored. Yet, dynamic covalent bonds combine covalent stability with reversible exchange under mild conditions, offering adaptable and biologically compatible routes to modulate DNA nanostructures. Here, we report iminoboronate conjugation as a strategy for the reversible functionalization of DNA origami. Polyethylene glycol and cholesterol moieties bearing 2-formylphenylboronic acid are efficiently conjugated to DNA origami displaying α-effect amines, while conjugate displacement can be triggered by a small-molecule competitor. We further demonstrate the reversible anchoring of DNA origami to lipid bilayer systems, including both large and giant unilamellar vesicles. These findings establish iminoboronate chemistry as a robust, stimuli-responsive platform for interfacing DNA origami with functional moieties, paving the way for programmable nanodevices and adaptive biomimetic materials.

## Graphical abstract

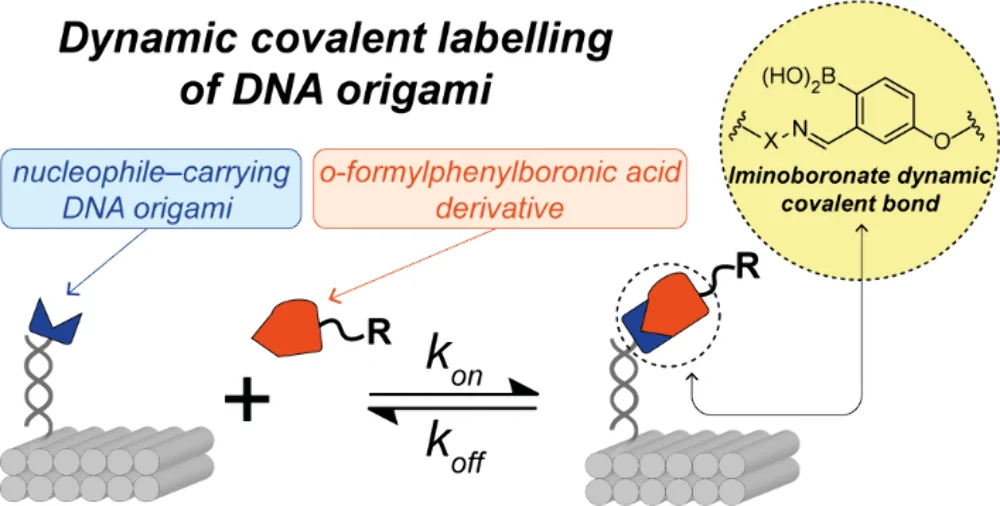

Precise and controllable functionalization is essential for expanding the applicability of DNA nanostructures. Such constructs are typically modified with molecular species (e.g., dyes, drugs, or bioactive molecules) through oligonucleotides bearing chemical handles – including amines, thiols, or azides– that hybridize to complementary DNA overhangs incorporated into the nanostructure design.^1^ However, these modifications generally rely on conventional covalent bond formation strategies that yield irreversible linkages and may present limited applicability in certain contexts. In areas such as targeted drug delivery or synthetic biology, the ability to reversibly attach and release molecules through dynamic chemistries is therefore highly desirable, enabling reconfigurable and responsive systems.

Dynamic covalent chemistry (DCC) offers a promising alternative, as certain reactions enable efficient yet reversible covalent bond formation under mild aqueous conditions.^2^ Several DCC reactions involving, for example, thiols, disulfides, and hydrazones, have been explored to introduce dynamic functionalities into DNA systems.^3–7^ However, these approaches have largely been limited to the modification of individual DNA strands, and some suffer from slow reaction kinetics as well as limited modularity and exchangeability.^8–10^ Consequently, the reversible conjugation of distinct molecular species to DNA nanostructures through DCC remains a significant challenge, yet one that holds considerable promise for enabling new functional and adaptive DNA-based materials.

One particularly attractive strategy for forming robust yet reversible bonds in aqueous media relies on the condensation of amines – particularly α-effect amines such as hydrazides and hydroxylamines – with 2-formyl and 2-acetylphenylboronic acids.^11,12^ Notably, 2-formylphenylboronic acid (FPBA) has emerged as a prototypical scaffold for rapid and reversible bond formation under aqueous conditions. The resulting adducts, also known as iminoboronates, form substantially more rapidly than conventional hydrazones and oximes, owing to the neighboring-group effect of the boronic acid moiety, which stabilizes the imine-type linkage. Moreover, these condensation products are highly stable and can form quantitatively at micromolar to millimolar concentrations, while still retaining the capacity for dynamic exchange under appropriate conditions.^13^ To date, such adducts have been explored primarily in the context of protein, peptide, and antibody modification, and only more recently for irreversible DNA tagging in the development of DNA-encoded libraries.^14–18^ In contrast, their application to the reversible functionalization of DNA nanostructures remains unexplored.

In this work, we harness dynamic iminoboronate chemistry to enable modular and reversible functionalization of DNA origami nanostructures (Figure 1a and S1) using derivatives of **1** (Figure 1b), including hydrophilic polymer **2** and hydrophobic tags such as compound **3** and cholesterol-containing derivative **4**. This approach allows tunable modification of complex DNA architectures and their higher-order assemblies (*vide infra*), establishing a versatile platform for the adaptive functionalization of DNA-based nanomaterials.

**Figure 1:**
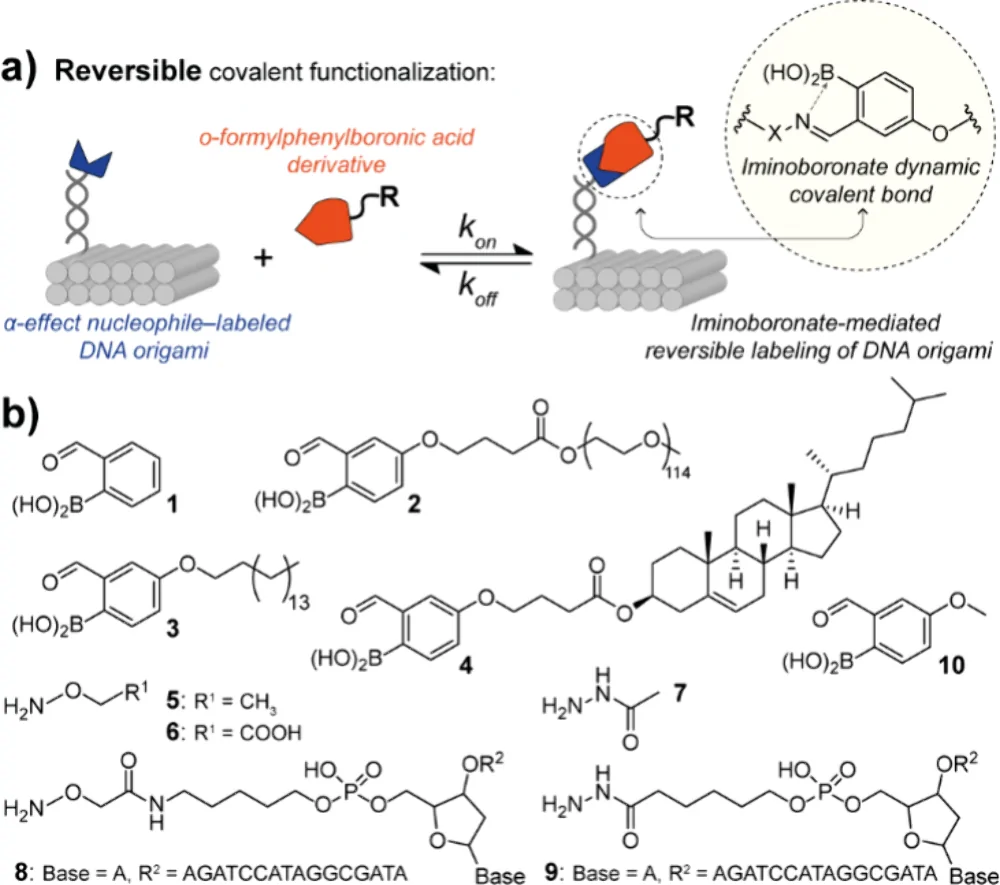
(a) Schematic representation of the approach used to incorporate dynamic modifications into DNA origami through iminoboronate chemistry. (b) Structures of compounds **1–7** and **10** and oligonucleotides **8** and **9**.

As an initial assessment, we performed a series of isothermal titration calorimetry (ITC) experiments to evaluate the formation of iminoboronate adducts with ssDNA bearing α-effect amines at their 5′ terminus (**8** and **9**, Figure 1b). These experiments were conducted following our recently established methodology,^19^ and were benchmarked against model reactions between FPBA and low-molecular-weight α-effect amines (**5** and **7**, Figure 1b). The hydrazone formed from the reaction between **1** and **9** exhibited thermodynamic and kinetic parameters (*K*_d_ ≈ 2.4 × 10^–5^ M; *k*_on_ ≈ 0.60 × 10^3^ M^–1^ s^–1^) (Figure 2a) comparable to those observed for the corresponding low-molecular-weight model reaction between **1** and **7** (Figure 2b and Table S2). These results demonstrate that iminoboronate bond formation can be efficiently carried out on ssDNA while retaining the kinetic and thermodynamic characteristics observed in small-molecule systems. Analogous experiments were performed with **8** (Figure 1b), the ssDNA analogue of **9**, bearing a terminal hydroxylamine group instead of a hydrazide. While the kinetics of bond formation remained similarly rapid, the resulting oxime adduct exhibited more than an order-of-magnitude greater thermodynamic stability than the corresponding hydrazone (Figure S2).

**Figure 2:**
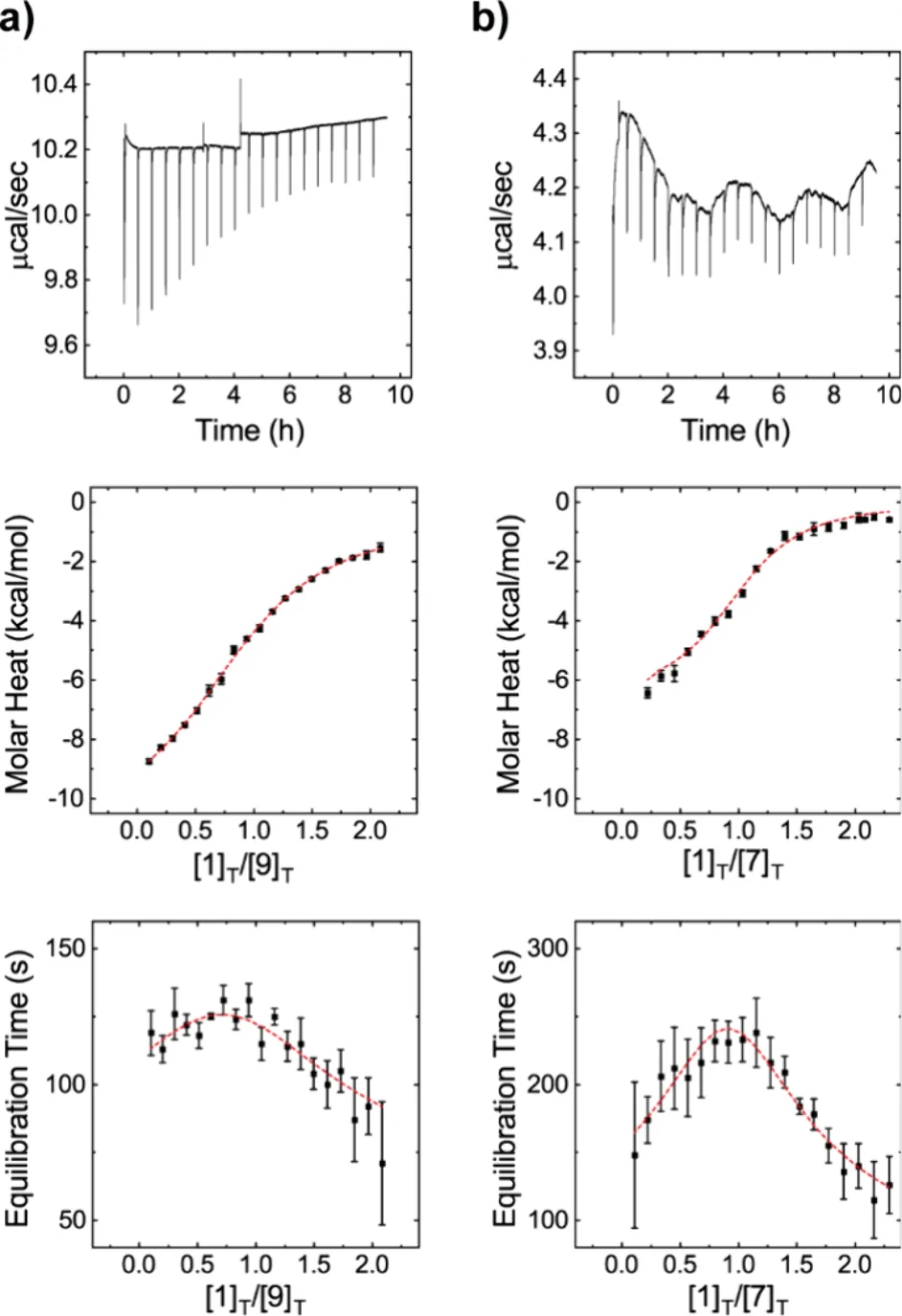
Representative ITC thermograms (top), integrated binding isotherms (center), and equilibration time curves (bottom) for the reversible covalent conjugation between **1** and compounds **9** (a) and **7** (b).

We next evaluated iminoboronate formation between the ssDNA and the PEG derivative **2** (Figure 1b), monitoring the reaction by polyacrylamide gel electrophoresis (PAGE). Distinct retarded bands corresponding to the ssDNA–PEG conjugate, consistent with the increased molecular weight of the conjugated product relative to the individual reactants, were observed (Figure S3a). Notably, higher conversion was obtained for **8** compared to **9** (Figure S3b), in agreement with our ITC measurements showing greater stability of the oxime adduct relative to the hydrazone analogue. Remarkably, PAGE analysis showed that the PEG conjugates formed from both **8** and **9** remained stable for at least 1 week at room temperature, r.t. (Figure S4). Furthermore, conjugation proceeded efficiently under diverse conditions (Figure S5), including cell culture media, demonstrating its compatibility with biologically relevant conditions– a key requirement for potential biological applications.^20^

The reversibility of the conjugation was next evaluated by incubating PEG-functionalized ssDNA derived from **8** and **9** with *O*-(carboxymethyl)­hydroxylamine (**6**, Figure 1b). Preformed DNA–PEG conjugates (prepared from equimolar amounts of the individual components) were treated with **6** (1.2 equiv) and incubated for 1 h at r.t. Dynamic exchange was readily observed for the conjugate derived from **9**, whereas the analogue derived from **8** remained largely unchanged (Figure S6a,b). Reversion of the latter required a 10-fold excess of **6** and extended incubation for 24 h (Figures S6c and S7), consistent with the higher intrinsic stability of oxime linkages relative to hydrazones indicated by ITC measurements.

Having established efficient and reversible conjugation of PEG derivative **2** to ssDNA, we next applied this strategy to a more complex DNA nanostructure: a two-layer square DNA origami (∼55 × 55 × 4 nm^3^).^21^ The origami (ORI) (Figure 3a and S1a) was designed to present 96 ssDNA overhangs (48 per face), each hybridized to strands bearing hydroxylamine or hydrazide (**8** and **9**, Figure 1b). ORI was incubated with **2** in 0.5× TE buffer containing 6 mM MgCl_2_ for 24 h at r.t. Agarose gel electrophoresis (AGE) analysis revealed mobility retardation for ORI displaying **8**, when treated with an excess of **2** relative to the hydroxylamine groups, indicating successful conjugation (Figure 3b and Figure S8a). This result was further supported by the increase in hydrodynamic diameter (Dh), as measured by DLS (Figure 3c) and by AFM (Figure S9). In contrast, ORI displaying **9** showed no significant mobility shift under identical conditions (Figure S8b). The reduced conjugation efficiency likely reflects the higher lability of the hydrazone linkage relative to the corresponding oxime as observed by ITC (see Table S2). This effect is further amplified in the ORI-based assays, where the concentration of α-effect amine functionalities is approximately 10-fold lower than in the ssDNA assays (∼1 μM in ORI vs ∼10 μM in ssDNA assays). Additionally, standard buffers for DNA origami preparation – including the one used in this study– contain Tris,^22^ which is known to interact with 2-formylphenylboronic acid derivatives under neutral aqueous conditions.^23^ In agreement with this, PAGE analysis confirmed a decrease in coupling efficiency between **2** and the α-effect amine–terminated oligonucleotides **8** and **9** at concentrations mimicking the ORI assays (∼1 μM) when performed in 1× TE instead of PBS (Figure S10).

**Figure 3:**
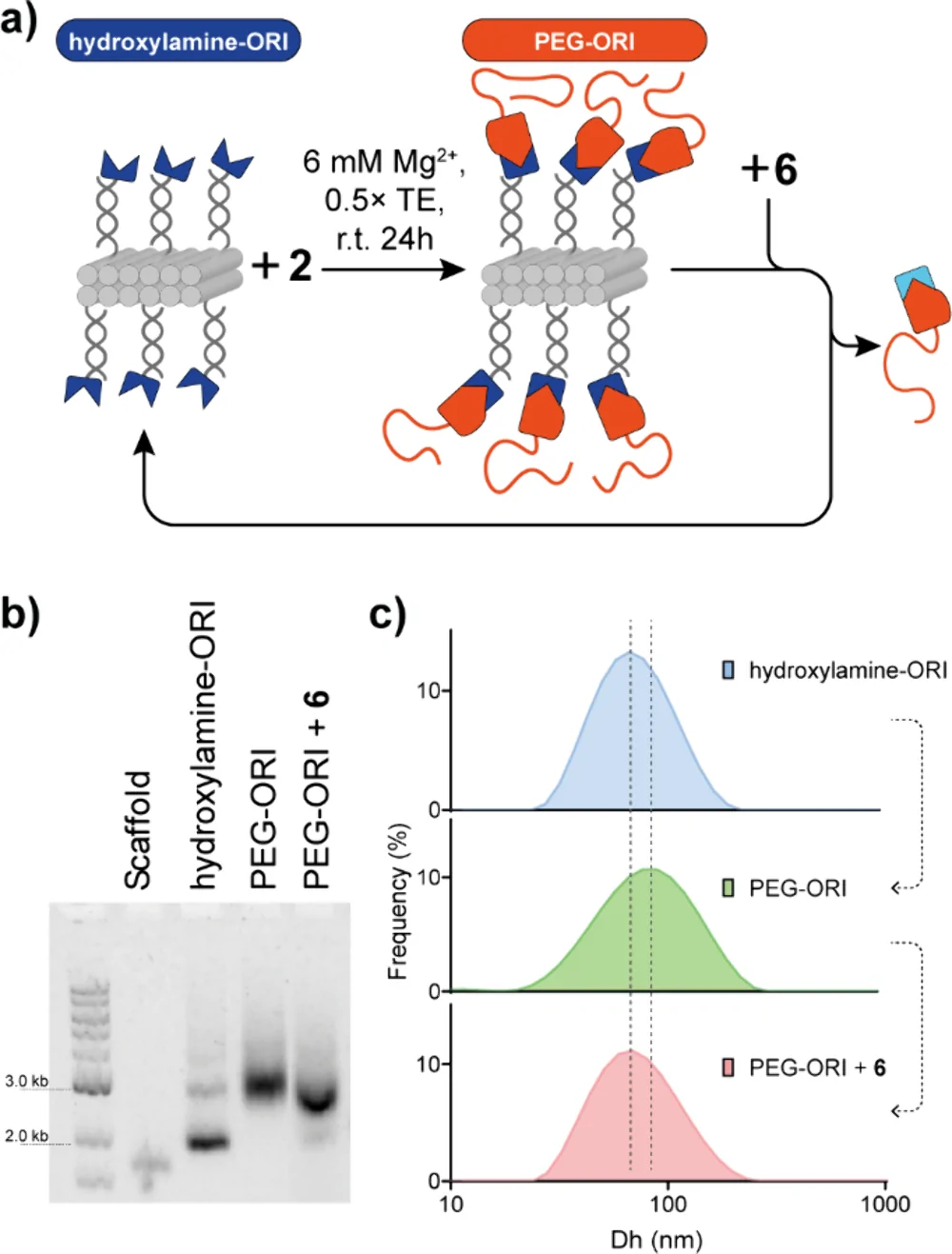
(a) Schematic of the functionalization of ORI with **2** and reversion with **6**. (b) AGE showing electrophoretic mobility shifts of hydroxylamine-ORI upon coupling with **2** and subsequent addition of **6**. For clarity, the gel image is cropped to highlight the relevant bands; the full gel is provided in Figure S8a. (c) Dh (nm) distributions (in intensity) by DLS of hydroxylamine-ORI (top), after reaction with **2** to yield PEG-ORI (middle), and after addition of **6** to restore hydroxylamine-ORI (bottom).

Overall, PEG conjugation to ORI was successfully achieved via the formation of boron-stabilized oxime linkages, while preserving the native morphology of ORI, as confirmed by TEM (Figure S11).

We next examined the dynamic nature of the PEG coating on ORI. PEG-functionalized ORI was incubated at r.t. for 24 h with **6** (10-fold excess relative to **2**). This treatment displaced the PEG chains from the origami surface, as evidenced by the decrease in Dh measured by DLS (Figure 3c) and by AFM (Figure S9). Consistently, AGE analysis showed partial recovery of electrophoretic mobility toward that of the unmodified origami (Figure 3b and S8a), supporting the dynamic behavior of the boron-stabilized oxime bonds illustrated in Figure 3a.

Building on these results, we next explored the dynamic anchoring of ORI to lipid vesicles. We hypothesized that vesicles formulated from phospholipids and relatively hydrophobic derivatives of **1**, such as **3** and **4** (Figure 1b), could form reversible covalent bonds with α-effect amine–labeled ORI, enabling the formation of higher-order dynamic assemblies (Figure S12). To test this, ORI displaying **8** on both faces (Figure S1a) was incubated with large unilamellar vesicles (LUVs) composed of 1-palmitoyl-2-oleoyl-*sn*-glycero-3-phosphocholine (POPC) and 1% (w/w) of **3** or **4.** This led to a pronounced increase in Dh (Figure S13), supporting the ORI-mediated cross-linking of LUVs via iminoboronate conjugation. In contrast, no size change was observed with LUVs containing **10**, a less hydrophobic derivative of **1**, that fails to incorporate into the membrane (Figure S14). This confirms that the observed LUV cross-linking requires the initial anchoring of FPBA-derivatives to the lipid bilayer. Additional control experiments confirmed that anchoring occurs specifically through iminoboronate coupling (Figure S15): no significant changes in Dh were observed when (i) LUVs bearing **4** were incubated with ORI lacking α-effect amine handles, or (ii) ORI displaying **8** was incubated with LUVs lacking FPBA. Importantly, addition of **6** to the ORI–LUV assemblies restored the original Dh of the LUVs within 1 h at r.t. (Figure S13), demonstrating the reversibility of the functionalization. These results further support the dynamic nature of the conjugation at the LUV interface.

Finally, we evaluated the versatility of this strategy using giant unilamellar vesicles (GUVs), which serve as biomimetic models of cell membranes. Atto488-labeled ORI displaying **8** on one face (Figure S1b) was incubated with GUVs composed of 2-dioleoyl-*sn*-glycero-3-phosphocholine (DOPC), Lissamine Rhodamine PE (Liss Rhod) at 1% (mol/mol) for membrane labeling, and 1% (w/w) of **4** to enable anchoring via boron-stabilized oxime formation. Confocal microscopy showed clear colocalization of the Atto488-labeled origami (green channel) with Liss Rhod–labeled GUVs (yellow channel), indicating successful membrane anchoring through iminoboronate coupling (Figure 4a and S16). Addition of **6** resulted in complete loss of the Atto488 signal from the membrane within 30 min (Figure 4b and S17), consistent with dynamic dissociation of the origami. No colocalization was observed for GUVs lacking **4** (Figure 4c and S18), confirming that anchoring is mediated by iminoboronate chemistry. These results demonstrate the efficiency and reversibility of iminoboronate-mediated conjugation and highlight the potential of DCC to enable controllable DNA origami–membrane interactions in synthetic membrane systems. The reversible nature of the iminoboronate linkage, however, also entails intrinsic limitations, as conjugation is governed by thermodynamic equilibrium. Consequently, the degree of functionalization depends on the reaction conditions, including the concentration of the binding partners and the stability of the dynamic covalent interaction. Such equilibrium-dependent characteristics should therefore be considered when designing DNA origami assemblies modified through DCC, but they also provide the key advantage of reversible and externally controllable conjugation.

**Figure 4:**
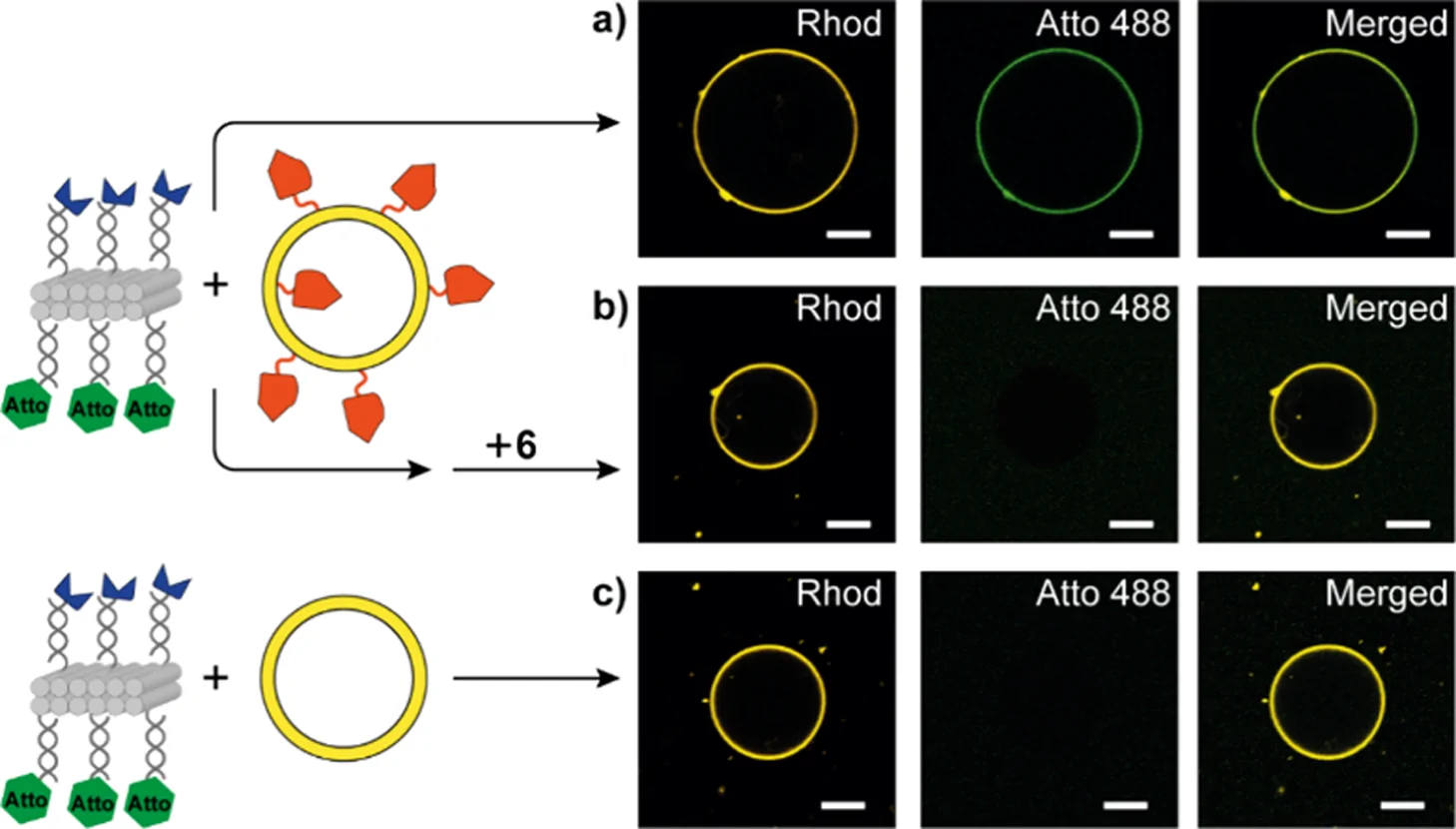
Schematic representation and confocal images showing the dynamic anchoring of DNA origami to GUVs via iminoboronate coupling. Hydroxylamine handles **8** and Atto488 labels on ORI are shown in blue and green, respectively. The presence of the FPBA moiety **4** in the GUV membrane is indicated in orange. (a) Atto488-ORI displaying **8** incubated with FPBA-GUVs and (b) after subsequent incubation with **6**. (c) Atto488-ORI displaying **8** was incubated with GUVs lacking **4**. Liss Rhod (membrane) and Atto488 (ORI) channels are shown in separate columns. Scale bar: 10 μm.

Overall, these results establish iminoboronate chemistry as a general and robust platform for the dynamic functionalization of DNA origami, enabling the modular attachment of polymeric chains and the controllable regulation of DNA–vesicle interactions. More broadly, this reversible chemistry provides a powerful handle to engineer adaptive DNA nanomaterials capable of responding to environmental cues, paving the way toward stimuli-responsive assemblies and reconfigurable nanostructures.

## Supplementary Material





## Notes

Supplementary data (XLSX)
Methods, protocols, instrumentation, materials and supplementary data (PDF)

## References

[1] Madsen M., Gothelf K. V. (2019). Chemistries for DNA Nanotechnology. Chem. Rev..

[2] Jin Y., Yu C., Denman R. J., Zhang W. (2013). Recent Advances in Dynamic Covalent
Chemistry. Chem. Soc. Rev..

[3] Ijäs H., Hakaste I., Shen B., Kostiainen M. A., Linko V. (2019). Reconfigurable DNA Origami Nanocapsule
for PH-Controlled Encapsulation
and Display of Cargo. ACS Nano.

[4] De
Stefano M., Vesterager Gothelf K. (2016). Dynamic Chemistry of Disulfide Terminated
Oligonucleotides in Duplexes and Double-Crossover Tiles. ChemBioChem..

[5] Faiad S., Laurent Q., Asohan J., Brown T., Prinzen A., Sleiman H. F. (2025). Site-Specific Disulfide-Mediated Crosslinking of DNA
Nanocubes for Enhanced Biological Applications. Small Sci..

[6] Kazane S. A., Sok D., Cho E. H., Loressa M., Kuhn P., Schultz P. G., Smider V. V. (2012). Site-Specific
DNA-Antibody Conjugates for Specific
and Sensitive Immuno-PCR. Proc. Natl. Acad.
Sci. U. S. A..

[7] Forget D., Boturyn D., Defrancq E., Lhomme J., Dumy P. (2001). Highly Efficient
Synthesis of Peptide - Oligonucleotide Conjugates: Chemoselective
Oxime and Thiazolidine Formation. Chem. Eur..

[8] Kölmel D. K., Kool E. T. (2017). Oximes and Hydrazones
in Bioconjugation: Mechanism
and Catalysis. Chem. Rev..

[9] Canal-Martín A., Pérez-Fernández R. (2020). Protein-Directed
Dynamic Combinatorial
Chemistry: An Efficient Strategy in Drug Design. ACS Omega.

[10] Kool E. T., Park D.-H., Crisalli P. (2013). Fast Hydrazone Reactants:
Electronic
and Acid/Base Effects Strongly Influence Rate at Biological PH. J. Am. Chem. Soc..

[11] Cal P. M. S. D., Vicente J. B., Pires E., Coelho A. V., Veiros L. F., Cordeiro C., Gois P. M. P. (2012). Iminoboronates:
A New Strategy for
Reversible Protein Modification. J. Am. Chem.
Soc..

[12] Gutiérrez-Moreno N. J., Medrano F., Yatsimirsky A. K. (2012). Schiff Base Formation and Recognition
of Amino Sugars, Aminoglycosides and Biological Polyamines by 2-Formyl
Phenylboronic Acid in Aqueous Solution. Org.
Biomol. Chem..

[13] Haggett J. G., Domaille D. W. (2024). Ortho -Boronic Acid Carbonyl Compounds and Their Applications
in Chemical Biology. Chem. Eur..

[14] Chio T. I., Gu H., Mukherjee K., Tumey L. N., Bane S. L. (2019). Site-Specific Bioconjugation
and Multi-Bioorthogonal Labeling via Rapid Formation of a Boron-Nitrogen
Heterocycle. Bioconjugate Chem..

[15] Dilek O., Lei Z., Mukherjee K., Bane S. (2015). Rapid Formation of a Stable Boron-Nitrogen
Heterocycle in Dilute, Neutral Aqueous Solution for Bioorthogonal
Coupling Reactions. Chem. Commun..

[16] Li K., Weidman C., Gao J. (2018). Dynamic Formation
of Imidazolidino
Boronate Enables Design of Cysteine-Responsive Peptides. Org. Lett..

[17] Schmidt P., Stress C., Gillingham D. (2015). Boronic Acids
Facilitate Rapid Oxime
Condensations at Neutral PH. Chem. Sci..

[18] Cai P., Schneider L. A., Stress C., Gillingham D. (2021). Building Boron
Heterocycles into DNA-Encoded Libraries. Org.
Lett..

[19] Ruiz J., Laga E., Velázquez-Campoy A., del Barrio J. (2026). Dissecting
Binary and Ternary Iminoboronates Assemblies: A Calorimetric Study
of Thermodynamics, Kinetics, and Allosteric Effects. ChemRxiv..

[20] Zhou M., Peng H., Luo S., Jiao K., Guo L., Fan C., Li J. (2025). Functionalization
of Nucleic Acid Molecular Machines
under Physiological Conditions: A Review. ACS
Appl. Bio Mater..

[21] Postigo A., Marcuello C., Verstraeten W., Sarasa S., Walther T., Lostao A., Göpfrich K., del Barrio J., Hernández-Ainsa S. (2025). Folding and
Functionalizing DNA Origami:
A Versatile Approach Using a Reactive Polyamine. J. Am. Chem. Soc..

[22] Ramakrishnan S., Ijäs H., Linko V., Keller A. (2018). Structural Stability
of DNA Origami Nanostructures under Application-Specific Conditions. Comput. Struct. Biotechnol. J..

[23] Li K., Kelly M. A., Gao J. (2019). Biocompatible
Conjugation of Tris
Base to 2-Acetyl and 2-Formyl Phenylboronic Acid. Org. Biomol. Chem..

