# Supplementary material for: Dynamic Covalent Functionalization of DNA Origami via Iminoboronate Chemistry
Source: J Am Chem Soc. 2026 Aug 5;148(32):34033–7. doi: 10.1021/jacs.6c08320 (PMC13495744; doi:10.1021/jacs.6c08320)
Supplement: Supplementary file 1 [file ja6c08320_si_002.pdf]

Supporting Information for

Dynamic Covalent Functionalization of DNA origami *via* Iminoboronate Chemistry

Alejandro Postigo, [a] Enrique Guerreiro, a],¶ Joseba Ruiz,[b],¶] M. Sofía Navarrete-Burgos,[a],¶ William Verstraeten,[c] Kerstin Göpfrich,[c],[d] Silvia Hernández-Ainsa.[a]*and Jesús del Barrio[a]*

[a] Instituto de Nanociencia y Materiales de Aragón (INMA), CSIC-Universidad de Zaragoza, 50009 Zaragoza, Spain

[b] Certest Biotec S.L., San Mateo de Gállego, 50840 Zaragoza, Spain

[c] Heidelberg University, Center for Molecular Biology of Heidelberg University (ZMBH), Berliner Str. 53, 69120 Heidelberg, Germany

[d] Cluster of Excellence SynthImmune, Heidelberg University, 69117 Heidelberg, Germany

E-mails: jdb529@unizar.es, silviamh83@unizar.es
(¶)E.G, J.R. and M.S.N.B. contributed equally.

1. Methods, protocols, instrumentation and materials

1.1. DNA nanostructures design and preparation

Square-shaped, two-layer DNA origami nanostructures were designed using the open-source software caDNAno v0.2. Two variants of the nanostructures were prepared, as schematically represented in Figure S1:
(i) A symmetric DNA origami design containing 96 ssDNA extensions, with 48 protruding from the top face and 48 from the bottom face (Figure S1a). All 96 extensions are 17 nt in length and share an identical sequence. Complete sequence information is provided in Table S1a.
(ii) An asymmetric DNA origami design containing 96 ssDNA extensions of 17 nt each. The 48 extensions on the top face share a common sequence distinct from the sequence shared by the 48 extensions on the bottom face (Figure S1b). Complete sequence information is provided in Table S1b.

Unmodified oligonucleotides were purchased from Macrogen and ThermoFisher with standard desalting purification. Hydrazide-, hydroxylamine- and atto488-modified oligonucleotides were obtained from Biomers. The modified oligonucleotides were supplied at HPLC-purified grade, which includes HPLC purification and confirmation of the purified product by mass spectrometry by the vendor. The scaffold strand p7560 was purchased from Tilibit nanosystems.

Folding of DNA origami nanostructures involved the addition of strands in a fourfold molar excess relative to the p7560 scaffold in a buffer containing 14 mM $MgCl_2$ in 1x TE pH 8.2 (Sigma Aldrich). For fluorescently labelled DNS origami, chemically modified ssDNA strands bearing atto488 fluorophores at their 5' termini were included in the staple mixture at a fourfold molar excess relative to the scaffold. Folding was conducted in a thermocycler (Bio-Rad), employing a 24h protocol, previously used for analogous DNA origami structures (*A. Postigo et al. J. Am. Chem. Soc. 2025, 147, 3919*). Upon completion of the annealing process, samples were maintained at 4°C until further use.

Next, the excess of staple strands was removed by PEG precipitation, followed by spin filtration. For PEG precipitation, assembled samples were mixed 1:1 (v/v) with PEG

buffer (18.75 mM PEG 8 kDa,1x TE, and 0.5 M NaCl) and gently mixed by tube inversion. The mixture was incubated for 30 min at room temperature (rt) and then centrifuged at 16,000 x g for 25 min at rt using a micro centrifuge (ThermoScientific). The supernatant was carefully discarded, and the resulting pellet was resuspended in the initial sample volume with 1x TE supplemented with 12 mM $MgCl_2$, followed by overnight incubation at rt under gentle agitation to ensure complete resuspension. Subsequent purification was conducted by spin filtration using 100 kDa molecular weight cut-off filters (Amicon Ultra-0.5 Centrifugal Filter 100 kDa MWCO Millipore). Prior to centrifugation, each 100 µL of DNA origami was diluted to 400 µL with 1x TE containing 3 mM $MgCl_2$ to avoid aggregation. After spin purification, DNA origami was collected, and its concentration was determined by UV-visible spectroscopy (NanoDrop 2000, Thermo Fisher Scientific). The final $MgCl_2$ concentration was then adjusted to 12 mM.

Chemically modified ssDNA strands bearing hydroxylamine (***8***) or hydrazide (***9***) moieties at their 5’ termini were subsequently added at a twofold molar excess relative to the purified DNA origami and allowed to hybridize with the ssDNA extensions of the DNA origami design, yielding the reactive DNA origami nanostructures used in subsequent experiments.

The correct folding and purification of the DNA origami samples was assessed by agarose gel electrophoresis (AGE). For this purpose, DNA origami samples, at concentrations ranging from 50 to 75 ng/µL and supplemented with gel loading dye (New England Biolabs) were loaded onto a 1% (w/v) agarose gel. Electrophoresis was conducted for 50 min at 80 V in 0.5x Tris-borate-EDTA (TBE) buffer, pH 8.3 (ThermoFisher) supplemented with 11 mM $MgCl_2$. Following electrophoresis, gels were stained with GelRed (Biotium) and visualized under UV transillumination using a GelDoc Go Imaging System (Bio-Rad).

1.2. Synthesis of aldehyde derivatives employed in this study.

1.2.1. Synthesis of compound 3

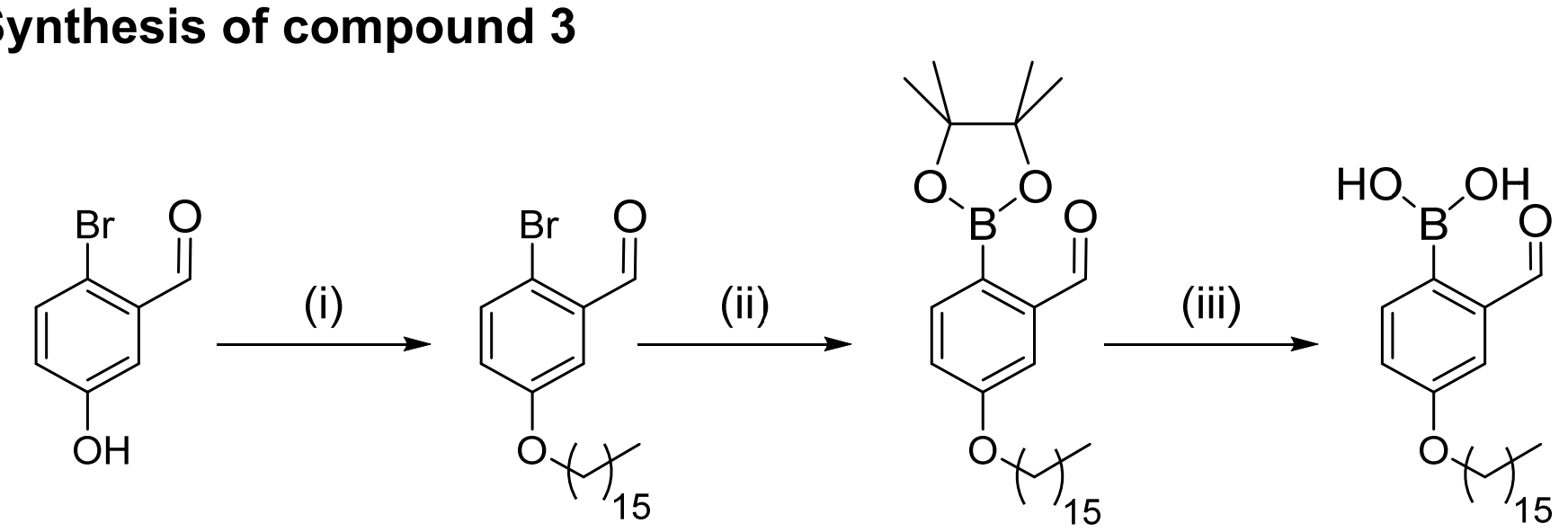

6a **7a** **3**

Scheme S1. Conditions and reagents for the synthesis of compound **3**: (i) 1-bromohexadecane, K_2CO_3, anhydrous DMF, rt to 80 °C, 24 h, 77%; (ii) $B_2(Pin)_2$, SPhos Pd G2, SPhos, anhydrous dioxane, 90 °C, 6 h, 45%; (iii) diethanolamine (DEA), diethyl ether then acid workup, rt, 24 h, 22%

1.2.1.1 Synthesis of 2-bromo-5-(hexadecyloxy)benzaldehyde (6a)

A solution of 1-bromohexadecane (1 eq., 1.7 g, 5.5 mmol) in anhydrous DMF (12 mL) was added to a flask containing 2-bromo-5-hydroxybenzaldehyde (1 eq., 1.1 g, 5.5 mmol) at rt. Potassium carbonate (3 eq., 2.2 g, 16 mmol) was then added, and the reaction mixture was stirred and heated at 80 °C. Reaction progress was monitored by thin-layer chromatography (TLC) using hexane and CH_2Cl_2 (6:4) as the eluent. After 24 h, the reaction mixture was diluted with 100 mL ethyl acetate, filtered by gravity to remove

solids and the organic phase was washed with distilled water (2 × 15 mL), followed by 1 M NaOH and saturated aqueous NaCl solution (brine). The organic phase was dried over $MgSO_4$, filtered, and the solvent was removed under reduced pressure. The crude product was purified by recrystallization in methanol to afford **6a** as an ochre solid. Yield: 77 %. ^{1}H-NMR (400.13 MHz, $CDCl_3$): δ = 10.31 (s, 1H); 7.53-7.49 (m, 1H); 7.41-7.39 (m, 1H); 7.05-7.00 (m, 1H); 3.98 (t, J = 3.6 Hz, 2H); 1.82-1.74 (m, 2H); 1.48-1.40 (m, 2H); 1.26 (s, 24H); 0.88 (t, J = 6.9 Hz, 3H). ^{13}C-NMR (126 MHz, $CDCl_3$) δ = 192.03, 158.99, 134.66, 134.08, 123.66, 117.82, 113.52, 68.79, 32.08, 29.76-29.80, 29.73, 29.69, 29.51, 29.47, 29.17, 26.08, 22.84, 14.27. ν_{max} (FTIR-ATR)/cm^{-1}: 2949, 2916, 2849, 1691, 1589, 1470, 1273, 1236, 827, 754, 671.

1.2.1.2 Synthesis of 5-(hexadecyloxy)-2-(4,4,5,5-tetramethyl-1,3,2-dioxaborolan-2-yl)benzaldehyde (7a)

Compound **6a** (1 eq., 0.86 g, 2.0 mmol), potassium acetate (3 eq., 0.61 g, 6.1 mmol), bis(pinacolato)diboron (3 eq., 1.5 g, 6.1 mmol), SPhos Pd G2 catalyst (0.03 eq., 45 mg, 0.063 mmol), and SPhos ligand (0.06 eq., 56 mg, 0.14 mmol) were dissolved in 20 mL anhydrous dioxane and placed in a Schlenk tube connected to a vacuum line to allow operation under an inert atmosphere. Three vacuum/argon cycles were performed, and the reaction mixture was stirred at 90 °C. Reaction progress was monitored by TLC using a mixture of hexane and CH_2Cl_2 (7:3) as the eluent. After 6 h, the reaction mixture was diluted with 80 mL of ethyl acetate and filtered through Celite® 545. The solvent was removed under reduced pressure, and the crude was redissolved in 40 mL of ethyl acetate. The organic phase was washed with water (3 × 15 mL), dried over anhydrous $MgSO_4$, and filtered by gravity. The solvent was again removed under vacuum, and the crude was purified by flash column chromatography (silica gel, Hexane/ CH_2Cl_2 7:3) affording **7a.** Yield: 45%. ^{1}H-NMR (400.13 MHz, $CDCl_3$): δ = 10.67 (s, 1H); 7.87-7.83 (m, 1H); 7.50-7.46 (m, 1H); 7.13-7.09 (m, 1H); 4.03 (t, *J* = 6.6 Hz, 2H); 1.83-1.75 (m, 2H); 1.49-1.40 (m, 2H), 1.36 (s, 12H); 1.26-1.25 (m, 24H); 0.88 (t, *J* = 6.9 Hz, 3H). ^{13}C-NMR (126 MHz, $CDCl_3$) δ = 195.10, 161.68, 143.71, 138.04, 120.55, 111.06, 84.27, 83.65, 68.36, 32.08, 29.91-29.77, 29.74, 29.71, 29.51, 29.23, 26.11, 25.18, 25.02, 22.84, 14.26. HRMS: m/z (ESI$^+$) calc. for $C_{29}H_{49}BNaO_4^+$ [M+Na]$^+$: 495.362; Obs.: 495.459. ν_{max} (FTIR-ATR)/cm^{-1}: 2920, 2849, 1688, 1593, 1466, 1371, 1275, 1223, 1171, 1148, 845, 831, 721.

1.2.1.3 Synthesis of (2-formyl-4-(hexadecyloxy)phenyl)boronic acid (3)

Diethanolamine (10 eq., 224 mg, 2.1 mmol) was added to a solution of **7a** (1 eq., 106 mg, 0.22 mmol) in diethyl ether (1 mL). The reaction mixture was stirred at rt for 24 h, and the formation of a white precipitate was observed after approximately 30 min. The precipitate was then isolated by vacuum filtration and washed with diethyl ether. The resulting solid was suspended in a mixture of 2 mL of ethyl acetate and 2 mL of aqueous HCl 0.1 M, the mixture was then vigorously stirred at rt for 30 min. Finally, distilled water (18 mL) was added to the reaction mixture, and the aqueous phase was extracted with ethyl acetate (3 × 15 mL). The combined organic phases were washed with saturated aqueous NaCl solution (brine), dried over anhydrous Na_2SO_4, and filtered by gravity. The solvent was removed under reduced pressure to directly afford compound **3.** Yield: 22%. ^{1}H-NMR (400.13 MHz, $CDCl_3$): δ = 9.85 (s, 1H); 8.25-8.22 (m, 1H); 7.44-7.40 (m, 1H); 7.20-7.14 (m, 1H); 4.06 (t, *J* = 6.5 Hz, 2H); 1.87-1.79 (m, 2H); 1.52-1.43 (m, 2H); 1.26 (s, 26H); 0.88 (*t*, *J* = 6.8 Hz, 3H). ^{13}C-NMR (126 MHz, $CDCl_3$) δ = 198.48, 161.45, 141.53, 140.70, 125.48, 118.92, 68.40, 31.93, 29.70-29.66, 29.59, 29.56, 29.37, 29.08, 25.98, 22.70, 14.12. ν_{max} (FTIR-ATR)/cm^{-1}: 3329, 3180, 2916, 2849, 1682, 1599, 1466, 1275, 1250, 1186, 1146, 939, 912, 831, 768, 719.

1.2.2. Synthesis of compound 2.

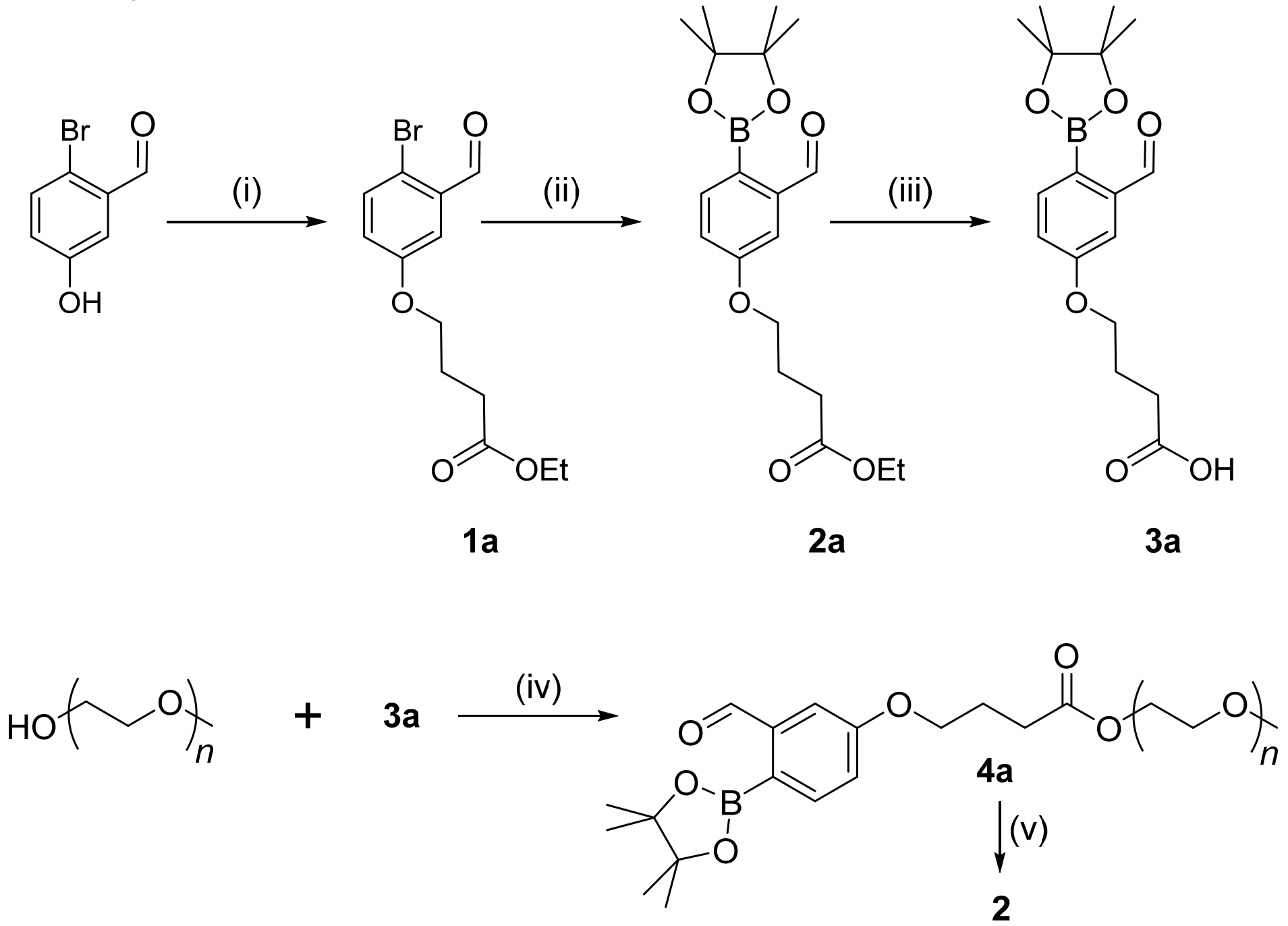

Scheme S2. Conditions and reagents for the synthesis of compound **2**: (i) Ethyl 4-bromobutyrate, NaOH, anhydrous DMF, 100 °C, overnight, 88%; (ii) $B_2(Pin)_2$, KOAc, $PdCl_2$(dppf), anhydrous dioxane, 95 °C, overnight, 78%; (iii) NaOH, EtOH/H_2O, then acid workup; rt, 2 h, 80%; (iv) DMAP, EDC·HCl, anhydrous DCM, 0 °C to rt, 16 h, 74%; (v) $NaIO_4$, THF/H_2O, then aqueous workup.

1.2.2.1 Synthesis of ethyl 4-(4-bromo-3-formylphenoxy)butanoate (1a)

2-bromo-5-hydroxybenzaldehyde (1.0 eq., 7.5 g, 37 mmol) was dissolved in 100 mL of DMF. Ground sodium hydroxide (1.1 eq., 1.7 g, 41 mmol) was added. The mixture was stirred at 100 °C for one hour until sodium hydroxide was dissolved. Ethyl 4-bromobutyrate (1.5 eq., 11 g, 56 mmol) was dissolved in 5 mL of anhydrous DMF and added to the reaction mixture, which was stirred overnight at 100 °C. The reaction crude was neutralized with 1 M HCl and diluted with 100 mL of deionized water. The aqueous phase was extracted with diethyl ether (2 × 200 mL). The organic phases were combined, dried with $MgSO_4$, filtered and the solvent was removed with rotary evaporator to obtain a yellow oil. Column chromatography (silica gel, CH_2Cl_2/Hexane 8:2) was performed to obtain **1a** as a dark yellow oil. Yield: 88%. ^{1}H-NMR (400.13 MHz, $CDCl_3$): δ = 10.28 (s, 1H), 7.50 (d, J = 8.8 Hz, 1H), 7.37 (d, J = 3.2 Hz, 1H), 7.00 (dd, J = 8.8, 3.2 Hz, 1H), 4.13 (q, J = 7.2 Hz, 2H), 4.03 (t, J = 6.2 Hz, 2H), 2.49 (t, J = 7.2 Hz, 2H), 2.11 (m, 2H), 1.24 (t, J = 7.2 Hz, 3H). ^{13}C-NMR (126 MHz, $CDCl_3$) δ = 191.91, 173.10, 158.64, 134.72, 134.09, 123.50, 118.09, 113.55, 67.49, 60.68, 30.78, 24.55, 14.36. HRMS: m/z (ESI$^+$) calc. for $C_{13}H_{15}BrNaO_4$ [M+Na]$^+$: 337.0046; Obs.: 337.0049. ν_{max} (FTIR-ATR)/cm^{-1}: 2980, 2935, 1699, 1685, 1596, 1472, 1373, 1288, 1269, 1221, 1171, 1142, 856, 825, 758, 671.

1.2.2.2 Synthesis of ethyl 4-(3-formyl-4-(4,4,5,5-tetramethyl-1,3,2-dioxaborolan-2-yl)phenoxy)butanoate (2a)

Compound **1a** (1.0 eq., 4.7 g, 15 mmol), bis(pinacolato)diboron (2.0 eq., 7.5 g, 30 mmol), potassium acetate (3.0 eq., 4.4 g, 45 mmol) and [1,1-Bis(diphenylphosphino)ferrocene]dichloropalladium(II) (0.05 eq., 0.55 g, 0.75 mmol) were dissolved in dry dioxane (70 mL) in a Schlenk tube under argon atmosphere. The

solvent was deoxygenated by three freeze-vacuum-argon cycles. The resulting heterogeneous orange mixture was heated to 95 °C and stirred overnight under light protection. The reaction crude was diluted with 150 mL of water and extracted three times with ethyl acetate (3 × 200 ml). The organic phases were combined, dried with $MgSO_4$, filtered and evaporated. The crude was purified by flash column chromatography (silica gel, ethyl acetate/Hexane 1:3) to yield **2a** as a yellow oil. Yield: 78%. ^{1}H-NMR (400.13 MHz, $CDCl_3$): δ = 10.66 (s, 1H), 7.85 (d, *J* = 8.3 Hz, 1H), 7.47 (d, *J* = 2.6 Hz, 1H), 7.10 (dd, *J* = 8.3, 2.6 Hz, 1H), 4.14 (q, *J* = 7.1 Hz, 2H), 4.08 (t, *J* = 6.2 Hz, 2H), 2.51 (t, *J* = 7.3 Hz, 2H), 2.16-2.09 (m, 2H), 1.36 (s, 12H), 1.25 (t, *J* = 7.1 Hz, 3H). ^{13}C NMR (126 MHz, $CDCl_3$) δ = 194.96, 173.18, 161.30, 143.68, 138.09, 120.37, 111.07, 84.28, 67.05, 60.63, 30.85, 24.99, 24.61, 14.35. HRMS: m/z (ESI$^+$) calc. for $C_{19}H_{27}BNaO_6$ $[M+Na]^+$: 385.1796; Obs.: 385.1797. ν_{max} (FTIR-ATR)/cm^{-1}: 2980, 2939, 1730, 1691, 1591, 1466, 1387, 1273, 1231, 1165, 1111, 866, 824, 754.

1.2.2.3 Synthesis of 4-(3-formyl-4-(4,4,5,5-tetramethyl-1,3,2-dioxaborolan-2-yl)phenoxy) butanoic acid (3a)

Compound **2a** (1 eq., 2.0 g, 5.5 mmol) was dissolved in ethanol (22 mL). Sodium hydroxide (10 eq., 2.2 g, 55 mmol), previously dissolved in 2 mL of deionized water, was added dropwise and the reaction mixture was stirred for 2 h at rt. The solution was slowly acidified with 1 M HCl to pH 1. The crude mixture was extracted three times with ethyl acetate (3 × 20 mL), and the organic phase was dried over $MgSO_4$ and filtered. Removal of the solvent under reduced pressure directly afforded **3a** as a brown-white solid. Yield: 80%. ^{1}H-NMR (400.13 MHz, $CDCl_3$): δ = 10.66 (s, 1H), 7.85 (d, *J* = 8.3 Hz, 1H), 7.47 (d, *J* = 2.6 Hz, 1H), 7.10 (dd, *J* = 8.3, 2.6 Hz, 1H), 4.10 (t, *J* = 6.1 Hz, 2H), 2.57 (t, *J* = 7.2 Hz, 2H), 2.16-2.10 (m, 2H), 1.36 (s, 12H). ^{13}C-NMR (126 MHz, $CDCl_3$) δ = 195.03, 178.52, 161.23, 143.68, 138.12, 120.40, 111.08, 84.31, 66.84, 30.48, 25.00, 24.35. HRMS: m/z (ESI$^+$) calc. for $C_{17}H_{23}BNaO_6$ $[M+Na]^+$: 357.1483; Obs.: 357.1474. ν_{max} (FTIR-ATR)/cm^{-1}: 2978, 2937, 1734, 1690, 1597, 1470, 1373, 1273, 1227, 1169, 1144, 849, 827, 787.

1.2.2.4 Synthesis of polymer 2

PEG-OH (Mn ca. 5 kDa, 1.00 g, 0.20 mmol polymer, corresponding to 0.20 mmol hydroxyl groups) was dissolved in anhydrous CH_2Cl_2 (10 mL) under argon. DMAP (2.4 mg, 0.020 mmol, 0.10 eq. relative to OH groups) EDC·HCl (50 mg, 0.26 mmol, 1.3 eq. relative to OH groups) and compound **3a** (74 mg, 0.22 mmol, 1.1 eq.) were added, and the reaction mixture was stirred in an ice–water bath for 20 min. The mixture was then allowed to warm to rt and stirred for 16 h under argon. Water (20 mL) was added, and the mixture was transferred to a separatory funnel. The organic layer was separated, and the aqueous layer was extracted with CH_2Cl_2 (3 × 50 mL). The combined organic layers were dried over anhydrous $MgSO_4$, filtered, and concentrated under reduced pressure. The crude polymer was precipitated into cold diethyl ether (35 mL), collected by filtration, washed with additional cold diethyl ether, and dried under high vacuum overnight to afford compound **4a** as a white to faint yellow solid. Yield: 74%. Such a compound was characterized (see below) and then subjected to oxidative deprotection of the pinacol boronic ester. ^{1}H-NMR (400.13 MHz, $CDCl_3$): δ = 10.64 (s, 1H); 7.84 (d, *J* = 8.3 Hz, 1H); 7.44 (d, *J* = 2.6 Hz, 1H); 7.08 (dd, *J* = 2.6, 8.3 Hz, 1H); 4.23 (t, *J* = 4.8 Hz, 2H); 4.06 (t, *J* = 6.1 Hz, 2H); 3.64 (m, 454H); 3.37 (s, 3H); 2.53 (t, *J* = 7.3 Hz, 2H); 2.16-2.09 (m, 2H); 1.36 (s,12H). ν_{max} (FTIR-ATR)/cm^{-1}: 2879, 2743, 2694,1736, 1689, 1601, 1466, 1360, 1342, 1279, 1242, 1146, 1095, 1061, 960, 841.

Compound **4a** (Mn ca. 5 kDa, 0.50 g, 0.10 mmol polymer) was then dissolved in THF/H_2O (10:1, 5 mL), and sodium periodate (32.0 mg, 0.15 mmol) was added. The reaction mixture was stirred at rt for 20 min, after which HCl (1.0 M aqueous solution, 0.50 mL) was added, and stirring was continued for 2 h. The reaction mixture was transferred to a separatory funnel and extracted with CH_2Cl_2 (3 × 20 mL). The combined organic layers

were dried over anhydrous Na_2SO_4, filtered, and concentrated under reduced pressure. The crude product was redissolved in CH_2Cl_2 (3 mL), precipitated into cold diethyl ether (30 mL), and collected by filtration to afford compound **2** as a white to faint yellow solid, which was used without further characterization.

1.2.3. Synthesis of compound 4

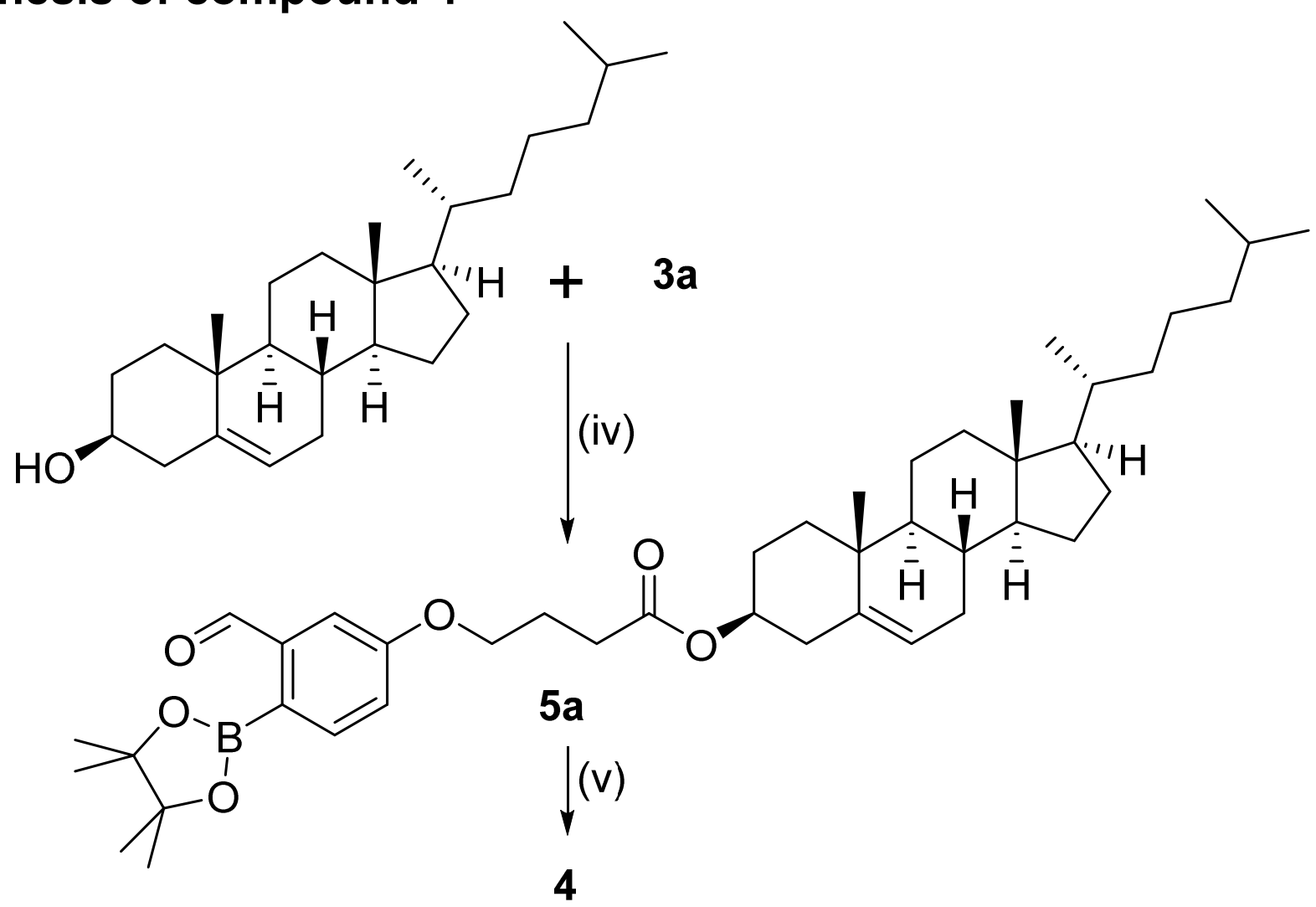

Scheme S3. Conditions and reagents for the synthesis of compound **4**: (iv) DMAP, EDC·HCl, anhydrous DCM, 0 °C to rt, 16 h, 34%; (v) $NaIO_4$, THF/H_2O, then aqueous workup.

Cholesterol (0.387 g, 1.0 mmol) was dissolved in anhydrous CH_2Cl_2 (7 mL) under argon. DMAP (12.2 mg, 0.10 mmol, 0.10 eq. relative to OH groups) EDC·HCl (0.249 mg, 1.3 mmol, 1.3 eq. relative to OH groups) and compound **3a** (0.370 g, 1.1 mmol, 1.1 eq.) were added sequentially. The reaction mixture was stirred in an ice–water bath for 20 min, then allowed to warm to rt and stirred for 16 h under argon. Water (10 mL) was added, and the mixture was transferred to a separatory funnel. The organic layer was separated, and the aqueous layer was extracted with CH_2Cl_2 (3 × 50 mL). The combined organic layers were dried over anhydrous $MgSO_4$, filtered, and concentrated under reduced pressure. The crude was purified by flash column chromatography (silica gel, hexane/ CH_2Cl_2 9:1) affording **5a** as a yellow to brownish solid. Yield: 34%. The compound was characterized (see below) and then subjected to oxidative deprotection of the pinacol boronic ester without further modification. ^{1}H-NMR (400.13 MHz, $CDCl_3$): δ = 10.66 (s, 1H), 7.86 (d, *J* = 8.3 Hz, 1H), 7,48 (d, *J* = 2.6 Hz, 1H), 7,10 (dd, *J* = 8.3, 2.6 Hz, 1H), 5.39-5.35 (m, 1H), 4.69-4.57 (m, 1H), 4.10 (t, *J* = 6.2 Hz, 2H), 2.49 (t, *J* = 7.3 Hz, 2H), 2.33-2.28 (m, 2H), 2.16-2.08 (m, 2H), 2.05-1.96 (m, 3H), 1.94-1.90 (m, 1H), 1.87-1.81 (m, 1H), 1.77-1.70 (m, 1H), 1.62-1.44 (m, 6H), 1.41-1.31 (m, 15H), 1.30-0.95 (m, 14H), 0.92 (d, *J* = 6.6 Hz, 3H), 0.87-0.86 (m, 6H), 0.67 (d, *J* = 0,6 Hz, 3H). ν_{max} (FTIR-ATR)/cm^{-1}: 2883, 2743, 2696, 1734, 1690, 1599, 1466, 1360, 1342, 1279, 1240, 1146, 1095, 1061, 961, 841.

Compound **5a** (140.6 mg, 0.20 mmol) was then dissolved in THF/H_2O (10:1, 3 mL), and sodium periodate (64.0 mg, 0.30 mmol) was added. The reaction mixture was stirred at rt for 20 min. Subsequently, 1.0 M aqueous HCl (0.65 mL) was added, and stirring was continued for 2 h at rt. Distilled water (6 mL) was added to the reaction mixture, and the aqueous phase was extracted with CH_2Cl_2 (3 × 15 mL). The combined organic phases were washed with brine, dried over anhydrous Na_2SO_4, and filtered. Removal of the solvent afforded compound **4** as a brownish solid, which was used directly without further characterization.

1.3. Isothermal Titration Calorimetry

Isothermal titration calorimetry experiments were performed using MicroCal Auto-iTC200 calorimeter (GE Healthcare). ***5***, ***7***, ***8*** or ***9*** compounds were loaded into the sample cell (400 µL) at a concentration of 100 µM in 100 mM phosphate buffer (pH 7.4). A solution of ***1*** at 1 mM, prepared in the same buffer, was loaded into the injection syringe (150 µL). Titration consisted of 19 successive injections of 2 µL each into the sample cell, with an interval of 1800 s between injections, at a constant cell temperature of 25 °C. Data were analyzed using AFFINImeter (Software 4 Science Developments), including the KinITC-ETC module, to directly determine the binding co

nstant, binding enthalpy, and the forward and reverse rate constants.

1.4. DNA nanostructures dynamic functionalisation with PEG-FPBA derivative

For the main functionalization process, ssDNA (***8*** or ***9***) at a concentration of 20 µM, corresponding to 20 µM of either hydroxylamine or hydrazide functional groups, was reacted with the PEG-FPBA derivative (***2***) at either equimolar concentration or a 10-fold molar excess for 24 h at rt in PBS. To evaluate the influence of the reaction conditions on the functionalization efficiency, reactions were performed using different ssDNA concentrations, reaction times, temperatures, pH values, NaCl concentrations, and in the presence of DMEM supplemented with 10% FBS.

For DNA origami nanostructures, samples were prepared at a concentration of 10 nM, corresponding to approximately 1 µM of **8** or **9**, and reacted with **2** at a 100-fold molar excess for 24 h at rt in 0.5x TE buffer supplemented with 6 mM $MgCl_2$.

To assess reversibility of PEG conjugation, ***6*** prepared in either PBS or in 100 mM phosphate buffer (pH 6.0, 7.4 or 8.4), was added to the previously PEG-conjugated samples at either a 1.2-fold molar excess (for ssDNA) or a 10-fold molar excess (for ssDNA ***8*** and DNA origami) relative to the concentration of ***2***. The reaction mixtures were then allowed to proceed for up to 24h at 4, 25 or 37 ºC. Control samples underwent the same procedure, replacing all reagents with equal volumes of PBS.

The efficiency of functionalization and reversibility was assessed using polyacrylamide gel electrophoresis (PAGE) for ssDNA and AGE for DNA origami. For PAGE analysis, 20 ng of each sample was loaded onto a 10% polyacrylamide gel and subjected to electrophoresis at 100 V for 30 min. The gel was prepared in a solution containing 11 mM $MgCl_2$ buffered with 1x TEA buffer pH 8.3 (Thermo Fisher). A Low Molecular Weight or a 100 bp DNA ladder (New England Biolabs) were included as molecular references. The degree of functionalization was quantified by measuring the relative band intensities of functionalized versus non-functionalized DNA strands within the same sample well. For DNA origami, AGE was performed as described in section 1.1.

1.5. DNA origami characterization by Dynamic Light Scattering, Atomic Force Microscopy, and Transmission Electron Microscopy

The hydrodynamic diameter (Dh) of the DNA origami nanostructures both unmodified and after functionalization with ***2*** was determined by DLS using a Zetasizer Nano ZS instrument (Malvern). DNA origami samples were diluted to a final concentration of 10 nM in 1xTE buffer containing 12 mM $MgCl_2$ and transferred to a low-volume disposable cuvette (Malvern). Measurements were conducted at 25 °C, with each dataset representing the average of 5 independent runs, each comprising 10 reads per run. Values in intensity are provided. Data were recorded and analysed by ZS explorer software.

For AFM analysis, 5 µL of the unmodified DNA origami nanostructures, after conjugation with **2**, and subsequent reversion with **6** were deposited at a concentration of 2 nM in 10 mM $MgCl_2$, 1x TE onto freshly cleaved mica and allowed to adsorb for 10 min. The samples were then rinsed three times with filtered Milli-Q water, excess liquid was gently removed with a tissue, and the surface was slowly dried under a soft nitrogen air stream for 3 min. Imaging was performed using a Veeco-Bruker Multimode 8 AFM with Tap 150Al-G Soft Tapping mode AFM probes with aluminum reflective coating (BudgetSensors), in soft tapping mode in air. The same AFM probe was used for all measurements to minimize variability in height parameters. Raw AFM images were processed using Gwyddion 2.60. All AFM images were treated consistently following the same workflow and identical processing parameters. Mean values and SD of the heights for DNA origami samples were calculated from 50 particles each.

Transmission electron microscopy (TEM) was employed to visualize the morphology and structural integrity of DNA origami nanostructures, both unmodified and after functionalization with ***2***.
For sample preparation, a 10 µL droplet of DNA origami solution (10 nM in 1x TE containing 12 mM $MgCl_2$) was placed onto a piece of Parafilm within a covered Petri dish. Subsequently, freshly incandescently discharged (30s, 15 mA) carbon-coated 200-mesh copper grids (Agar Scientific Supplies) were incubated over the sample drops for 5 min to allow adsorption of the nanostructures. For staining, 1% aqueous uranyl acetate was added, drop by drop, with each drop removed using a tissue to clean while staining. The final drop was incubated for 1 min, bringing the total staining volume to 50 µL. Finally, the grids were stored in a grid box in a desiccator until further observation using a Tecnai T20 microscope (Thermo Fisher Scientific) at 200 kV.

1.6. DNA origami dynamic attachment to LUVs

Large unilamellar vesicles (LUVs) were prepared by thin-film hydration followed by extrusion. Lipids dissolved in chloroform were mixed to obtain either non-functionalized vesicles composed of 100% 1-palmitoyl-2-oleoyl-sn-glycero-3-phosphocholine (POPC), or FPBA-functionalized vesicles containing 99% POPC and 1% (w/w) ***3***, ***4***, or ***10***. The solvent was removed under reduced pressure to form a thin lipid film, which was subsequently hydrated in PBS to a final lipid concentration of 2 mM. The resulting multilamellar vesicles were extruded 21 times through Whatman 19 mm NUC polycarbonate membrane, with 0.2 µm pore size, using an Avanti Mini-Extruder, yielding LUVs with an average Dh of approximately 200 nm according to DLS. Extruded vesicles were stored at 4 °C and used within the next 3 days.

For conjugation experiments, 25 µL of DNA origami at 10 nM was mixed with 25 µL of LUVs (2 mM lipid concentration) and incubated for 24 h at rt. DNA origami symmetric design (Figure S1a) was employed in these assays bearing 48 hydroxylamine groups on each side. Equivalent design but lacking hydroxylamine groups was used as control. To evaluate reversibility, ***6*** (5 mM final concentration) was added to the pre-formed DNA origami-LUV conjugates, and samples were analyzed by DLS after 1 h and 24 h of incubation at rt.

1.7. Attachment of DNA origami to GUVs and reversibility with 6

Preparation of GUVs

Giant unilamellar vesicles (GUVs) were generated using the electroformation method facilitated by a VesiclePrepPro device (Nanion Technologies GmbH). Two distinct types of GUVs were produced:

(i) Chol-FPBA-containing GUVs: composed of 94 % 1,2-dioleoyl-sn-glycero-3-phosphocholine (DOPC), 1 % 18:1 Lissamine Rhodamine PE (LissRhod PE), and 5% of ***4***.
(ii) Control non-modified GUVs, composed of 99% DOPC and 1% LissRhod PE.

For GUVs electroformation, 40µL of a 2 mM lipid mixture in $CHCl_3$ was homogeneously spread onto the conductive side of an indium tin oxide (ITO) coated glass cover slide (Visiontek Systems Ltd) using a clean cover slide to ensure a uniform lipid film. The lipid-coated ITO slide was then subsequently placed under vacuum for at least 30 min to ensure complete solvent evaporation. Subsequently, an 18 mm diameter rubber ring was positioned on the lipid-coated ITO slide and filled with 275 µL of 200 mM sucrose solution (Sigma-Aldrich). The chamber was sealed by placing a second ITO-coated slide on top of the spacer, forming a closed electroformation cell.

The assembled chamber was inserted into the VesiclePrepPro and subjected to a programmable AC-current with an amplitude of 3 V and a frequency of 5 Hz for 2 h at 37°C. GUVs were promptly collected after formation, diluted 1:6 with 200 mM glucose solution and stored at 4°C for up to 2 days.

Incubation of GUVs with DNA origami

For conjugation assays, GUVs were incubated with DNA origami structures functionalized with 48 hydroxylamine groups ***8*** and 48 fluorescently labelled with Atto 488 (asymmetric design, Figure S1b). The reaction was carried out for 24 h at rt by mixing 2.5 µL DNA origami (20 nM in 1x TE with 12 mM $MgCl_2$), 10 µL of diluted GUVs (modified with ***4***, or non-modified as control), 2.5 µL of 100 mM NaCl, and 5 µL of 70 mM glucose to a final volume of 20 µL.

For reversibility studies, ***6*** was added post-incubation at a final concentration of 5 mM (corresponding to the addition of 2 µL of a 50 mM of ***6*** solution). The samples were observed thereafter.

Confocal imaging

GUVs were imaged in a custom-built observation chamber, which was coated with 50 mg/mL Polyvinyl-alcohol (Sigma-Aldrich) for 5 min to prevent vesicle adhesion and fusion with the glass surface.

Imaging was conducted using an LSM 900 Zeiss confocal fluorescence microscope (Carl Zeiss AG). The pinhole aperture was set to one Airy Unit, and experiments were performed at rt. Images were acquired using a 20x objective (Plan-Apochromat 20x/0.8 M27, Carl Zeiss AG). Subsequent image processing and analysis was carried out using the ZEN 3.4 software (Carl Zeiss AG).

2. Supplementary figures and tables

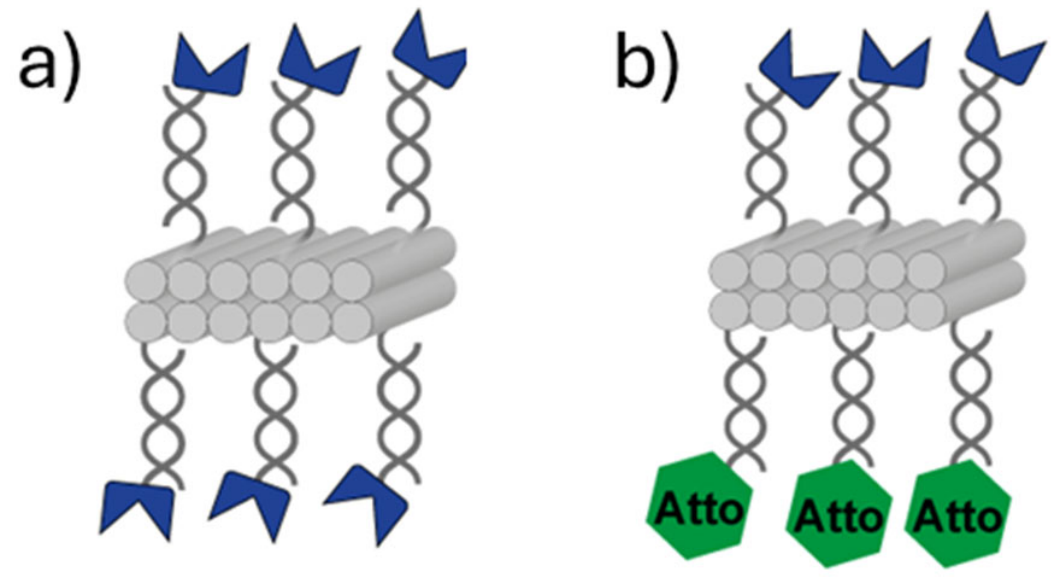

Figure S1. Sideview representation of a) symmetric and b) asymmetric DNA origami designs. Each design contains 48 extensions per side. For clarity, only three extensions are shown in the schematics. Hydroxylamine or hydrazide groups are depicted in blue, whereas Atto488 fluorophores are represented in green.

Table S1a. Strands used to fold the symmetric version of the DNA origami design (Figure S1a) bearing either 96 hydroxylamine or 96 hydrazide groups (48 on each side).

Name	Sequence
Hydroxylamine-strand (**8**)	**/Hydroxylamine/** AAGATCCATAGGCGATA
Hydrazide-strand (**9**)	**/Hydrazide/** AAGATCCATAGGCGATA
Extension A & C (extA & ext C)	TATCGCCTATGGATCTT
Core1	CAGTTTGGAACAAGAGTCCACTAAATCAAAATACAAACTTATGGGAT
Core2	TTCACCGCCTGGCCCTGAGAGAGCACCAGTGAGCGGAGTTATCGGTT
Core3	AATCGCCATATTTAACAACGCCGTATAAAGGCGCCTGTTATCATTC
Core4	GCTTAATTGCTCATTTTTGATTCAAAAGTCATTGCCTGAGAGT
Core5	TCAAAAAGATTTCAGAAGCTCGATGAATAAAATTCGCATTAAA
Core6	AATGTTTAGACTGCCAGAGATTTTTTAGATTGACCGTAATGGG
Core7	ATGAAAATAGGATTTTTTAAAATTAAGATAGCTTAGATTAAG
Core8	AATACCACATTATTCATCAGATGGGCGCATTCGCCATTCAGGC
Core9	AAGAAACAATAGTTAAGCGTAGATTTCAAAAGAAGATGATGA
Core10	AGACCAGGCGCGAGGACAGTTCTAAGTTCTATGATACCGACAG
Core11	AAGGTAAATACATTCAACGAATTGAGGTTATTAATTTTAAAA
Core12	CGCGAAACAAAACCCCCAGAAGCAACTGTGCTTGTTACCTCGA
Core13	TCAGTAGCGAACCATCGACGAACGAAATATCTGGTCAGTTGG
Core14	CGGCTACAGAGGCATCGGAGGGTACCGAGTGAGCTAACTCACA
Core15	ACCCTCAGAAAACCGCCTTTTACATTACTGATAGCCCTAAAA
Core16	CGCAGTCTCTTCATTAAAATTAACCGTACATTTTGACGCTCA
Core17	CGCCACCCTCAGAACCGCCACGCGGGGTTAAAGCGAAGCTAGGGC
Core18	AAATCAAGTTTTTTGGCGGGGAAAAGAGGGTTTGAAAGTA
Core19	GGGCGATGGCCCACTAAAGGGAAGTTGCTCAGAGAAGGAT
Core20	TCCAACGTCAAAGGGCTGGTGGTTACAGCCCTTCTTTCCA
Core21	GAGCCCCCGATTTAGAGCTTGAGGTCGAGGTACTCAGG
Core22	GAGAAAGGCGTGAACCAAC
Core23	CTGTTTGAGAAAAACCCCACCACCCTCATTTT
Core24	CCCTTATATTAAAGAACAATAGGA
Core25	ACCACCACGCACGTATATAAACAGTAAGCGTC
Core26extA	AGGAAAAAACACGACCCACCGGAATTTAGCGTTATCGCCTATGGATCTT
Core27extA	CCAGAACACAACAGAGTTAGCGTTGCATTTTCTATCGCCTATGGATCTT
Core28extA	TGAAATTGCTGACCTCTTAAACGGACCTAAAATATCGCCTATGGATCTT
Core29extA	TTCGTAATGGGCTTAAGACTAAAGAAAACACTTATCGCCTATGGATCTT
Core30extA	CACCAGTCCGCTCATGCTCAGAGCACAAACAATATCGCCTATGGATCTT
Core31extA	ATTCTGGCATATTACCGAACCACCGAGGCAGGTATCGCCTATGGATCTT
Core32extA	TCCGAACTTTATCCGCTGCAGGGACCGATATATATCGCCTATGGATCTT
Core33extA	AGTAAACACATGGTCACGTCACCCGACAATGATATCGCCTATGGATCTT
Core34extA	TGGCTATTAAACAGAGAAGGCCGGATTAAAGGTATCGCCTATGGATCTT
Core35extA	AAGAATACGCCTGCAACAAAATCAGAGCCATTTATCGCCTATGGATCTT
Core36extA	CGCATTTCCCAAAATACGAAATCCGGCGCAGATATCGCCTATGGATCTT
Core37extA	GGCACGAACTCGCCCTGAGATTTGCTGACCAATATCGCCTATGGATCTT
Core38extA	AGAAGATAAGTCTTTAAGTTTGCCCCAGAGCCTATCGCCTATGGATCTT
Core39extA	TTAACACCGTGGCACATTTCATCGTGCCATCTTATCGCCTATGGATCTT
Core40extA	ATGTCCCGACATAAATAGGCACCAGTAAAATATATCGCCTATGGATCTT
Core41extA	TATTTACGTATAGGGGAATACACTACTTTTTCTATCGCCTATGGATCTT
Core42extA	CCTCAAATATCTTTAGCCAGCGCCTAAAGGTGTATCGCCTATGGATCTT
Core43extA	AATGAAAAGAGCCGTCGTCACAATGACACCACTATCGCCTATGGATCTT
Core44extA	CTGAATTGACGCCAGGAACCGGATTCATTCAGTATCGCCTATGGATCTT
Core45extA	ATTCATGCAAACGACGGCTGACCTAAACACCATATCGCCTATGGATCTT
Core46extA	TCTAAAATATCAAACCAATTATTCAAACGTCATATCGCCTATGGATCTT
Core47extA	ATAGATTAATCTAAAGACCGACTTCCAGTAGCTATCGCCTATGGATCTT
Core48extA	GTTGGGTATCAACCTTCGGAACGAGCGACCTGTATCGCCTATGGATCTT
Core49extA	ACGTTGTAGCACGACTGAACCGAATATCATCGTATCGCCTATGGATCTT
Core50extA	TCGTATTAGAAGGAGCATACCCAATCTTACCGTATCGCCTATGGATCTT

Core51extA	AAGTATTATATCAGATAAGGAAACCAGATAGCTATCGCCTATGGATCTT
Core52extA	GGATGTGCGCCAGCTTTGGCTCATACGTTAATTATCGCCTATGGATCTT
Core53extA	CTCTTCGCAAACCAGGATTGTGAATATTACAGTATCGCCTATGGATCTT
Core54extA	AACCACCAAATCCTTTTACATACAAAAGACAATATCGCCTATGGATCTT
Core55extA	TTCCTGATGACTTTACAACGCAAACAATAGAATATCGCCTATGGATCTT
Core56extA	GCACTCCATGCAAGGCAAAGCTGCATTCATTATATCGCCTATGGATCTT
Core57extA	GGTGCCGGTATTACGCCTGACGAGTCATCAAGTATCGCCTATGGATCTT
Core58extA	GGGTTAGAATGAATATCAGAGAGAAATAACATTATCGCCTATGGATCTT
Core59extA	ATTGTTTGGGGAGAAAACAAAGTCGGGAGAATTATCGCCTATGGATCTT
Core60extA	CAGTATCGTGTGAGCGGCATAGTAACGACGATTATCGCCTATGGATCTT
Core61extA	CCGTGCATGTGGGAACGCAGATACTTTGCAAATATCGCCTATGGATCTT
Core62extA	AACGTCAGACCTACCAAATAGCTAAAGAACTGTATCGCCTATGGATCTT
Core63extA	TTTACATCGATTATACAAAGTAAGCGAGGAAATATCGCCTATGGATCTT
Core64extA	ACATTAAAGCCTCAGGAAAAATCTTATACCAGTATCGCCTATGGATCTT
Core65extA	GATTCTCCCTGCCAGTAACAACATTTACCTTATATCGCCTATGGATCTT
Core66extA	AATTATTCCATTTGAATTTATCCCCTACAATTTATCGCCTATGGATCTT
Core67extA	GAATACCAACATAAATTTTGCCAGAACGAGCGTATCGCCTATGGATCTT
Core68extA	TTCCTGTAGAAGATTGATTGAATCAGAATGACTATCGCCTATGGATCTT
Core69extA	GAACGCCAAACGTTAAGTCCAATAACCCTGACTATCGCCTATGGATCTT
Core70extA	AACAATTTATTTCAATACAGAGAGTAACCCACTATCGCCTATGGATCTT
Core71extA	GAAACAGTAGTTACAACATTAGACAGAGGGTATATCGCCTATGGATCTT
Core72extA	AAAAACAGGCCAGCTTTTTACCAGAGAGCAACTATCGCCTATGGATCTT
Core73extA	AAATTGTATCAAAAATGAGAGGCTATAACGCCTATCGCCTATGGATCTT
Core74	ATTGTTTGGGGAGAAAACAAAGTCGGGAGAAT
Core75	CAGTATCGTGTGAGCGGCATAGTAACGACGAT
Core76	CCGTGCATGTGGGAACGCAGATACTTTGCAAA
Core77	AACGTCAGACCTACCAAATAGCTAAAGAACTG
Core78	TTTACATCGATTATACAAAGTAAGCGAGGAAA
Core79	ACATTAAAGCCTCAGGAAAAATCTTATACCAG
Core80	GATTCTCCCTGCCAGTAACAACATTTACCTTA
Core81	AATTATTCCATTTGAATTTATCCCCTACAATT
Core82	GAATACCAACATAAATTTTGCCAGAACGAGCG
Core83	TTCCTGTAGAAGATTGATTGAATCAGAATGAC
Core84	GAACGCCAAACGTTAAGTCCAATAACCCTGAC
Core85	AACAATTTATTTCAATACAGAGAGTAACCCAC
Core86	GAAACAGTAGTTACAACATTAGACAGAGGGTA
Core87	AAAAACAGGCCAGCTTTTTACCAGAGAGCAAC
Core88	AAATTGTATCAAAAATGAGAGGCTATAACGCC
Core89	CCTTAGAACTGAGAGATTTTAGCGATTTTCAT
Core90	TTCTGTAAGGTTGGGTGGCTTATCATAGCAAG
Core91	GGTTGATAATGCCGGATCGAGCTTAGGTCAGG
Core92	GTAAAACTCTACAAAGGCCCGAAACCTTTTGA
Core93	TCATAGGTTCCTTGAAGCACCCAGAATCCAAA
Core94	CCGGCTTAATCGTCGCCCAACGCTTTACAAAA
Core95	ATAAATTAATCAGAAAAGAAAACGCCCCTCAA
Core96	TGAGAGATAGCATGTCAGGTCTTTCTGCGGAA
Core97	AAACTTTTACCGACCGTCTTTCCTTTATCAAC
Core98	CAAATCCAAGAATAAAGAGCATGTAAATAATA
Core99	TGATATTCTAAAAATTCTAAAGTATGATTCCC
Core100	AAGGCCGGATGCAATGTGCTGTAGGTTTGACC
Core101	TTTGAAATTCAAATATCCGTTTTTAACCTCCC
Core102	GTTAAATAATCGCAAGCGCGCCCACGGTATTC
Core103	CGCAAGGAAACCGTTCACTCCAACCAAAGCGA
Core104	TATTTTAAAGACAGTCTAATTGCTGACTTCAA
Core105	TCTTACCAAACATGTACCAGACGA
Core106	GCCTGTTTCCAGTAATAAAGTACC
Core107	GCGGGAGAACATCCAAGCGAGCTG
Core108	ACCAAAAAAATTAGCATAACCTGT
Core109	AAAGCTAAAATGTGTAGG
Core110	AGAGGCATTTTCGAGAGTATCATAACAAGAAAGAAACCA
Core111	AATAGTAGTAGCATTAAGCCTTTAATAACAGTCGGTGTCT
Core112	ACAGGCAAGGCAAAGCATTATGATAGATTTACTCAACAT
Core113	CGACAATAAACAACATTGCAGAACCCAACGCTTTTAATGG
Core114	GACAAAAGGTAAAGTAGTCCTGATGCGTTATAAGGC
Core115	AAAAGGTGGCATCAATCATTCCATTTTATTACTCATACAA
Core116	TTAGCTATATTTTCAGAACGAGCCCTGTACCTCATA
Core117extC	GACTTGCGGCTATTTTAACATAGCTTACATTTTATCGCCTATGGATCTT
Core118extC	TAAGAACAATCTTATATTAATTTTAATGTATCGCCTATGGATCTT
Core119extC	ACCAGACCAACAGTTCAGCTGAATGTGAGCCCTATCGCCTATGGATCTT
Core120extC	ATATCGCAAAAATCAATCATAAATATTTTATCGCCTATGGATCTT
Core121	CAAGAACGCAAGCAAGATTTTAGTTATCAAAA
Core122	ATCAATATCATTACACAAAGATTAACCT
Core123	GGAAGTTTGGAAGCAATAGGTAAATATATCTG
Core124	GTTTTAAGTACCTTAAATCACCTATTTT
Core125	TCATCGAGAAGGTATTAAGACCTAAACAACAGTA
Core126	CGTAGGAAATCGGCTGTGTGATAAATACAAAT
Core127extC	TAAGAAACCAGCCTTTTACCTGAGTCAGGTTTTATCGCCTATGGATCTT
Core128extC	TAAACAGGGAAGCGAATCGCGAGTACCTTATCGCCTATGGATCTT
Core129extC	ATGCTTTAAACCCTCGTCAGCCTGGATTCTCATATCGCCTATGGATCTT
Core130extC	TCGTCATAAATAGCAATTCGCCCCGTCGTATCGCCTATGGATCTT
Core131	TAAGAACAATCTTATATTAATTTTAATG

Core132	ACCAGACCAACAGTTCAGCTGAATGTGAGCCC
Core133extC	AAAAACAGCCATATTATTACCTTTTAATTTTCTATCGCCTATGGATCTT
Core134extC	TAACTGAAGAGCCTAACAATATATAACCTTGCTATCGCCTATGGATCTT
Core135extC	AAAAACCAAAATATTCTATAAGCATGTACCCCTATCGCCTATGGATCTT
Core136extC	AGAAGTTTTGGATAGCTATTTTGTCGGTAATCTATCGCCTATGGATCTT
Core137extC	AAGAATTGGAAATAGCTATCAAAAAACAAAGATATCGCCTATGGATCTT
Core138extC	ATTGAGCTTTAAGATTCTGAACATCATATATCGCCTATGGATCTT
Core139extC	ACTATCATTTGGGAAGAAGCAATATTCATATCTATCGCCTATGGATCTT
Core140extC	AAAAGGACTAACGGTTGAGGGCGCTTCTTATCGCCTATGGATCTT
Core141	ATGCTTTAAACCCTCGTCAGCCTGGATTCTCA
Core142	TCGTCATAAATAGCAATTCGCCCCGTCG
Core143extC	AAGCCCTTGCTAATATACAGTAACCAGAGGCGTATCGCCTATGGATCTT
Core144extC	CGAACAAACACCCTGACAATAACGATTGCTTTTATCGCCTATGGATCTT
Core145extC	AAAACGAAATTACGAGAGTAACAAGTCTGGCCTATCGCCTATGGATCTT
Core146extC	GTAGAAAGCAACTAATAAACGGCGACCAATAGTATCGCCTATGGATCTT
Core147extC	GCATGATTGTAGAAAAGCCCGAACGAAGGTTATATCGCCTATGGATCTT
Core148extC	CGCAATATAAAAGAAAACAATACAACTATATCGCCTATGGATCTT
Core149extC	TCAGGACGAACGTAACGATTTTGAATACATAATATCGCCTATGGATCTT
Core150extC	TGCGATTGCTTGCCCAGCTGGAGTCACGTATCGCCTATGGATCTT
Core151	AAGCCCTTGCTAATATACAGTAACCAGAGGCG
Core152	CGAACAAACACCCTGACAATAACGATTGCTTT
Core153extC	GCAACATAATAACGGAGGAATTATTAATGGAATATCGCCTATGGATCTT
Core154extC	GGAATAAGGTTACCAGGATGGCAATAATCCTGTATCGCCTATGGATCTT
Core155extC	TGAATAAGTTAAGAACTCCGGCACGACGACGATATCGCCTATGGATCTT
Core156extC	GAACGAGTCTTTAATCCAAAGCGCCATCGTAATATCGCCTATGGATCTT
Core157extC	AAGGGCGATTGACGGACTCAATCACCACCAGCTATCGCCTATGGATCTT
Core158extC	AATTCATCACCGTCCATCACCGTCAGTATATCGCCTATGGATCTT
Core159extC	CCCAAATCTACTTAGCATGAGAGCCGCTGACATATCGCCTATGGATCTT
Core160extC	AGTAATCCATAAGGTAAGTGTTCTAATCTATCGCCTATGGATCTT
Core161	GCAACATAATAACGGAGGAATTATTAATGGAA
Core162	GGAATAAGGTTACCAGGATGGCAATAATCCTG
Core163extC	TGAATTATATGGTTTAGAGCACTATCGACAACTATCGCCTATGGATCTT
Core164extC	TGGGAATTTTTATTTTAATAGATAGGATTTAGTATCGCCTATGGATCTT
Core165extC	CGGTCAATTTGACAAGGTTTTCCCCGAAAGGGTATCGCCTATGGATCTT
Core166extC	CTTTGAAAATAGGCTGGCCAGTGCGTGCGGGCTATCGCCTATGGATCTT
Core167extC	CCAATGAACAGAATCAATGCGCGAGGCAGATTTATCGCCTATGGATCTT
Core168extC	ACCATTAAGCGCGTGACAATAAAGGGACTATCGCCTATGGATCTT
Core169extC	CTCCATGTCACTACGACATGACCTCTTCTTTCTATCGCCTATGGATCTT
Core170extC	CCTGATAGCAAAAGCCTTGAATGTAATGTATCGCCTATGGATCTT
Core171	CGGTCAATTTGACAAGGTTTTCCCCGAAAGGG
Core172	CTTTGAAAATAGGCTGGCCAGTGCGTGCGGGC
Core173extC	CAGACTGTCCATTAGCGTGAGGCGTTGCTGAATATCGCCTATGGATCTT
Core174extC	GGTCATAGAGAGCCAGCAGTGCCACAGCAGCATATCGCCTATGGATCTT
Core175extC	CGAAAGAGAATTGTGTACCCCGCTCCTTAGTGTATCGCCTATGGATCTT
Core176extC	CATCTTTGGTACAACGGGAGTGACGGTTGTGATATCGCCTATGGATCTT
Core177	CAGACTGTCCATTAGCGTGAGGCGTTGCTGAA
Core178	GGTCATAGAGAGCCAGCAGTGCCACAGCAGCA
Core179	CGAAAGAGAATTGTGTACCCCGCTCCTTAGTG
Core180	CATCTTTGGTACAACGGGAGTGACGGTTGTGA
Core181	ACCACCGGCCGCCACCGAAATACCTTGTAGCA
Core182	TTTCATAGCCACCAGCCAGCCTCACTTG
Core183extC	TCAGAGCCATCAAAATAGTAATAATTTTTGAATATCGCCTATGGATCTT
Core184extC	CGCCGCCACCCCCTTAATAGAACCGAAAGCGTTATCGCCTATGGATCTT
Core185extC	CCGCTTTTAGTTTCCACTGGTTGGTCGGCTGATATCGCCTATGGATCTT
Core186extC	GAAAGACAGCTTTGAGGCTACGTGCGTCGGTGTATCGCCTATGGATCTT
Core187	CCGCTTTTAGTTTCCACTGGTTGGTCGGCTGA
Core188	GAAAGACAGCTTTGAGGCTACGTGCGTCGGTG
Core189	ATAAATCCGAATTTACATCAGTGAATGGTTGC
Core190	TCAGACGCTTTTGAGTACGCCTTCCTCG
Core191	TTCGGTCGACGTTGAAGGGGCCGAAGGAGAGA
Core192	CAACAACAGGAGCCCCAGCTGGGTGGTT
Core193	AAACAGCTTGATGCTTTCGCTGCCCGCAACAGCT
Core194	ATACATGGATTGGCCTTAATAACAATTGCAAC
Core195	TGGTAATAGCATTGACCAAACTATGGTAATAT
Core196	GCTCCAAACATCGCCCAGCCGGAATTCCTGTG
Core197	TATCAGCTTACCGATAGCCTAATGAGCTCGAA
Core198	CCCTGCCTCTATTATTCGCTTAATGCGCCGCT
Core199	AGTAACACTGAGACGTCACGCACGTGGC
Core200	ATTTTTTCATCTAAAGAAACGGGCAGGAGATC
Core201	AAAGGAATAAATGAAGCGGTCGGCAAAAT
Core202	TTAAGAGGGTGCCCGTAACGTGCTAGAATCCT
Core203	TAGGATTAAGTTTTAAAGCTAAACTTAAAGGG
Core204	GACGTTAGCAACTAAAGGCGCCAGCATTAATG
Core205	TTTGCTAAACAGTTTCAGACGGGCTTTCCAGT
Core206	TATAGCCCTATCACCGTGCCGTAAAGCACT
Core207	GGATAAGCCCTCAGATCACCC
Core208	GCGTAACGCCTCAGAGGTCTATCA
Core209	TGTAGCAGCAAGCCCGTGGAC
Core210	AGGTTTAGTACCGCCATGCCGTCGGCCGGCGATGCGCGTA
Core211	CAGGGATATTCCACAGCCGAAATCCACGCTGG
Core212	ACCCATGTACCGTAACGTCACCAGGAATAGCCCGAGATA

Table S1b. Strands used to fold the asymmetric version of the DNA origami design Figure S1b), bearing 48 hydroxylamine groups on one side and 48 Atto488 fluorophores on the other.

Name	Sequence
Hydroxylamine-strand (**8**)	**/Hydroxylamine/** AAGATCCATAGGCGATA
Atto488-strand	**/atto488/** AGCCCACATTGAATGT
Extension B (ext B)	TACATTCAATGTGGGCT
Extension C (ext C)	TATCGCCTATGGATCTT
Core1	CAGTTTGGAACAAGAGTCCACTAAATCAAAATACAAACTTATGGGAT
Core2	TTCACCGCCTGGCCCTGAGAGAGCACCAGTGAGCGGAGTTATCGGTT
Core3	AATCGCCATATTTAACAACGCCGTATAAAGGCGCCTGTTATCATTC
Core4	GCTTAATTGCTCATTTTTGATTCAAAAGTCATTGCCTGAGAGT
Core5	TCAAAAAGATTTCAGAAGCTCGATGAATAAAATTCGCATTAAA
Core6	AATGTTTAGACTGCCAGAGATTTTTTAGATTGACCGTAATGGG
Core7	ATGAAAATAGGATTTTTTAAAATTAAGATAGCTTAGATTAAG
Core8	AATACCACATTATTCATCAGATGGGCGCATTCGCCATTCAGGC
Core9	AAGAAACAATAGTTAAGCGTAGATTTCAAAAGAAGATGATGA
Core10	AGACCAGGCGCGAGGACAGTTCTAAGTTCTATGATACCGACAG
Core11	AAGGTAAATACATTCAACGAATTGAGGTTATTAATTTTAAAA
Core12	CGCGAAACAAAACCCCCAGAAGCAACTGTGCTTGTTACCTCGA
Core13	TCAGTAGCGAACCATCGACGAACGAAATATCTGGTCAGTTGG
Core14	CGGCTACAGAGGCATCGGAGGGTACCGAGTGAGCTAACTCACA
Core15	ACCCTCAGAAAACCGCCTTTTACATTACTGATAGCCCTAAAA
Core16	CGCAGTCTCTTCATTAAAATTAACCGTACATTTTGACGCTCA
Core17	CGCCACCCTCAGAACCGCCACGCGGGGTTAAAGCGAAGCTAGGGC
Core18	AAATCAAGTTTTTTGGCGGGGAAAAGAGGGTTTGAAAGTA
Core19	GGGCGATGGCCCACTAAAGGGAAGTTGCTCAGAGAAGGAT
Core20	TCCAACGTCAAAGGGCTGGTGGTTACAGCCCTTCTTTCCA
Core21	GAGCCCCCGATTTAGAGCTTGAGGTCGAGGTACTCAGG
Core22	GAGAAAGGCGTGAACCAAC
Core23	CTGTTTGAGAAAAACCCCACCACCCTCATTTT
Core24	CCCTTATATTAAAGAACAATAGGA
Core25	ACCACCACGCACGTATATAAACAGTAAGCGTC
Core26extB	AGGAAAAAAACACGACCCACCGGAATTTAGCGTTACATTCAATGTGGGCT
Core27extB	CCAGAACACAACAGAGTTAGCGTTGCATTTTCTACATTCAATGTGGGCT
Core28extB	TGAAATTGCTGACCTCTTAAACGGACCTAAAATACATTCAATGTGGGCT
Core29extB	TTCGTAATGGGCTTAAGACTAAAGAAAACACTTACATTCAATGTGGGCT
Core30extB	CACCAGTCCGCTCATGCTCAGAGCACAAACAATACATTCAATGTGGGCT
Core31extB	ATTCTGGCATATTACCGAACCACCGAGGCAGGTACATTCAATGTGGGCT
Core32extB	TCCGAACTTTATCCGCTGCAGGGACCGATATATACATTCAATGTGGGCT
Core33extB	AGTAAACACATGGTCACGTCACCCGACAATGATACATTCAATGTGGGCT
Core34extB	TGGCTATTAAACAGAGAAGGCCGGATTAAAGGTACATTCAATGTGGGCT
Core35extB	AAGAATACGCCTGCAACAAAATCAGAGCCATTTACATTCAATGTGGGCT
Core36extB	CGCATTTCCCAAAATACGAAATCCGGCGCAGATACATTCAATGTGGGCT
Core37extB	GGCACGAACTCGCCCTGAGATTTGCTGACCAATACATTCAATGTGGGCT
Core38extB	AGAAGATAAGTCTTTAAGTTTGCCCCAGAGCCTACATTCAATGTGGGCT
Core39extB	TTAACACCGTGGCACATTTCATCGTGCCATCTTACATTCAATGTGGGCT
Core40extB	ATGTCCCGACATAAATAGGCACCAGTAAAATATACATTCAATGTGGGCT
Core41extB	TATTTACGTATAGGGGAATACACTACTTTTTCTACATTCAATGTGGGCT
Core42extB	CCTCAAATATCTTTAGCCAGCGCCTAAAGGTGTACATTCAATGTGGGCT
Core43extB	AATGAAAAGAGCCGTCGTCACAATGACACCACTACATTCAATGTGGGCT
Core44extB	CTGAATTGACGCCAGGAACCGGATTCATTCAGTACATTCAATGTGGGCT
Core45extB	ATTCATGCAAACGACGGCTGACCTAAACACCATACATTCAATGTGGGCT
Core46extB	TCTAAAATATCAAACCAATTATTCAAACGTCATACATTCAATGTGGGCT
Core47extB	ATAGATTAATCTAAAGACCGACTTCCAGTAGCTACATTCAATGTGGGCT
Core48extB	GTTGGGTATCAACCTTCGGAACGAGCGACCTGTACATTCAATGTGGGCT
Core49extB	ACGTTGTAGCACGACTGAACCGAATATCATCGTACATTCAATGTGGGCT
Core50extB	TCGTATTAGAAGGAGCATACCCAATCTTACCGTACATTCAATGTGGGCT
Core51extB	AAGTATTATATCAGATAAGGAAACCAGATAGCTACATTCAATGTGGGCT
Core52extB	GGATGTGCGCCAGCTTTGGCTCATACGTTAATTACATTCAATGTGGGCT
Core53extB	CTCTTCGCAAACCAGGATTGTGAATATTACAGTACATTCAATGTGGGCT
Core54extB	AACCACCAAATCCTTTTACATACAAAAGACAATACATTCAATGTGGGCT
Core55extB	TTCCTGATGACTTTACAACGCAAACAATAGAATACATTCAATGTGGGCT
Core56extB	GCACTCCATGCAAGGCAAAGCTGCATTCATTATACATTCAATGTGGGCT
Core57extB	GGTGCCGGTATTACGCCTGACGAGTCATCAAGTACATTCAATGTGGGCT
Core58extB	GGGTTAGAATGAATATCAGAGAGAAATAACATTACATTCAATGTGGGCT
Core59extB	ATTGTTTGGGGAGAAAACAAAGTCGGGAGAATTACATTCAATGTGGGCT
Core60extB	CAGTATCGTGTGAGCGGCATAGTAACGACGATTACATTCAATGTGGGCT
Core61extB	CCGTGCATGTGGGAACGCAGATACTTTGCAAATACATTCAATGTGGGCT
Core62extB	AACGTCAGACCTACCAAATAGCTAAAGAACTGTACATTCAATGTGGGCT
Core63extB	TTTACATCGATTATACAAAGTAAGCGAGGAAATACATTCAATGTGGGCT
Core64extB	ACATTAAAGCCTCAGGAAAAATCTTATACCAGTACATTCAATGTGGGCT
Core65extB	GATTCTCCCTGCCAGTAACAACATTTACCTTATACATTCAATGTGGGCT
Core66extB	AATTATTCCATTTGAATTTATCCCCTACAATTTACATTCAATGTGGGCT
Core67extB	GAATACCAACATAAATTTTGCCAGAACGAGCGTACATTCAATGTGGGCT
Core68extB	TTCCTGTAGAAGATTGATTGAATCAGAATGACTACATTCAATGTGGGCT
Core69extB	GAACGCCAAACGTTAAGTCCAATAACCCTGACTACATTCAATGTGGGCT
Core70extB	AACAATTTATTTCAATACAGAGAGTAACCCACTACATTCAATGTGGGCT
Core71extB	GAAACAGTAGTTACAACATTAGACAGAGGGTATACATTCAATGTGGGCT
Core72extB	AAAAACAGGCCAGCTTTTTACCAGAGAGCAACTACATTCAATGTGGGCT

Core73extB	AAATTGTATCAAAAATGAGAGGCTATAACGCCTACATTCAATGTGGGCT
Core74	ATTGTTTGGGGAGAAAACAAAGTCGGGAGAAT
Core75	CAGTATCGTGTGAGCGGCATAGTAACGACGAT
Core76	CCGTGCATGTGGGAACGCAGATACTTTGCAAA
Core77	AACGTCAGACCTACCAAATAGCTAAAGAACTG
Core78	TTTACATCGATTATACAAAGTAAGCGAGGAAA
Core79	ACATTAAAGCCTCAGGAAAAATCTTATACCAG
Core80	GATTCTCCCTGCCAGTAACAACATTTACCTTA
Core81	AATTATTCCATTTGAATTTATCCCCTACAATT
Core82	GAATACCAACATAAATTTTGCCAGAACGAGCG
Core83	TTCCTGTAGAAGATTGATTGAATCAGAATGAC
Core84	GAACGCCAAACGTTAAGTCCAATAACCCTGAC
Core85	AACAATTTATTTCAATACAGAGAGTAACCCAC
Core86	GAAACAGTAGTTACAACATTAGACAGAGGGTA
Core87	AAAAACAGGCCAGCTTTTTACCAGAGAGCAAC
Core88	AAATTGTATCAAAAATGAGAGGCTATAACGCC
Core89	CCTTAGAACTGAGAGATTTTAGCGATTTTCAT
Core90	TTCTGTAAGGTTGGGTGGCTTATCATAGCAAG
Core91	GGTTGATAATGCCGGATCGAGCTTAGGTCAGG
Core92	GTAAAACTCTACAAAGGCCCGAAACCTTTTGA
Core93	TCATAGGTTCCTTGAAGCACCCAGAATCCAAA
Core94	CCGGCTTAATCGTCGCCCAACGCTTTACAAAA
Core95	ATAAATTAATCAGAAAAGAAAACGCCCCTCAA
Core96	TGAGAGATAGCATGTCAGGTCTTTCTGCGGAA
Core97	AAACTTTTACCGACCGTCTTTCCTTTATCAAC
Core98	CAAATCCAAGAATAAAGAGCATGTAAATAATA
Core99	TGATATTCTAAAAATTCTAAAGTATGATTCCC
Core100	AAGGCCGGATGCAATGTGCTGTAGGTTTGACC
Core101	TTTGAAATTCAAATATCCGTTTTTAACCTCCC
Core102	GTTAAATAATCGCAAGCGCGCCCACGGTATTC
Core103	CGCAAGGAAACCGTTCACTCCAACCAAAGCGA
Core104	TATTTTAAAGACAGTCTAATTGCTGACTTCAA
Core105	TCTTACCAAACATGTACCAGACGA
Core106	GCCTGTTTCCAGTAATAAAGTACC
Core107	GCGGGAGAACATCCAAGCGAGCTG
Core108	ACCAAAAAAATTAGCATAACCTGT
Core109	AAAGCTAAAATGTGTAGG
Core110	AGAGGCATTTTCGAGAGTATCATAACAAGAAAGAAACCA
Core111	AATAGTAGTAGCATTAAGCCTTTAATAACAGTCGGTGTCT
Core112	ACAGGCAAGGCAAAGCATTATGATAGATTTACTCAACAT
Core113	CGACAATAAACAACATTGCAGAACCCAACGCTTTTAATGG
Core114	GACAAAAGGTAAAGTAGTCCTGATGCGTTATAAGGC
Core115	AAAAGGTGGCATCAATCATTCCATTTTATTACTCATACAA
Core116	TTAGCTATATTTTCAGAACGAGCCCTGTACCTCATA
Core117extC	GACTTGCGGCTATTTTAACATAGCTTACATTTTATCGCCTATGGATCTT
Core118extC	TAAGAACAATCTTATATTAATTTTAATGTATCGCCTATGGATCTT
Core119extC	ACCAGACCAACAGTTCAGCTGAATGTGAGCCCTATCGCCTATGGATCTT
Core120extC	ATATCGCAAAAATCAATCATAAATATTTTATCGCCTATGGATCTT
Core121	CAAGAACGCAAGCAAGATTTTAGTTATCAAAA
Core122	ATCAATATCATTACACAAAGATTAACCT
Core123	GGAAGTTTGGAAGCAATAGGTAAATATATCTG
Core124	GTTTTAAGTACCTTAAATCACCTATTTT
Core125	TCATCGAGAAGGTATTAAGACCTAAACAACAGTA
Core126	CGTAGGAAATCGGCTGTGTGATAAATACAAAT
Core127extC	TAAGAAACCAGCCTTTTACCTGAGTCAGGTTTTATCGCCTATGGATCTT
Core128extC	TAAACAGGGAAGCGAATCGCGAGTACCTTATCGCCTATGGATCTT
Core129extC	ATGCTTTAAACCCTCGTCAGCCTGGATTCTCATATCGCCTATGGATCTT
Core130extC	TCGTCATAAATAGCAATTCGCCCCGTCGTATCGCCTATGGATCTT
Core131	TAAGAACAATCTTATATTAATTTTAATG
Core132	ACCAGACCAACAGTTCAGCTGAATGTGAGCCC
Core133extC	AAAAACAGCCATATTATTACCTTTTAATTTTCTATCGCCTATGGATCTT
Core134extC	TAACTGAAGAGCCTAACAATATATAACCTTGCTATCGCCTATGGATCTT
Core135extC	AAAAACCAAAATATTCTATAAGCATGTACCCCTATCGCCTATGGATCTT
Core136extC	AGAAGTTTTGGATAGCTATTTTGTCGGTAATCTATCGCCTATGGATCTT
Core137extC	AAGAATTGGAAATAGCTATCAAAAAACAAAGATATCGCCTATGGATCTT
Core138extC	ATTGAGCTTTAAGATTCTGAACATCATATATCGCCTATGGATCTT
Core139extC	ACTATCATTTGGGAAGAAGCAATATTCATATCTATCGCCTATGGATCTT
Core140extC	AAAAGGACTAACGGTTGAGGGCGCTTCTTATCGCCTATGGATCTT
Core141	ATGCTTTAAACCCTCGTCAGCCTGGATTCTCA
Core142	TCGTCATAAATAGCAATTCGCCCCGTCG
Core143extC	AAGCCCTTGCTAATATACAGTAACCAGAGGCGTATCGCCTATGGATCTT
Core144extC	CGAACAAACACCCTGACAATAACGATTGCTTTTATCGCCTATGGATCTT
Core145extC	AAAACGAAATTACGAGAGTAACAAGTCTGGCCTATCGCCTATGGATCTT
Core146extC	GTAGAAAGCAACTAATAAACGGCGACCAATAGTATCGCCTATGGATCTT
Core147extC	GCATGATTGTAGAAAAGCCCGAACGAAGGTTATATCGCCTATGGATCTT
Core148extC	CGCAATATAAAAGAAAACAATACAACTATATCGCCTATGGATCTT
Core149extC	TCAGGACGAACGTAACGATTTTGAATACATAATATCGCCTATGGATCTT
Core150extC	TGCGATTGCTTGCCCAGCTGGAGTCACGTATCGCCTATGGATCTT
Core151	AAGCCCTTGCTAATATACAGTAACCAGAGGCG
Core152	CGAACAAACACCCTGACAATAACGATTGCTTT
Core153extC	GCAACATAATAACGGAGGAATTATTAATGGAATATCGCCTATGGATCTT

Core154extC	GGAATAAGGTTACCAGGATGGCAATAATCCTGTATCGCCTATGGATCTT
Core155extC	TGAATAAGTTAAGAACTCCGGCACGACGACGATATCGCCTATGGATCTT
Core156extC	GAACGAGTCTTTAATCCAAAGCGCCATCGTAATATCGCCTATGGATCTT
Core157extC	AAGGGCGATTGACGGACTCAATCACCACCAGCTATCGCCTATGGATCTT
Core158extC	AATTCATCACCGTCCATCACCGTCAGTATATCGCCTATGGATCTT
Core159extC	CCCAAATCTACTTAGCATGAGAGCCGCTGACATATCGCCTATGGATCTT
Core160extC	AGTAATCCATAAGGTAAGTGTTCTAATCTATCGCCTATGGATCTT
Core161	GCAACATAATAACGGAGGAATTATTAATGGAA
Core162	GGAATAAGGTTACCAGGATGGCAATAATCCTG
Core163extC	TGAATTATATGGTTTAGAGCACTATCGACAACTATCGCCTATGGATCTT
Core164extC	TGGGAATTTTTATTTTAATAGATAGGATTTAGTATCGCCTATGGATCTT
Core165extC	CGGTCAATTTGACAAGGTTTTCCCCGAAAGGGTATCGCCTATGGATCTT
Core166extC	CTTTGAAAATAGGCTGGCCAGTGCGTGCGGGCTATCGCCTATGGATCTT
Core167extC	CCAATGAACAGAATCAATGCGCGAGGCAGATTTATCGCCTATGGATCTT
Core168extC	ACCATTAAGCGCGTGACAATAAAGGGACTATCGCCTATGGATCTT
Core169extC	CTCCATGTCACTACGACATGACCTCTTCTTTCTATCGCCTATGGATCTT
Core170extC	CCTGATAGCAAAAGCCTTGAATGTAATGTATCGCCTATGGATCTT
Core171	CGGTCAATTTGACAAGGTTTTCCCCGAAAGGG
Core172	CTTTGAAAATAGGCTGGCCAGTGCGTGCGGGC
Core173extC	CAGACTGTCCATTAGCGTGAGGCGTTGCTGAATATCGCCTATGGATCTT
Core174extC	GGTCATAGAGAGCCAGCAGTGCCACAGCAGCATATCGCCTATGGATCTT
Core175extC	CGAAAGAGAATTGTGTACCCCGCTCCTTAGTGTATCGCCTATGGATCTT
Core176extC	CATCTTTGGTACAACGGGAGTGACGGTTGTGATATCGCCTATGGATCTT
Core177	CAGACTGTCCATTAGCGTGAGGCGTTGCTGAA
Core178	GGTCATAGAGAGCCAGCAGTGCCACAGCAGCA
Core179	CGAAAGAGAATTGTGTACCCCGCTCCTTAGTG
Core180	CATCTTTGGTACAACGGGAGTGACGGTTGTGA
Core181	ACCACCGGCCGCCACCGAAATACCTTGTAGCA
Core182	TTTCATAGCCACCAGCCAGCCTCACTTG
Core183extC	TCAGAGCCATCAAAATAGTAATAATTTTTGAATATCGCCTATGGATCTT
Core184extC	CGCCGCCACCCCCTTAATAGAACCGAAAGCGTTATCGCCTATGGATCTT
Core185extC	CCGCTTTTAGTTTCCACTGGTTGGTCGGCTGATATCGCCTATGGATCTT
Core186extC	GAAAGACAGCTTTGAGGCTACGTGCGTCGGTGTATCGCCTATGGATCTT
Core187	CCGCTTTTAGTTTCCACTGGTTGGTCGGCTGA
Core188	GAAAGACAGCTTTGAGGCTACGTGCGTCGGTG
Core189	ATAAATCCGAATTTACATCAGTGAATGGTTGC
Core190	TCAGACGCTTTTGAGTACGCCTTCCTCG
Core191	TTCGGTCGACGTTGAAGGGGCCGAAGGAGAGA
Core192	CAACAACAGGAGCCCCAGCTGGGTGGTT
Core193	AAACAGCTTGATGCTTTCGCTGCCCGCAACAGCT
Core194	ATACATGGATTGGCCTTAATAACAATTGCAAC
Core195	TGGTAATAGCATTGACCAAACTATGGTAATAT
Core196	GCTCCAAACATCGCCCAGCCGGAATTCCTGTG
Core197	TATCAGCTTACCGATAGCCTAATGAGCTCGAA
Core198	CCCTGCCTCTATTATTCGCTTAATGCGCCGCT
Core199	AGTAACACTGAGACGTCACGCACGTGGC
Core200	ATTTTTTCATCTAAAGAAACGGGCAGGAGATC
Core201	AAAGGAATAAATGAAGCGGTCGGCAAAAT
Core202	TTAAGAGGGTGCCCGTAACGTGCTAGAATCCT
Core203	TAGGATTAAGTTTTAAAGCTAAACTTAAAGGG
Core204	GACGTTAGCAACTAAAGGCGCCAGCATTAATG
Core205	TTTGCTAAACAGTTTCAGACGGGCTTTCCAGT
Core206	TATAGCCCTATCACCGTGCCGTAAAGCACT
Core207	GGATAAGCCCTCAGATCACCC
Core208	GCGTAACGCCTCAGAGGTCTATCA
Core209	TGTAGCAGCAAGCCCGTGGAC
Core210	AGGTTTAGTACCGCCATGCCGTCGGCCGGCGATGCGCGTA
Core211	CAGGGATATTCCACAGCCGAAATCCACGCTGG
Core212	ACCCATGTACCGTAACGTCACCAGGAATAGCCCGAGATA

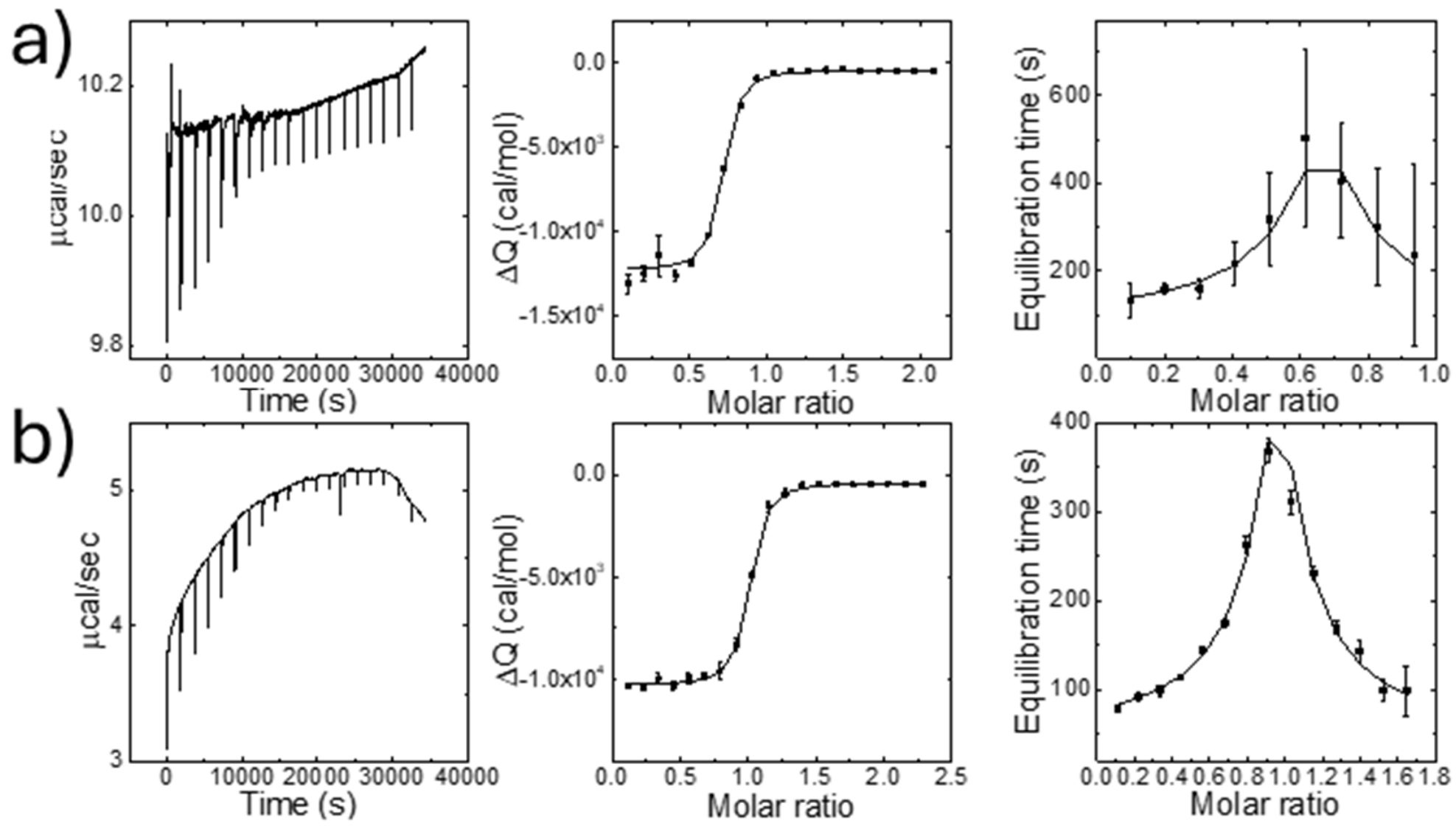

Figure S2. Representative ITC thermograms (left), integrated binding isotherms (center), and equilibration time curves (right) for the reversible covalent conjugation between ***1*** and ***8*** (a) or ***5*** (b).

Cell	Syringe	Kd (M)	ΔH ($cal{\cdot}mol^{-1}$)	TΔS ($cal{\cdot}mol^{-1}$)	k_{on} ($M^{-1}{\cdot}s^{-1}$)
8	***1***	3.71×10^{-7}	-1.18×10^{4}	-3.03×10^{3}	1.11×10^{3}
9	***1***	2.39×10^{-5}	-1.02×10^{4}	-3.91×10^{3}	0.60×10^{3}
5	***1***	3.54×10^{-7}	-9.84×10^{3}	-1.04×10^{3}	1.02×10^{3}
7	***1***	9.35×10^{-6}	-6.61×10^{3}	-2.47×10^{2}	0.37×10^{3}

Table S2. Thermodynamic parameters obtained by isothermal titration calorimetry (ITC) for the reversible covalent conjugation between ***1*** and hydroxylamine-functionalized DNA strand ***8*** or hydrazide-functionalized DNA strand ***9***, as well as small-molecule analogues (***5*** and ***7***, respectively). Reported values include dissociation constant (Kd), enthalpy change (ΔH), entropic contribution (TΔS) and forward rate constant (k_{on}).

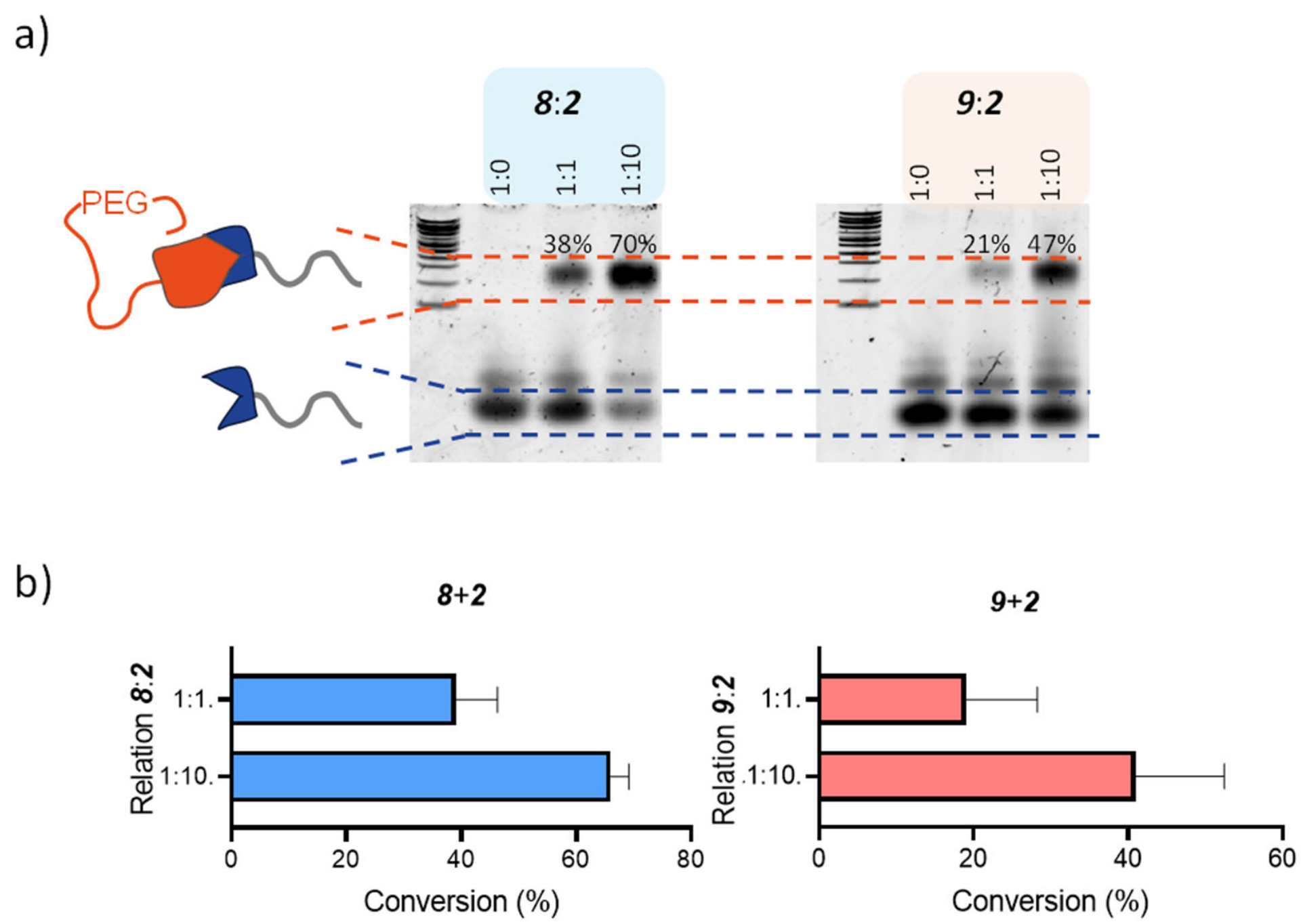

Figure S3. PAGE analysis of hydroxylamine-ssDNA ***8*** (blue) and hydrazide-ssDNA ***9*** (red) after reaction with PEG-FPBA ***2***. a) Representative PAGE gels of ***8*** or ***9*** incubated with ***2*** at different molar ratios: 1:0 (control with no PEG-FPBA), 1:1, and 1:10. Schematic representations are included to indicate the bands assigned to PEG-conjugated ssDNA and non-conjugated ssDNA. The percentage of functionalization is indicated above each lane. b) Quantification of conversion efficiency for ***8*** or ***9*** reacted with ***2*** across independent experiments (n = 4).

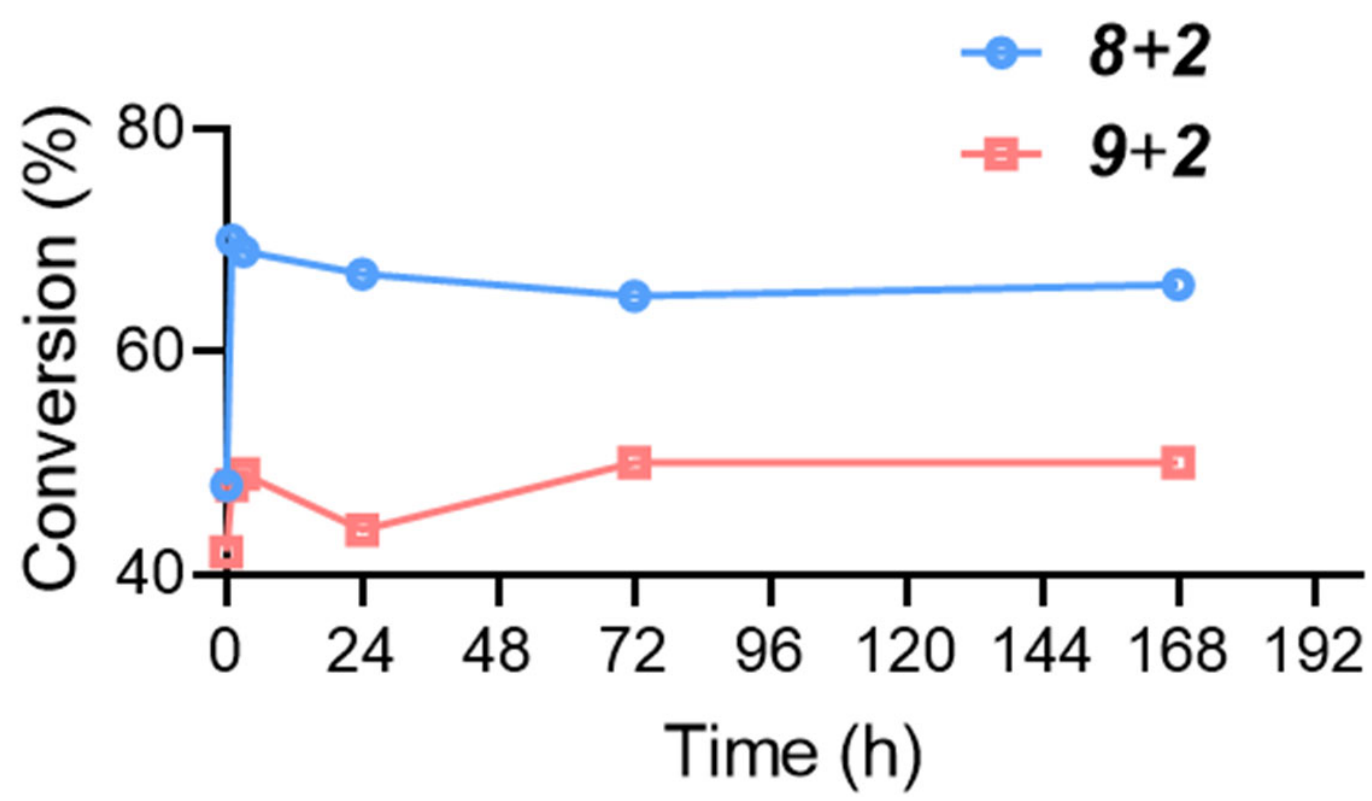

Figure S4. Quantification of conversion efficiency by PAGE for hydroxylamine-ssDNA ***8*** (blue) and hydrazide-ssDNA ***9*** (red) upon reaction with PEG-FPBA ***2*** for several days.

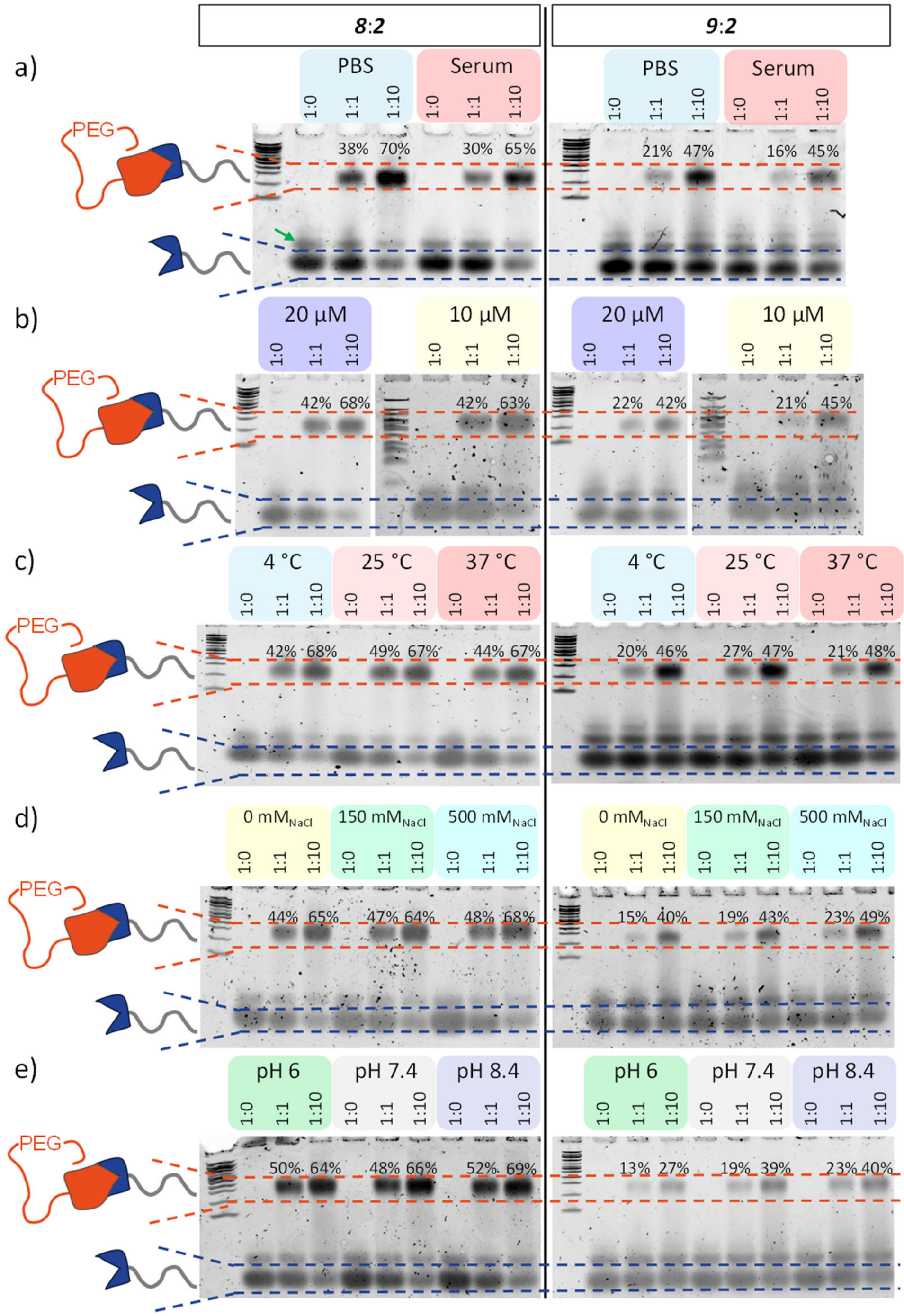

Figure S5. PAGE analysis of the conjugation of ***2*** to ssDNA ***8*** or ***9*** at different molar ratios (1:0, control without PEG-FPBA; 1:1; and 1:10) under various experimental conditions: (a) in PBS or DMEM supplemented with 10% FBS; (b) at two different ssDNA concentrations; (c) at different temperatures; (d) at different NaCl concentrations; and (e) at different pH values. Schematic representations are included to indicate the bands assigned to PEG-conjugated ssDNA and non-conjugated ssDNA. The green arrow in Figure S5a, shown as a representative case, indicates bands attributed to non-conjugated ssDNA dimers. The functionalization yield (%) is indicated above each lane.

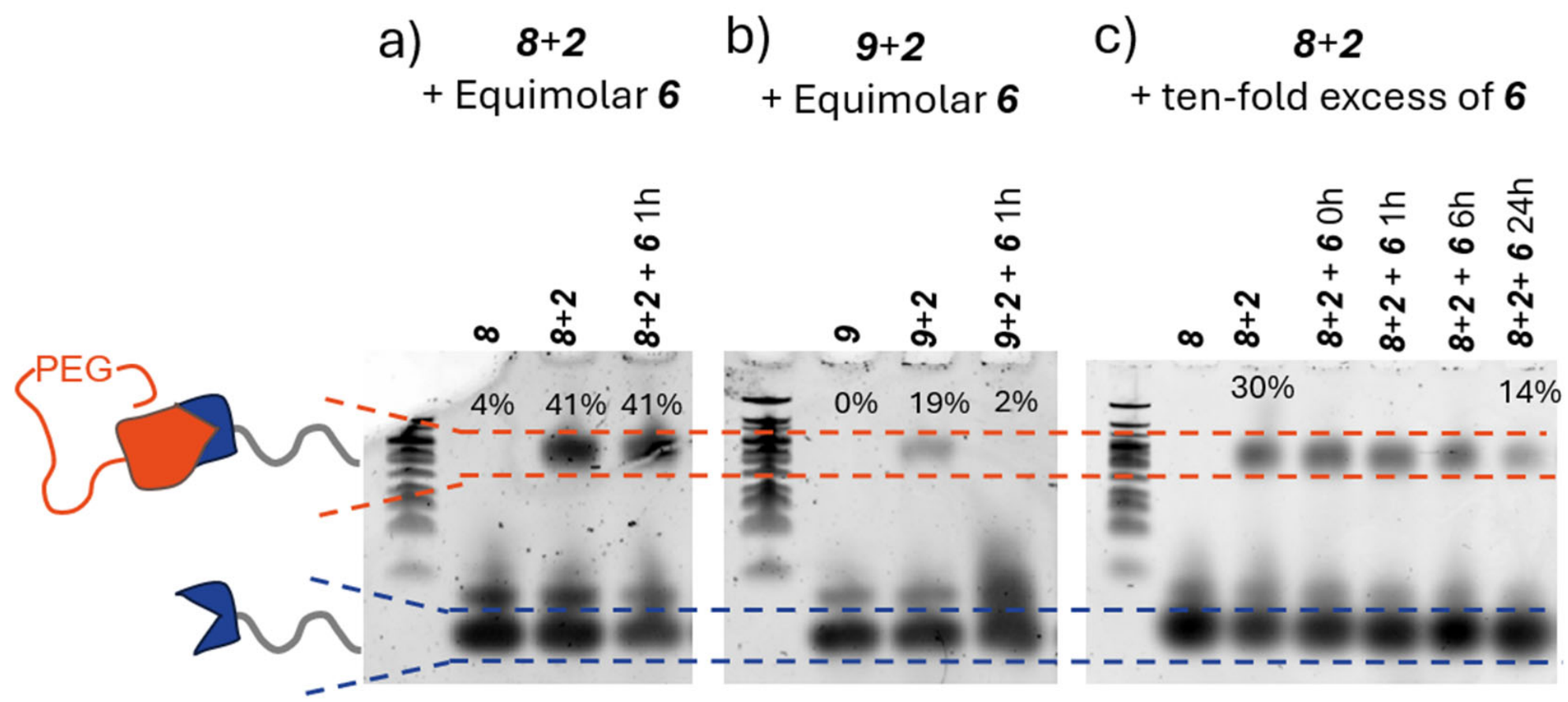

Figure S6. PAGE to assess dynamic conjugation of PEG-FPBA ***2*** to hydroxylamine-ssDNA ***8*** and hydrazide-ssDNA ***9*** upon addition of *O*-(carboxymethyl)hydroxylamine ***6***. a) PAGE showing ***8, 8+2*** and ***8+2*** upon reaction for 1h with ***6*** added at an equimolar ratio related to ***2***. b) PAGE showing ***9, 9+2*** and ***9+2*** upon reaction for 1h with ***6*** added at an equimolar ratio related to ***2***. c) PAGE showing ***8, 8+2*** and ***8+2*** upon reaction for up to 24h with ***6*** added at tenfold molar excess related to ***2***. Schematic representations are included to indicate the bands assigned to PEG-conjugated ssDNA and non-conjugated ssDNA. The percentage of functionalization is indicated above each lane.

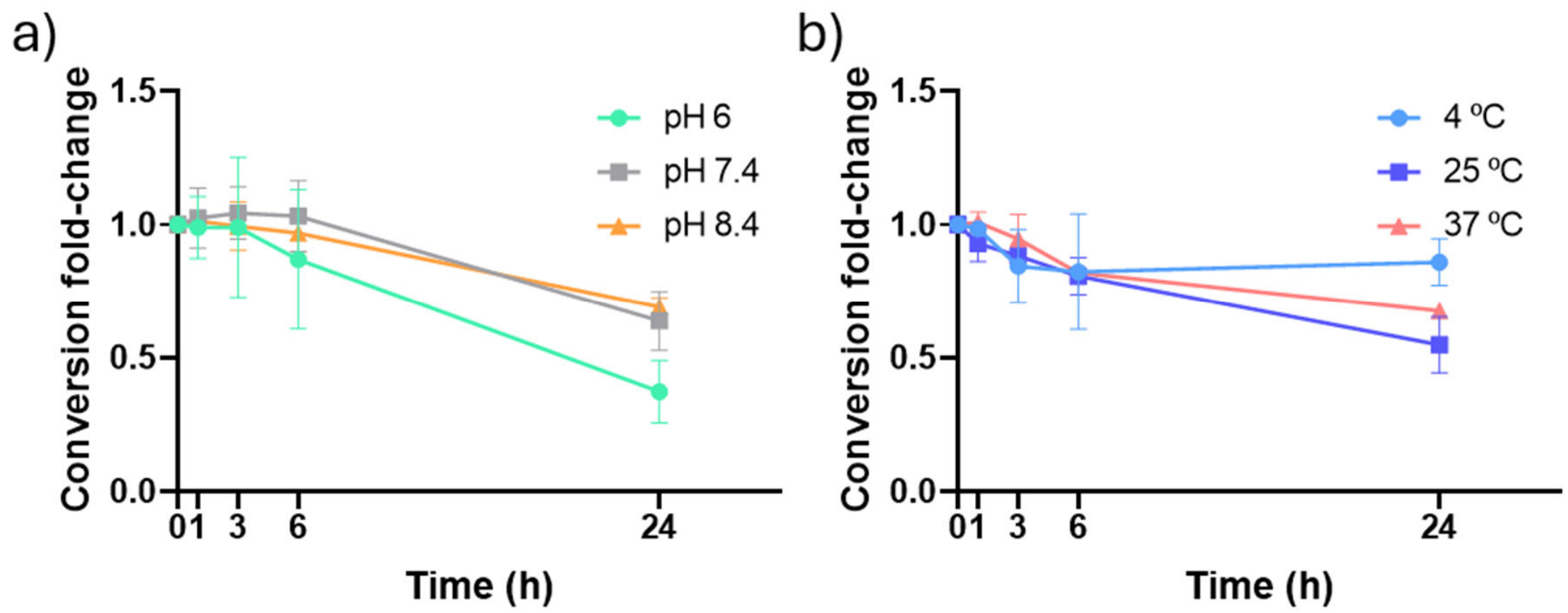

Figure S7. Fold reduction in the conversion of ***8***:***2*** after incubation with ***6*** under (a) different pH conditions or (b) at different temperatures over time. Values were normalized to the conversion measured for each sample at t = 0h. Data represent the mean and SD of n = 3 independent measurements.

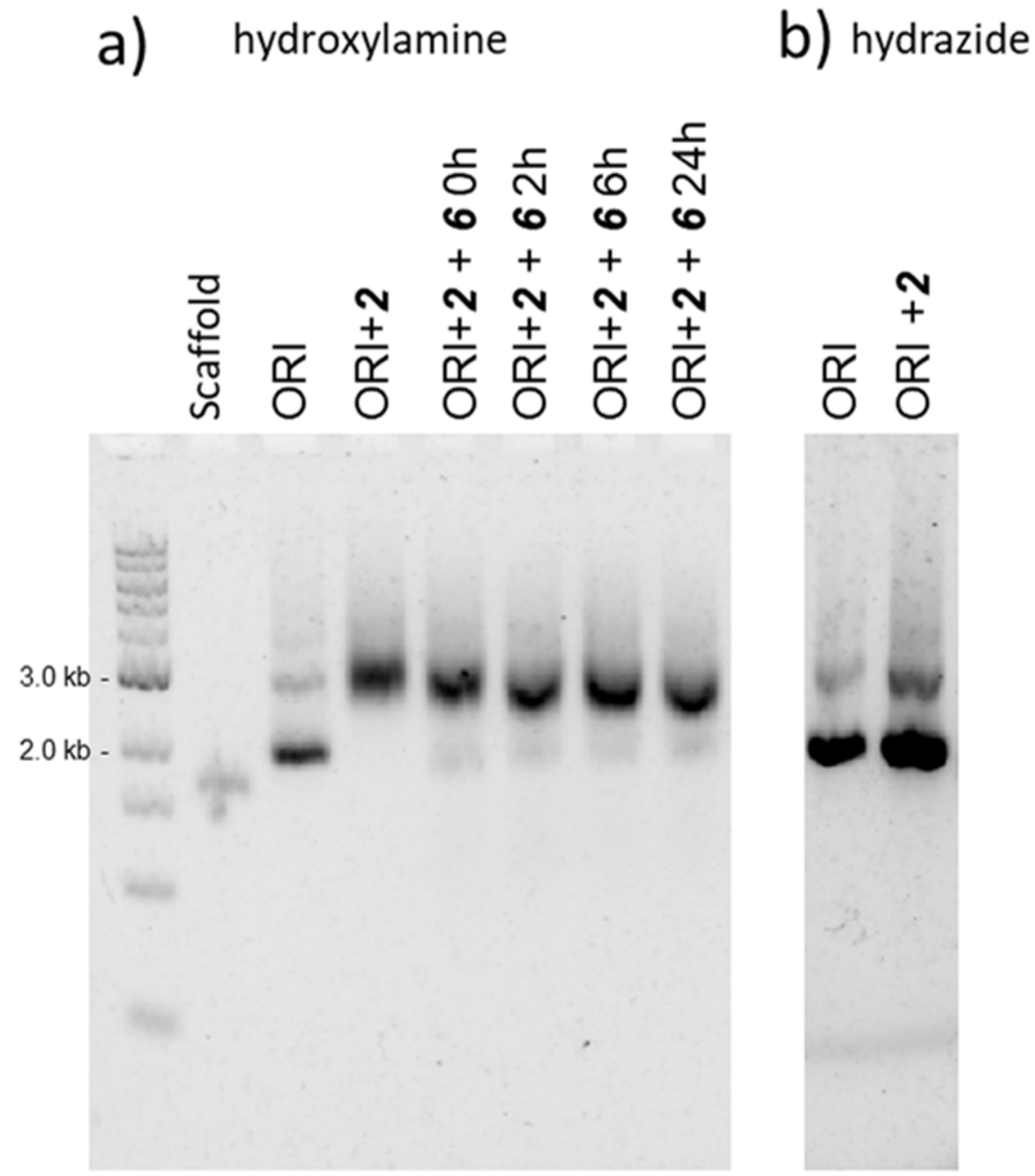

Figure S8. a) AGE showing conjugation of PEG-FPBA ***2*** to hydroxylamine-ORI (symmetric version of the DNA origami bearing ***8***) and its reversion upon addition of *O*-(carboxymethyl)hydroxylamine ***6*** reacted for up to 24h. A 1 kb DNA ladder was used as a marker, with selected band sizes indicated. b) AGE showing no conjugation of ***2*** with hydrazide-ORI (symmetric version of the DNA origami bearing ***9***).

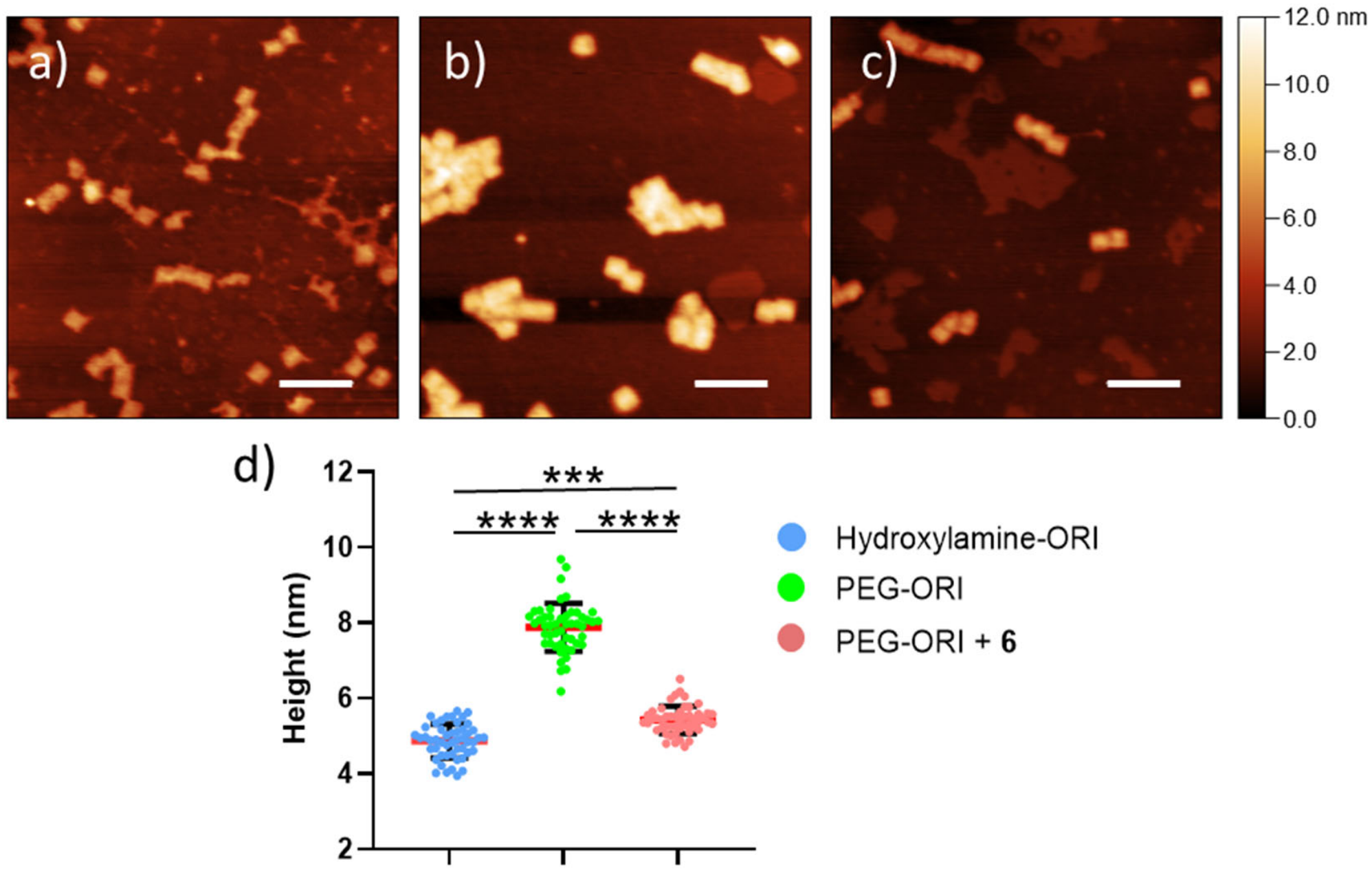

Figure S9. Representative Atomic Force Microscopy images for hydroxylamine ORI (a), PEG-ORI (b), and PEG-ORI incubated with ***6*** (c). Scale bar is 200 nm. d) Height distributions of the DNA origami structures determined by AFM. Individual values and mean height and standard deviation (SD) are indicated. At least 50 individual origami structures were analyzed for each sample. Statistical analysis was performed using one-way ANOVA in GraphPad Prism Significance thresholds were established at: $p \leq 0.05$ (*), $p \leq 0.01$ (**), $p \leq 0.001$ (***) and $p \leq 0.0001$ (****).

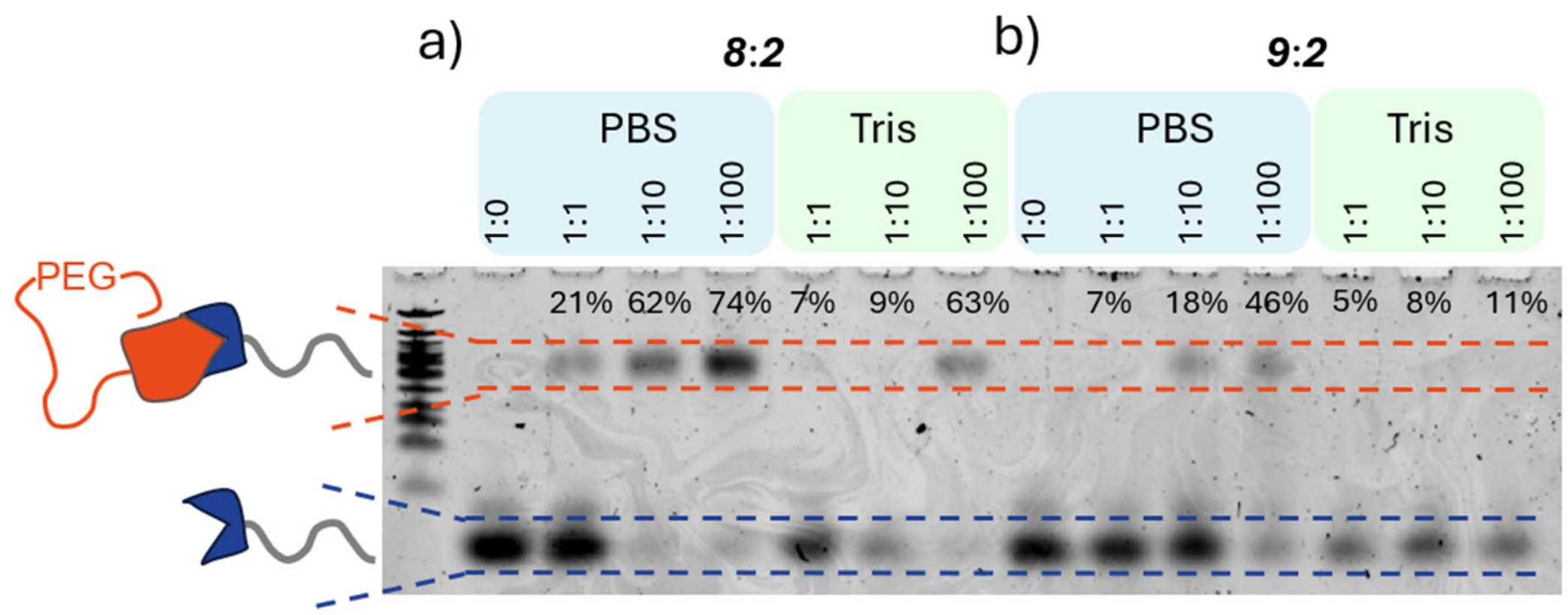

Figure S10. PAGE displaying (a) hydroxylamine-ssDNA ***8*** and (b) hydrazide-ssDNA ***9*** after reaction for 24h with PEG-FPBA ***2*** in either 1x PBS or 0.5 x Tris-EDTA buffer at different molar ratios: 1:0 (control without PEG-FPBA), 1:1, 1:10 and 1:100. Schematic representations are included to indicate the bands assigned to PEG-conjugated ssDNA and non-conjugated ssDNA. The percentage of functionalization is indicated above each lane. In this assay, the ssDNA concentration was set to 1 μM to match the concentration of hydroxylamine and hydrazide groups used in the DNA origami coupling assays.

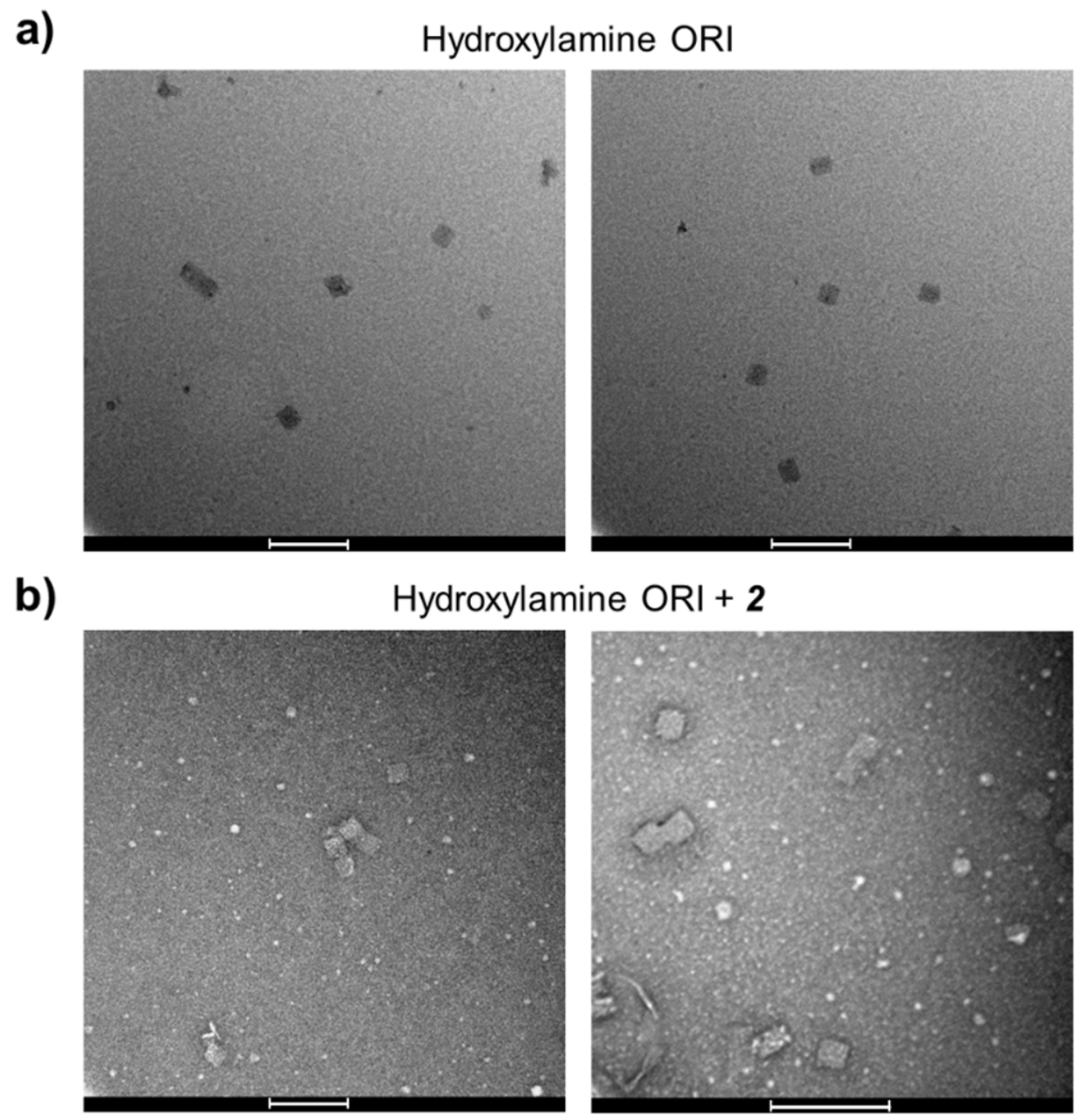

Figure S11. TEM images of DNA origami bearing ***8*** (symmetric design) stained with uranyl acetate a) without ***2*** and b) after conjugation with ***2***. Scale bar: 200 nm. Note that TEM was employed not to visualize PEG directly but rather to assess the structural integrity of the DNA origami before and after the modification with ***2***.

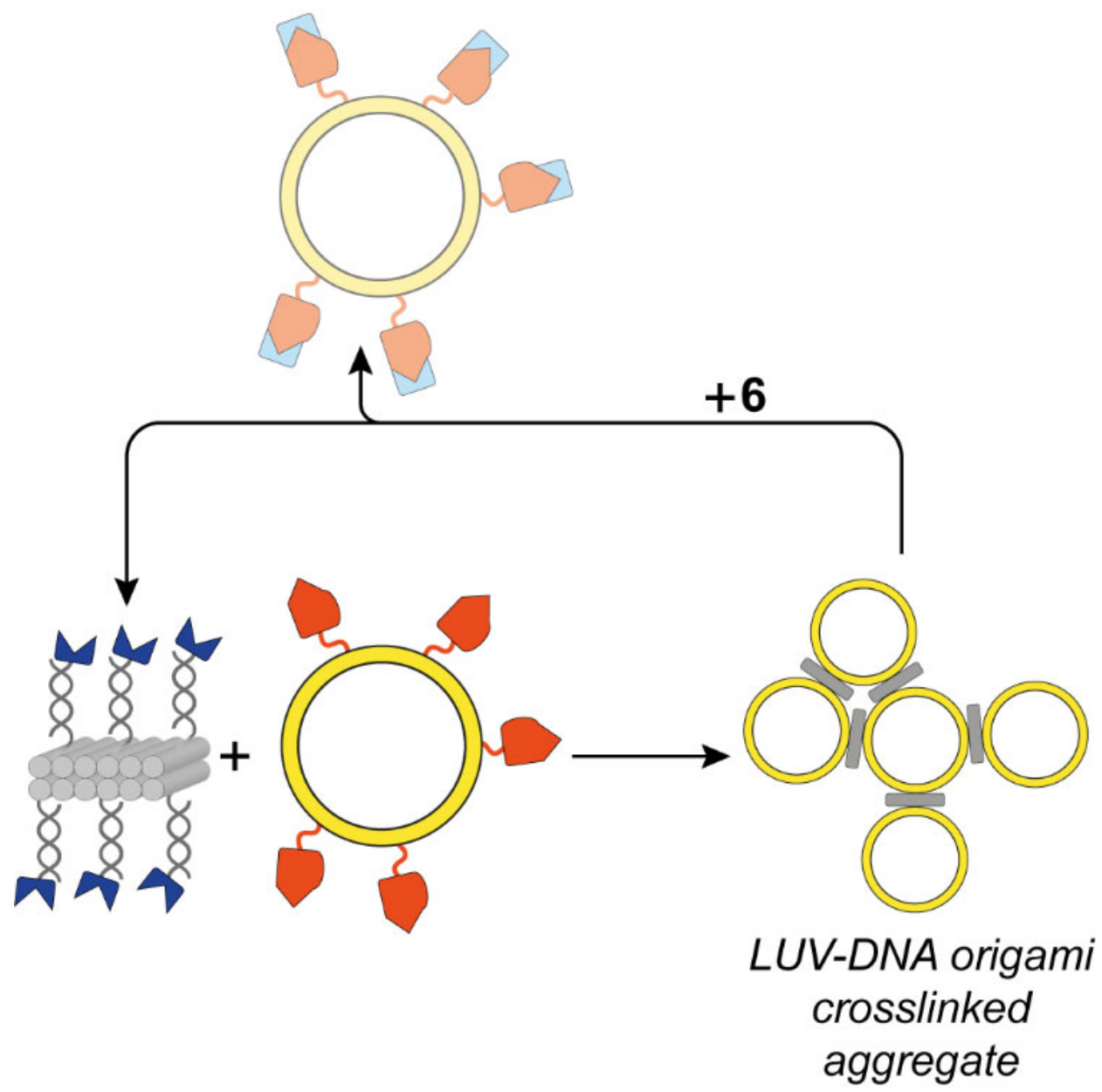

Figure S12. Schematic representation of LUV cross-linking mediated by the anchoring of DNA origami bearing ***8*** (symmetric design) to LUVs functionalized with hydrophobic FPBA derivatives (***3, 4***) and subsequent reversion by addition of *O*-(carboxymethyl)hydroxylamine ***6***.

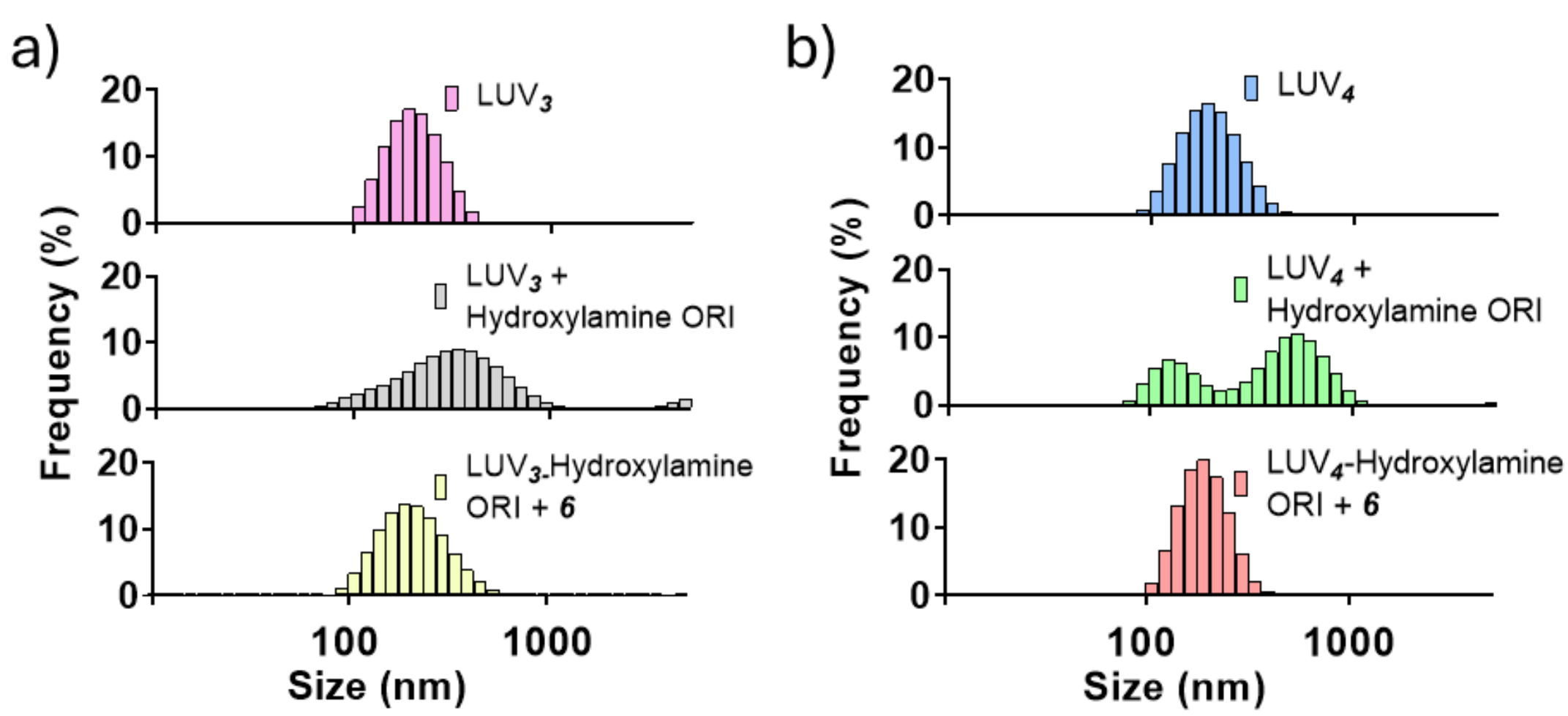

Figure S13. Size distribution histograms (in intensity) obtained by DLS for LUVs containing 1 % (w/w) of compound ***3*** (a) and ***4*** (b) upon interaction with DNA origami bearing **8** (symmetric design) and subsequent addition of ***6***.

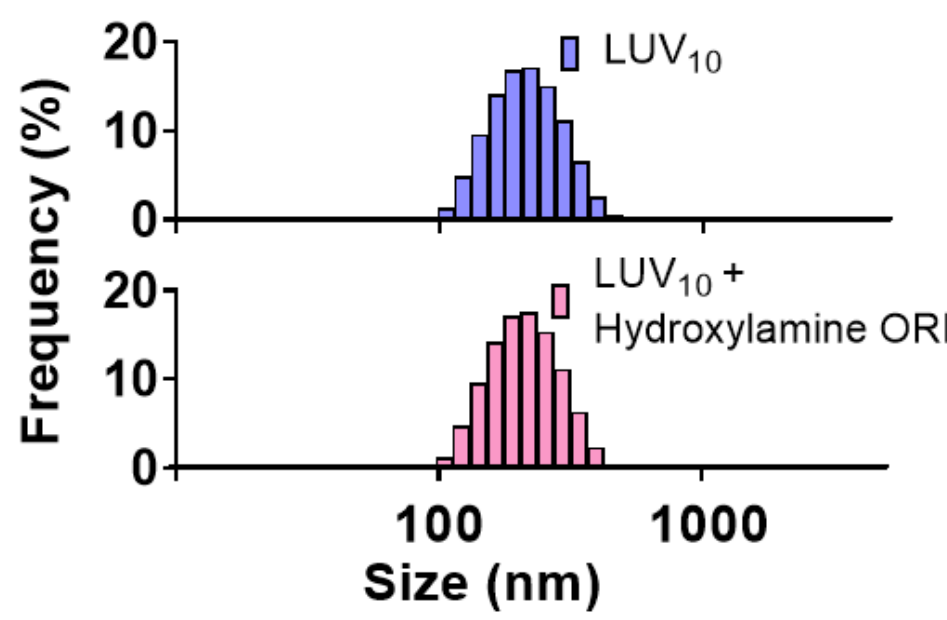

Figure S14. Size distribution histograms (in intensity) obtained by DLS for LUVs containing 1 % (w/w) of compound ***10*** upon interaction with DNA origami bearing **8** (symmetric design).

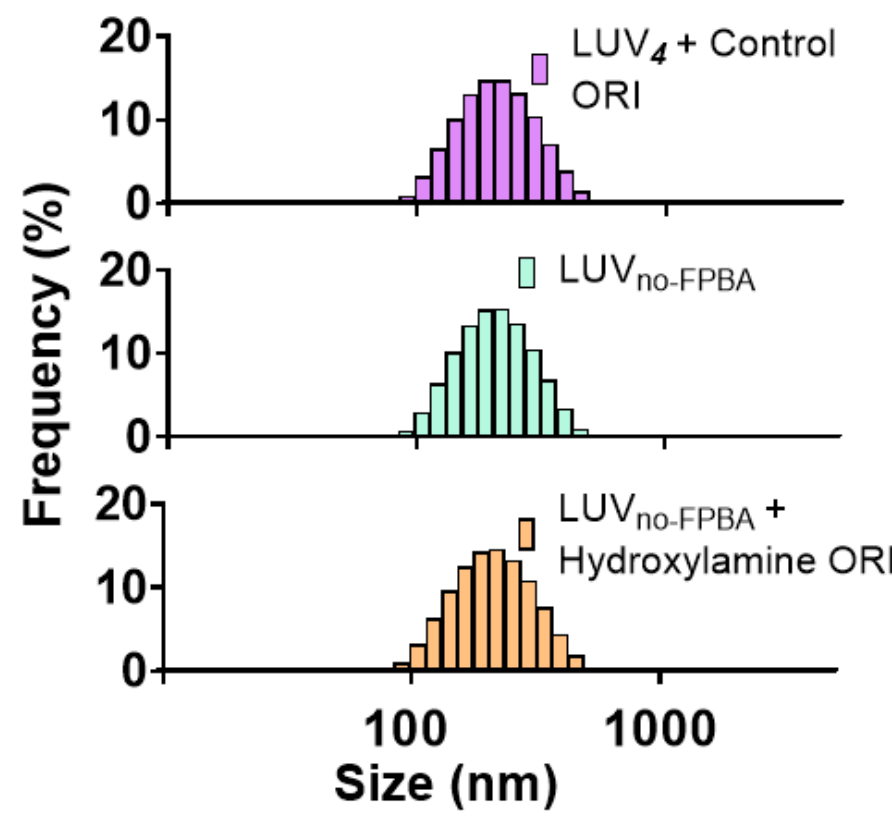

Figure S15. Control measurements of size distribution histograms (in intensity) obtained by DLS for (i) LUVs containing 1 % (w/w) of compound ***4*** upon interaction with DNA origami bearing no hydroxylamine group, (ii) LUVs with no FPBA derivative and (iii) LUVs with no FPBA derivative upon DNA origami bearing ***8*** (symmetric design).

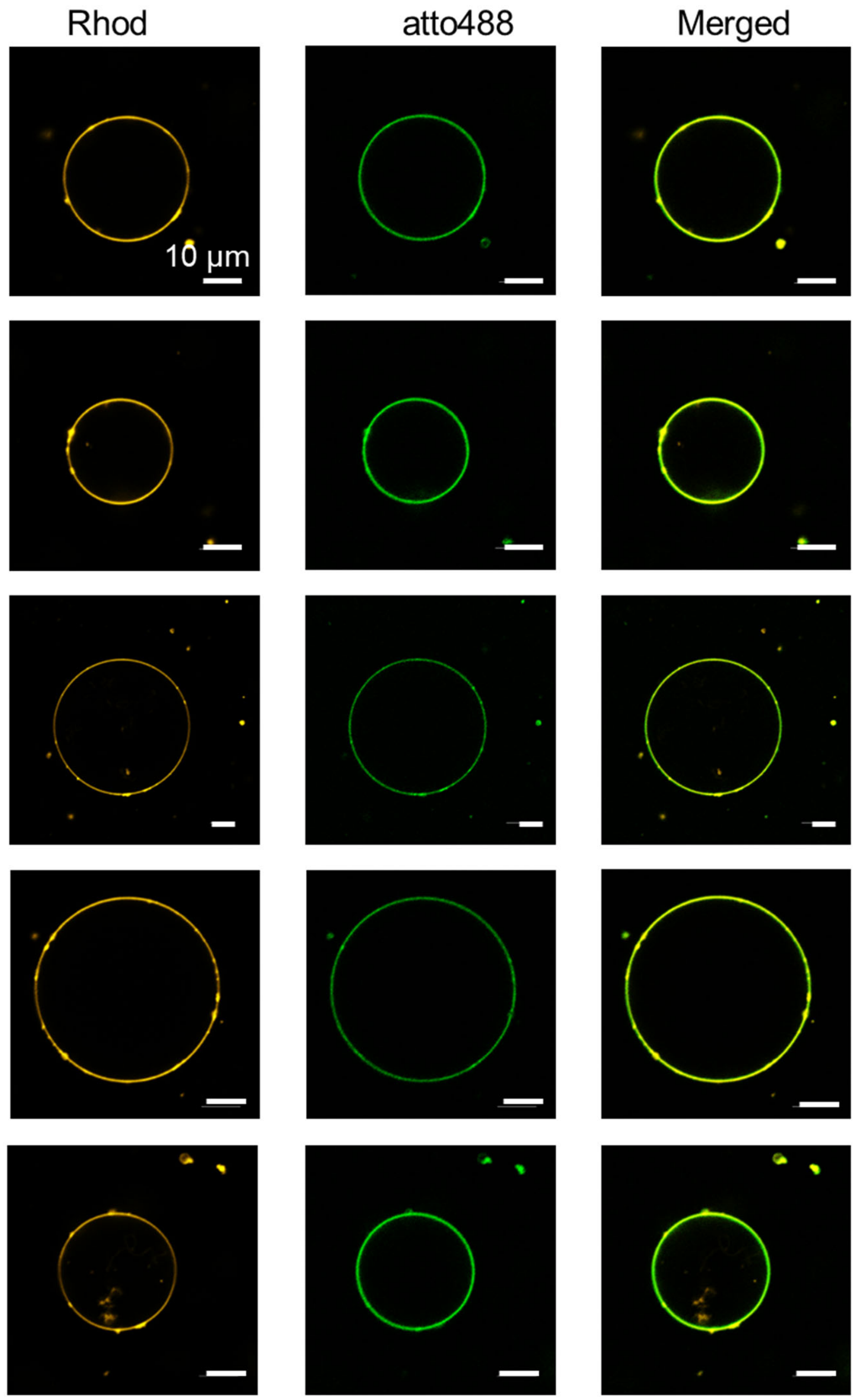

Figure S16. Complementary confocal images of GUVs with ***4*** incubated with Hydroxylamine ORI (atto488-labeled). Each channel (Rhodamine and atto488) is shown in separate columns. Scale bar corresponds to 10 µm.

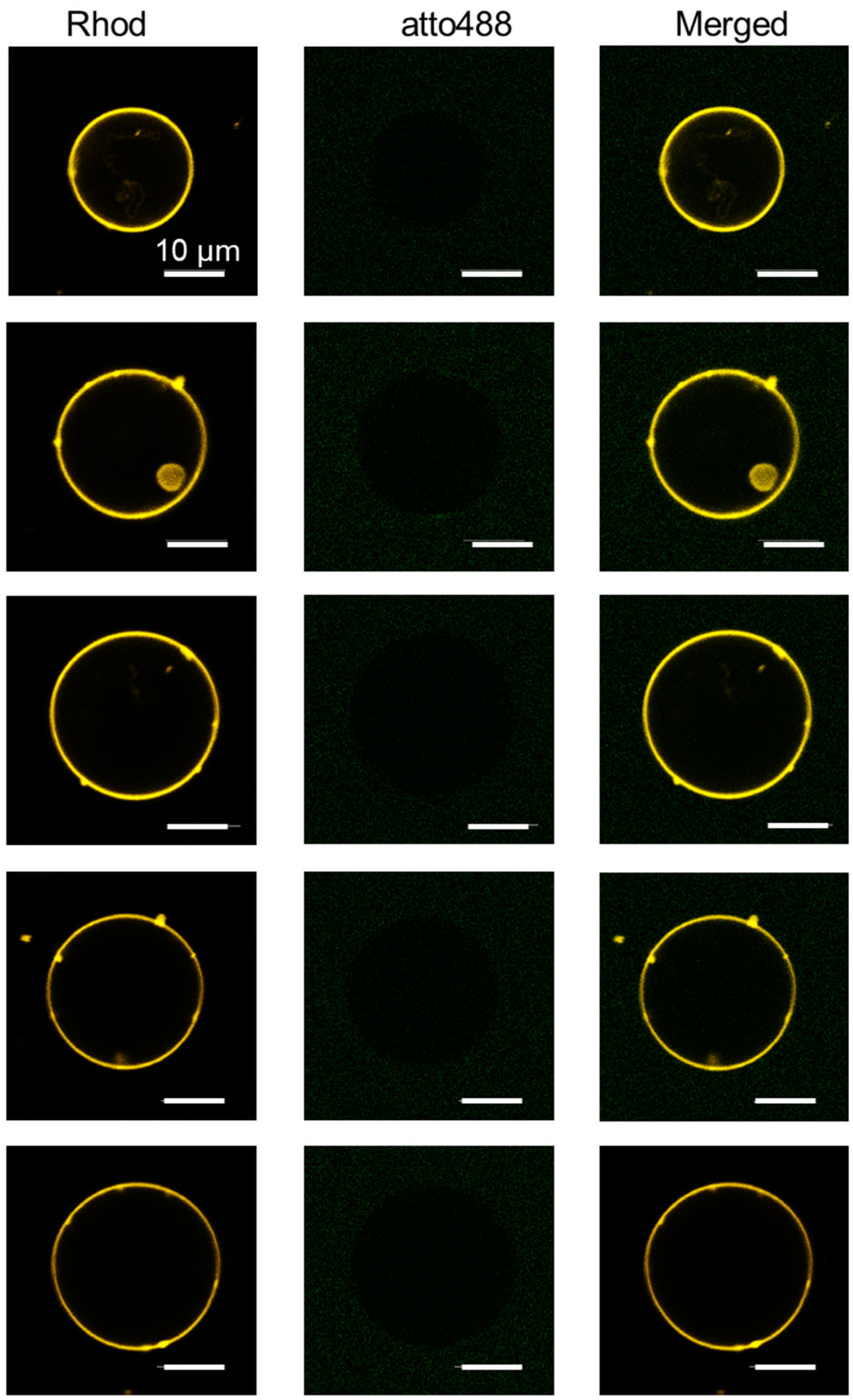

Figure S17. Confocal microscopy images of GUVs bearing 5% of ***4***, incubated with Hydroxylamine ORI (Atto488-labeled). The effect of the ***6*** competitor was evaluated by its addition post-conjugation. Each channel (Rhodamine and atto488) is shown in separate columns. Scale bar corresponds to 10 µm.

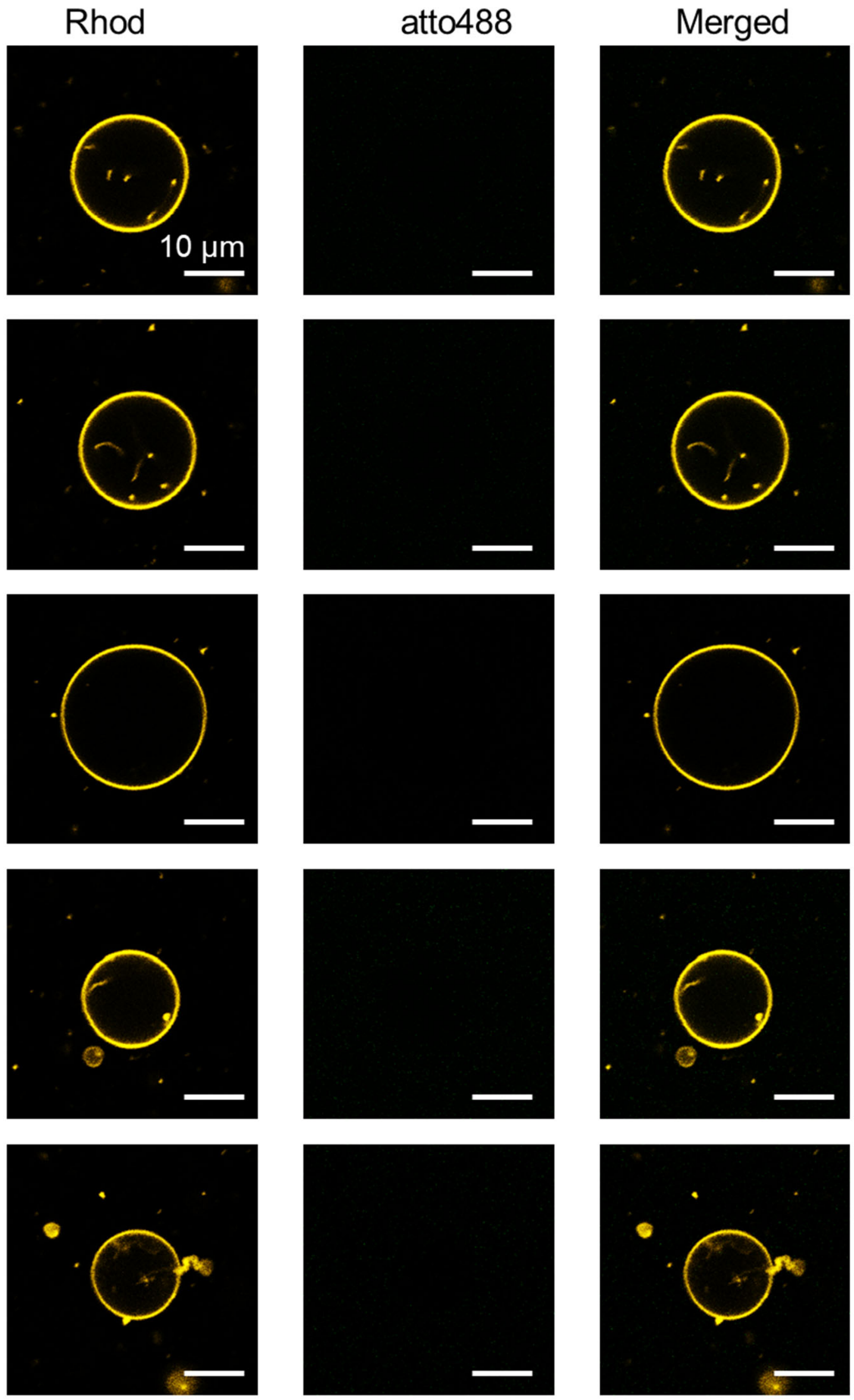

Figure S18. Control confocal microscopy images of GUVs without ***4***, incubated with ORI-oxy (Atto488-labeled). Each channel (Rhodamine and atto488) is shown in separate columns. Scale bar corresponds to 10 µm.

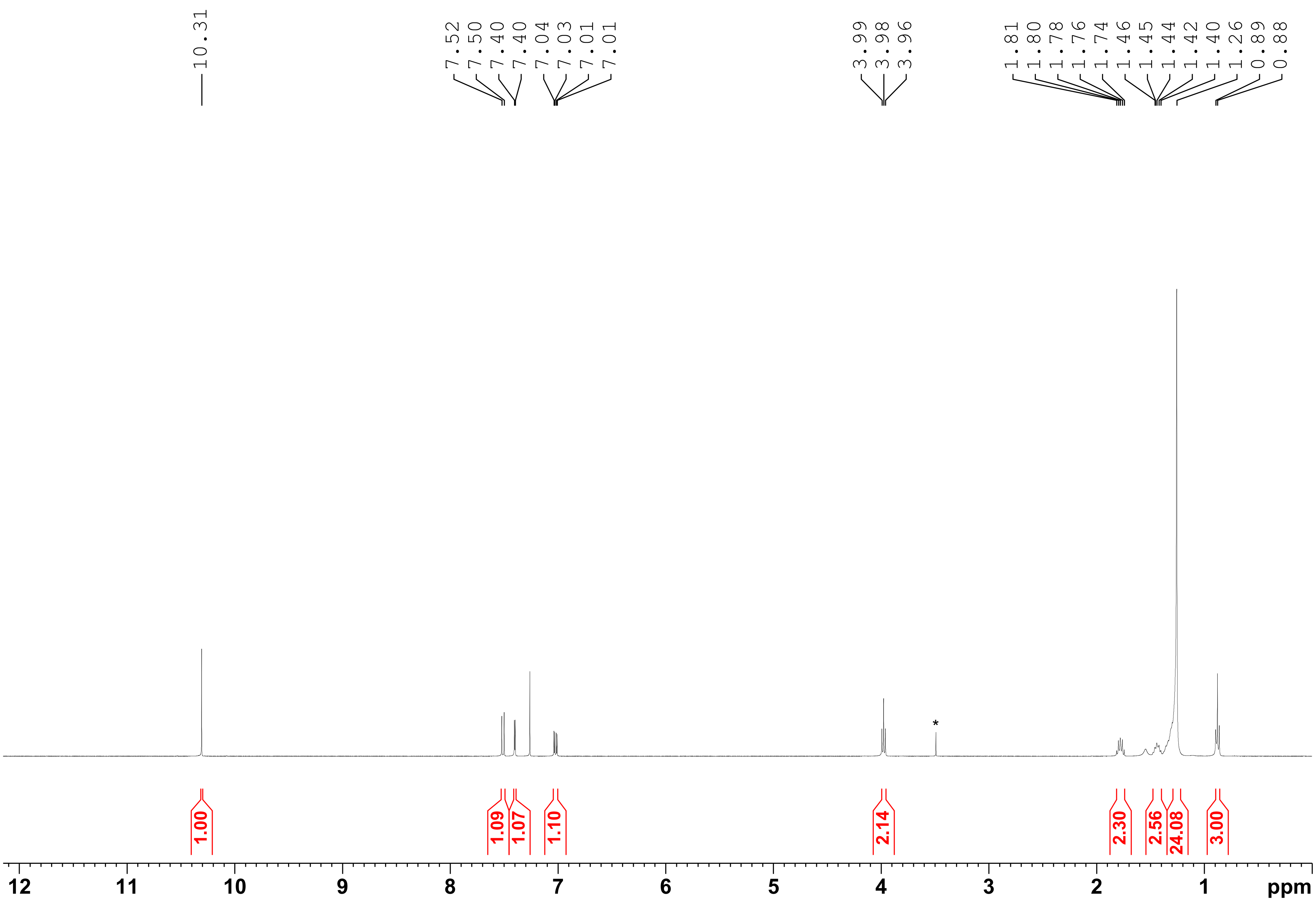

Figure S19. ^1H NMR spectrum of **6a** in $CDCl_3$. Minor residual solvent (MeOH) peaks are indicated by an asterisk (*).

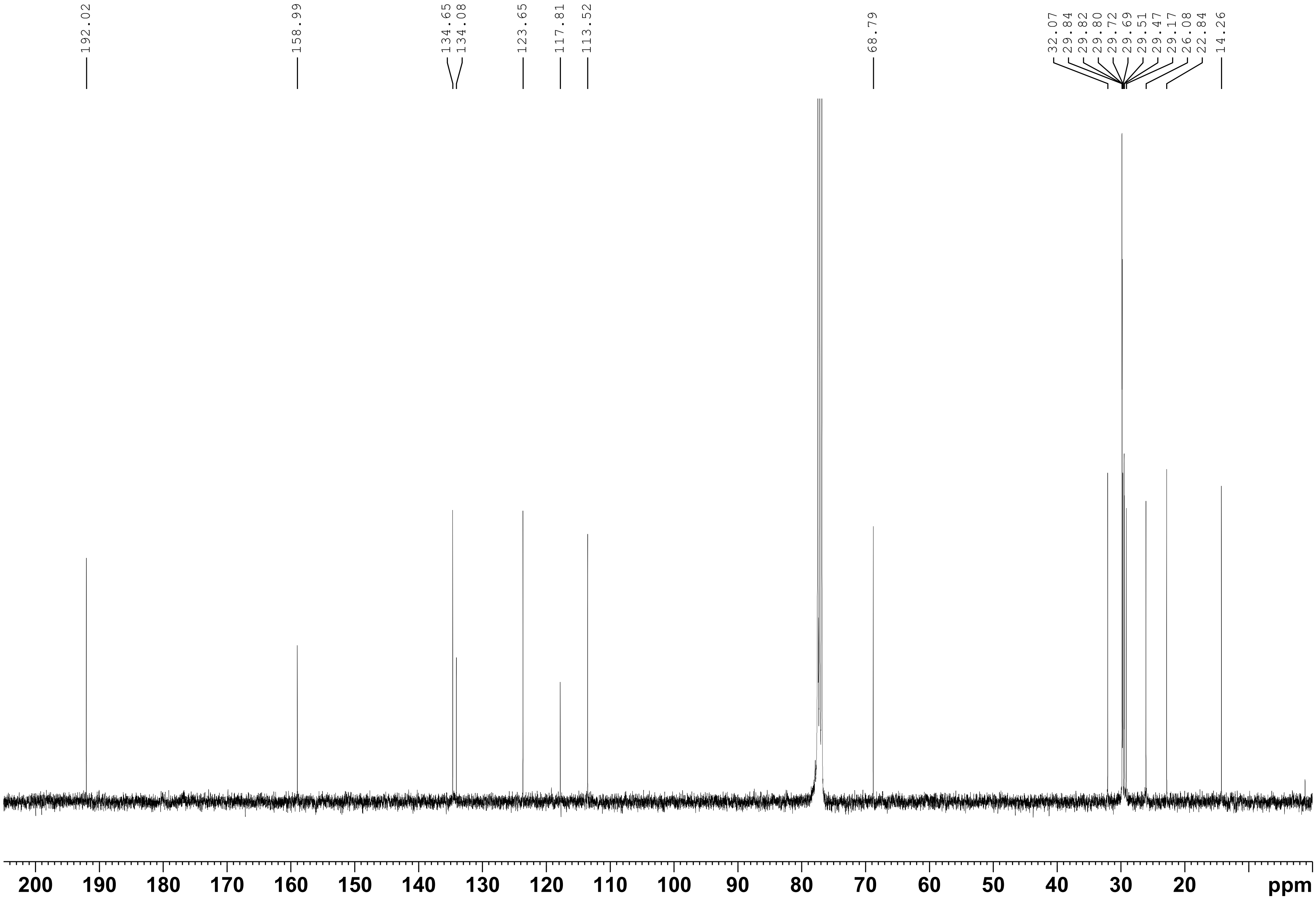

Figure S20. ^{13}C NMR spectrum of **6a** in $CDCl_3$.

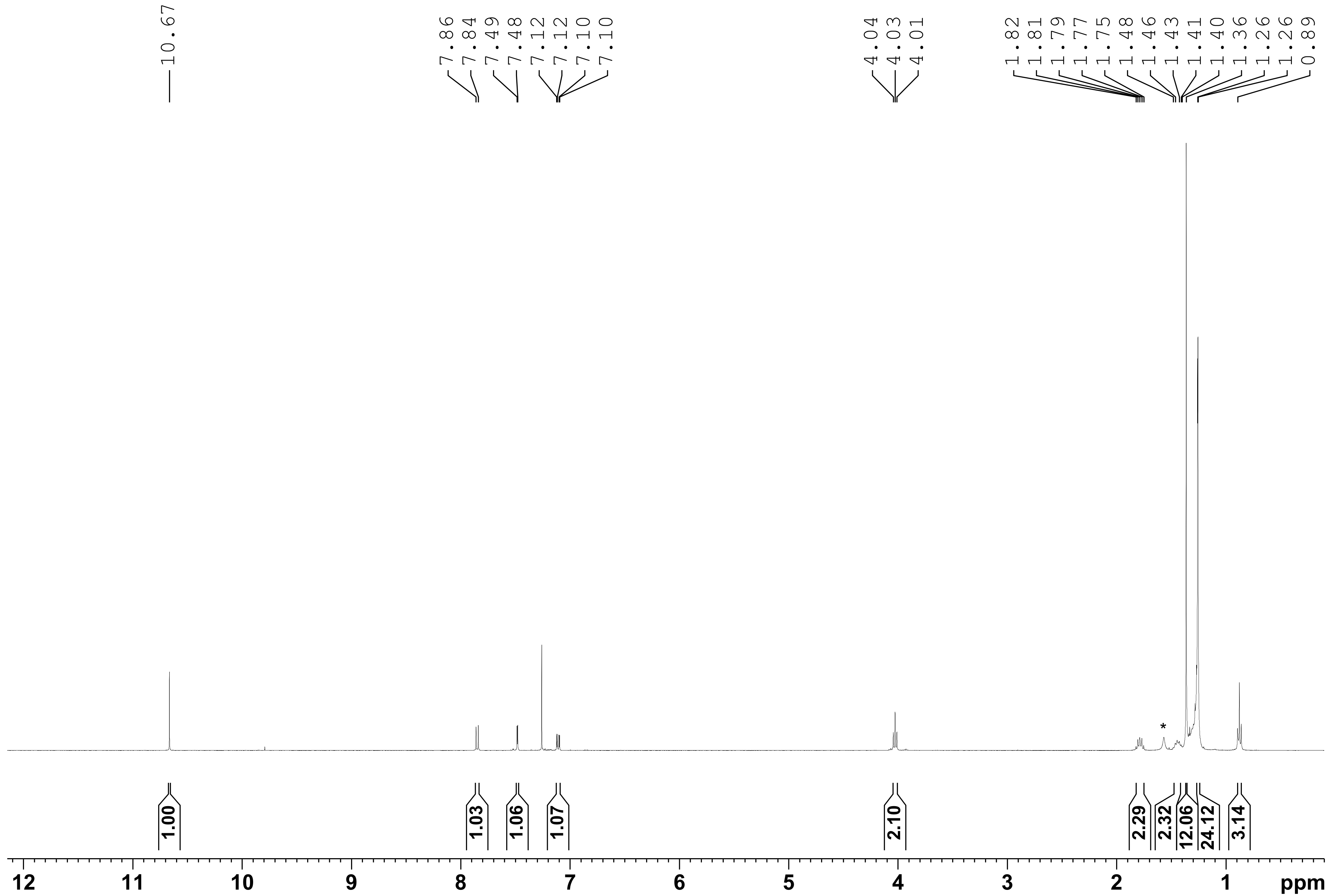

Figure S21. ^{1}H NMR spectrum of **7a** in $CDCl_3$. Minor residual solvent (H_2O) peaks are indicated by an asterisk (*).

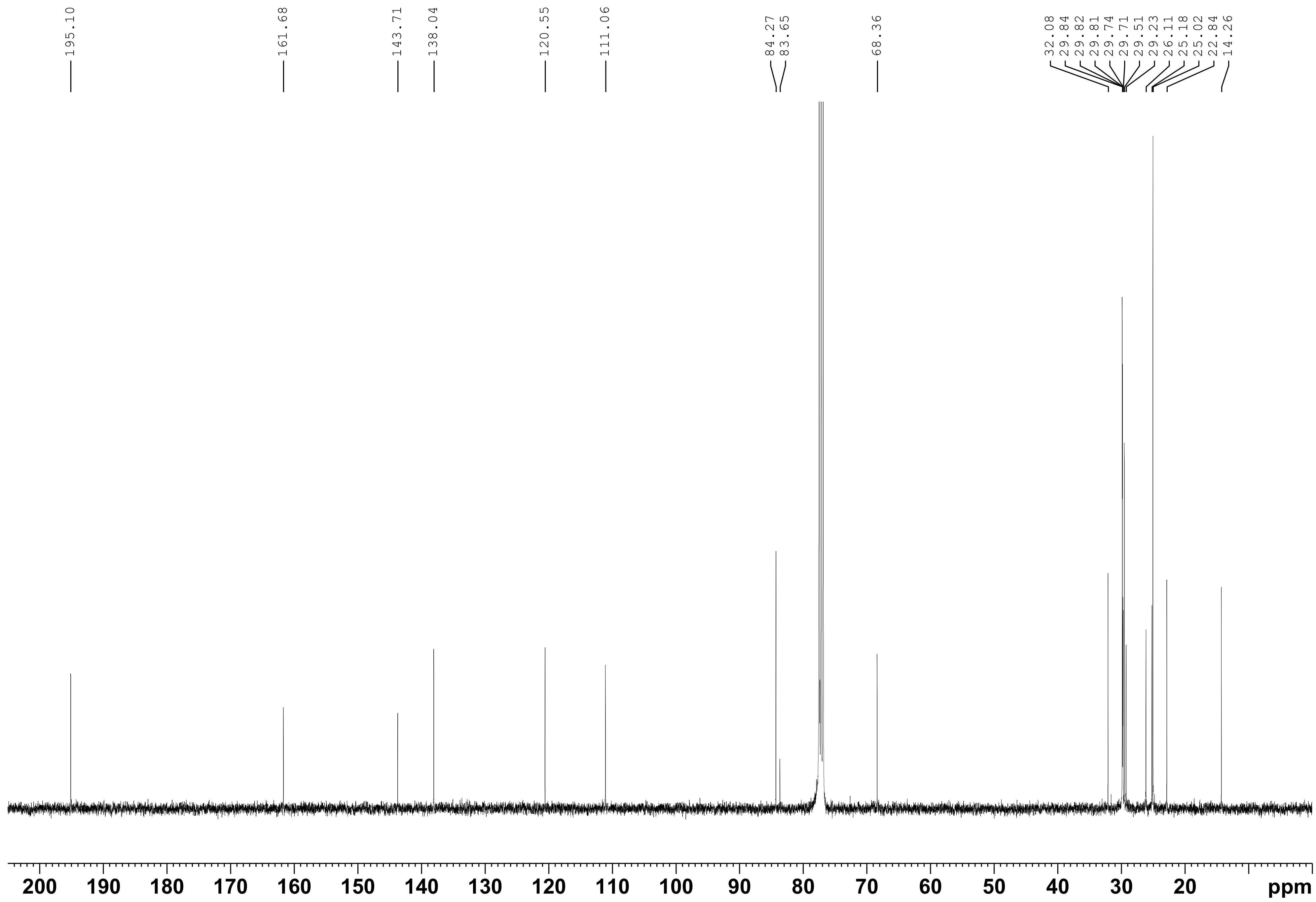

Figure S22. ^{13}C NMR spectrum of **7a** in $CDCl_3$.

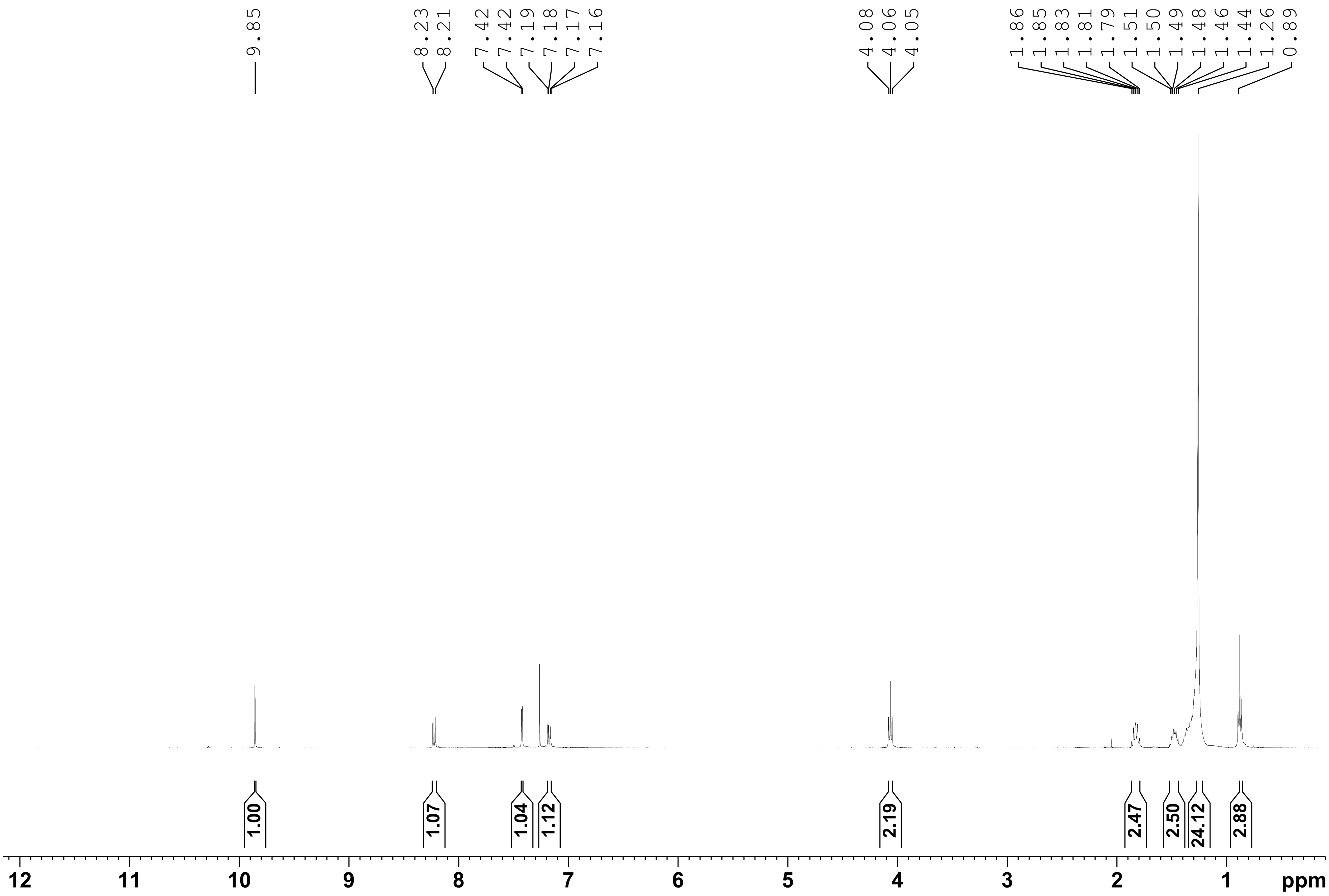

Figure S23. ^1H NMR spectrum of **3** in $CDCl_3$.

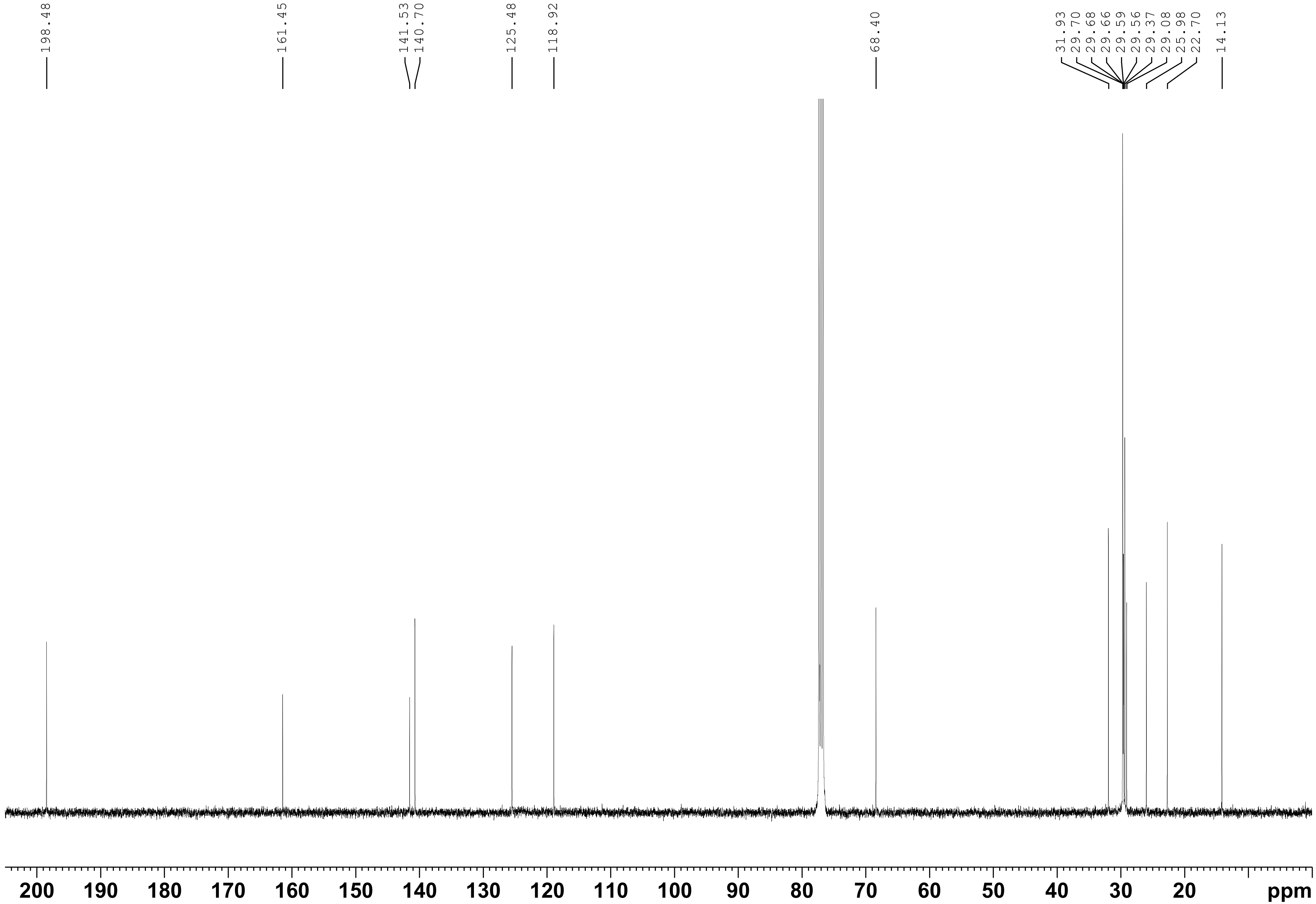

Figure S24. ^{13}C NMR spectrum of **3** in $CDCl_3$.

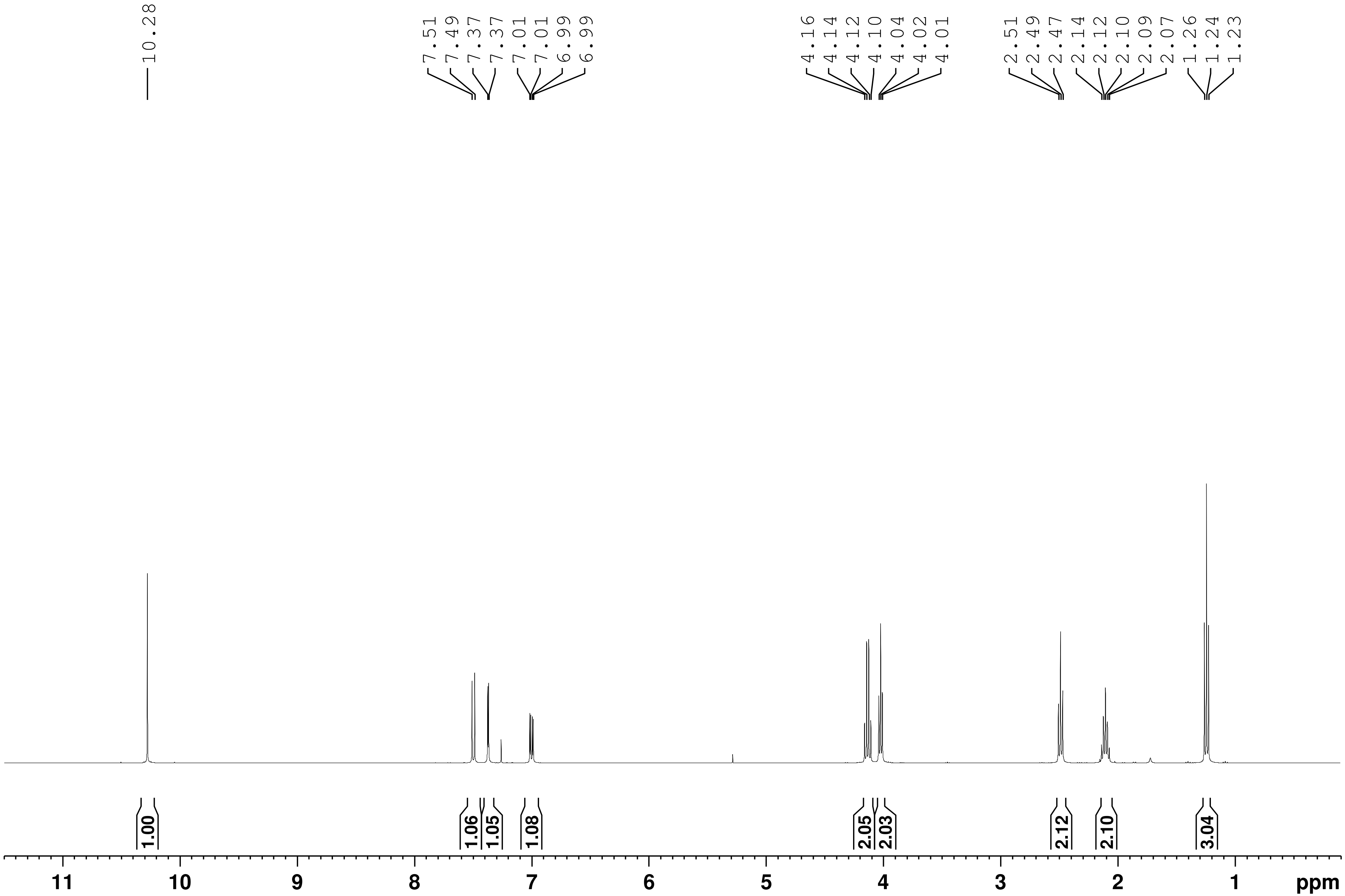

Figure S25. ^{1}H NMR spectrum of **1a** in $CDCl_3$.

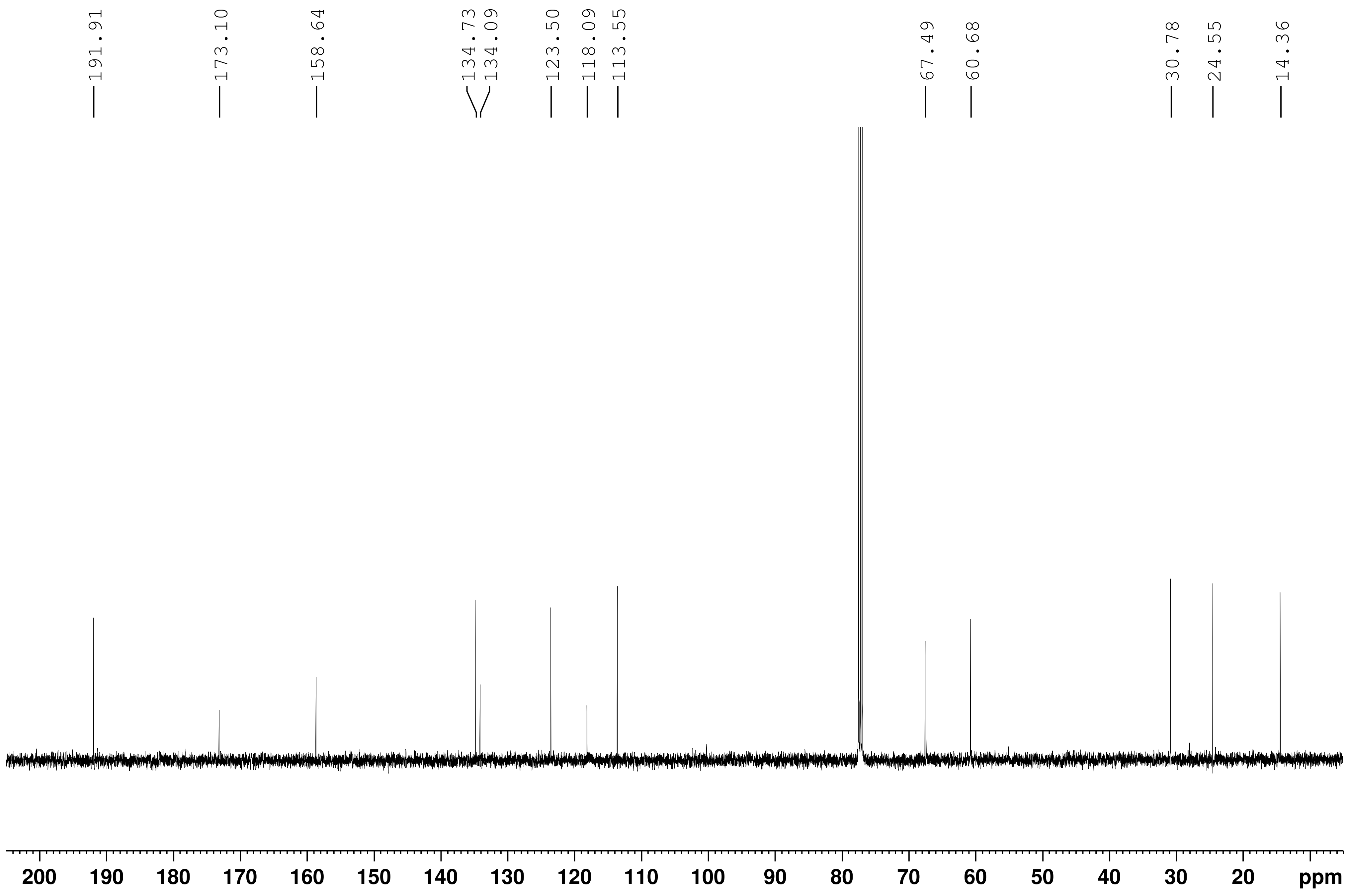

Figure S26. ^{13}C NMR spectrum of **1a** in $CDCl_3$.

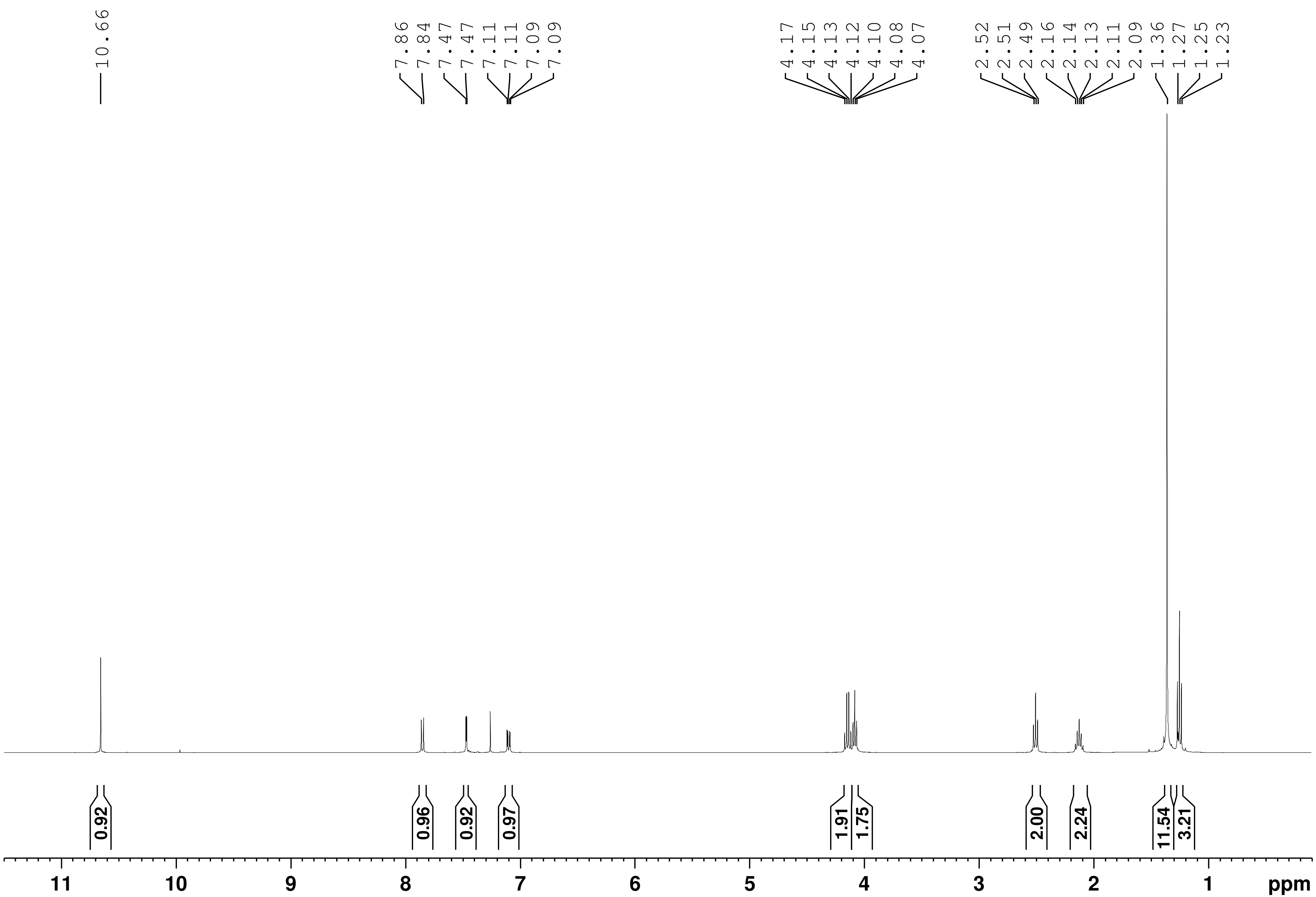

Figure S27. ^{1}H NMR spectrum of **2a** in $CDCl_3$.

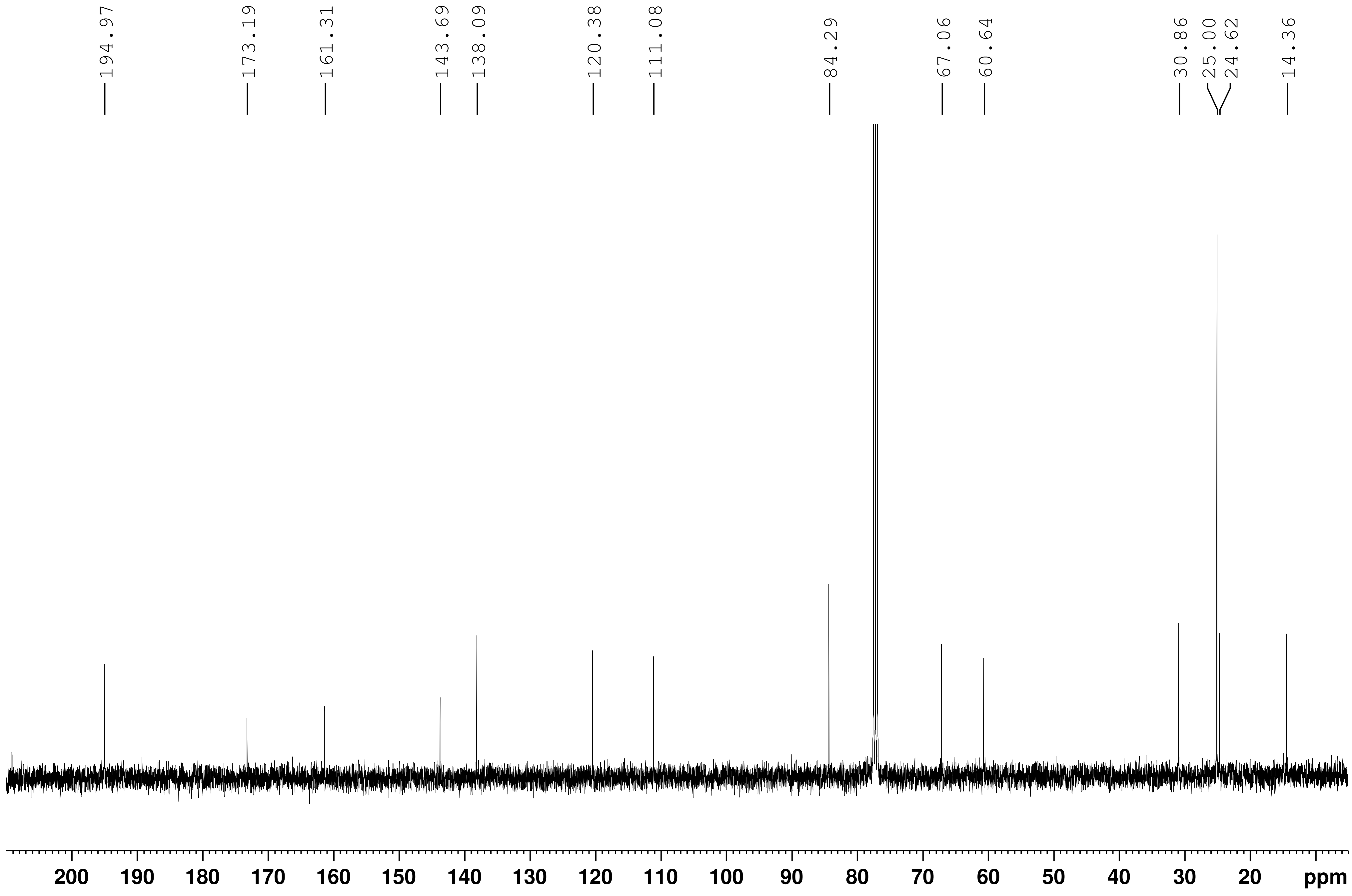

Figure S28. ^{13}C NMR spectrum of **2a** in $CDCl_3$.

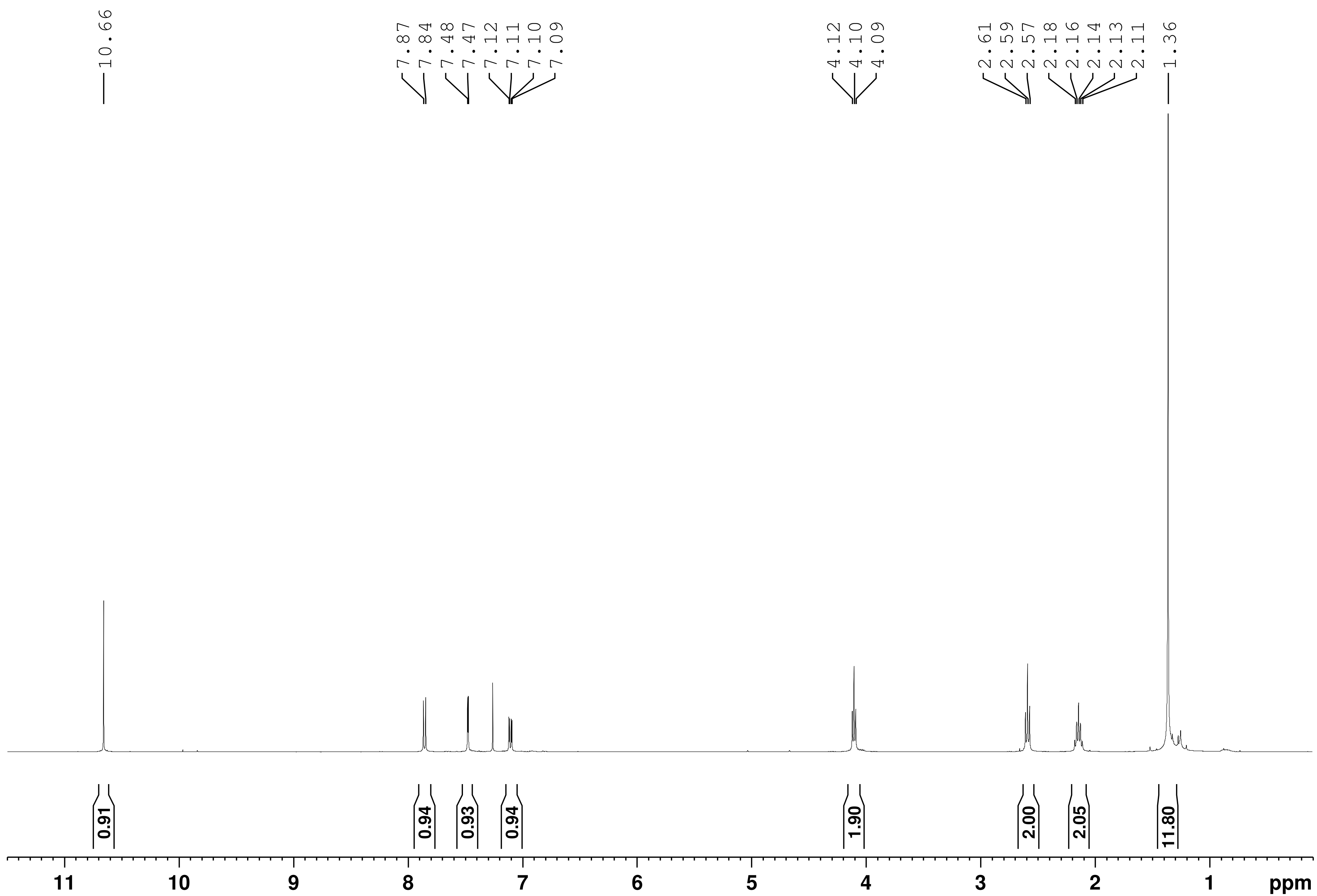

Figure S29. ^{1}H NMR spectrum of **3a** in $CDCl_3$.

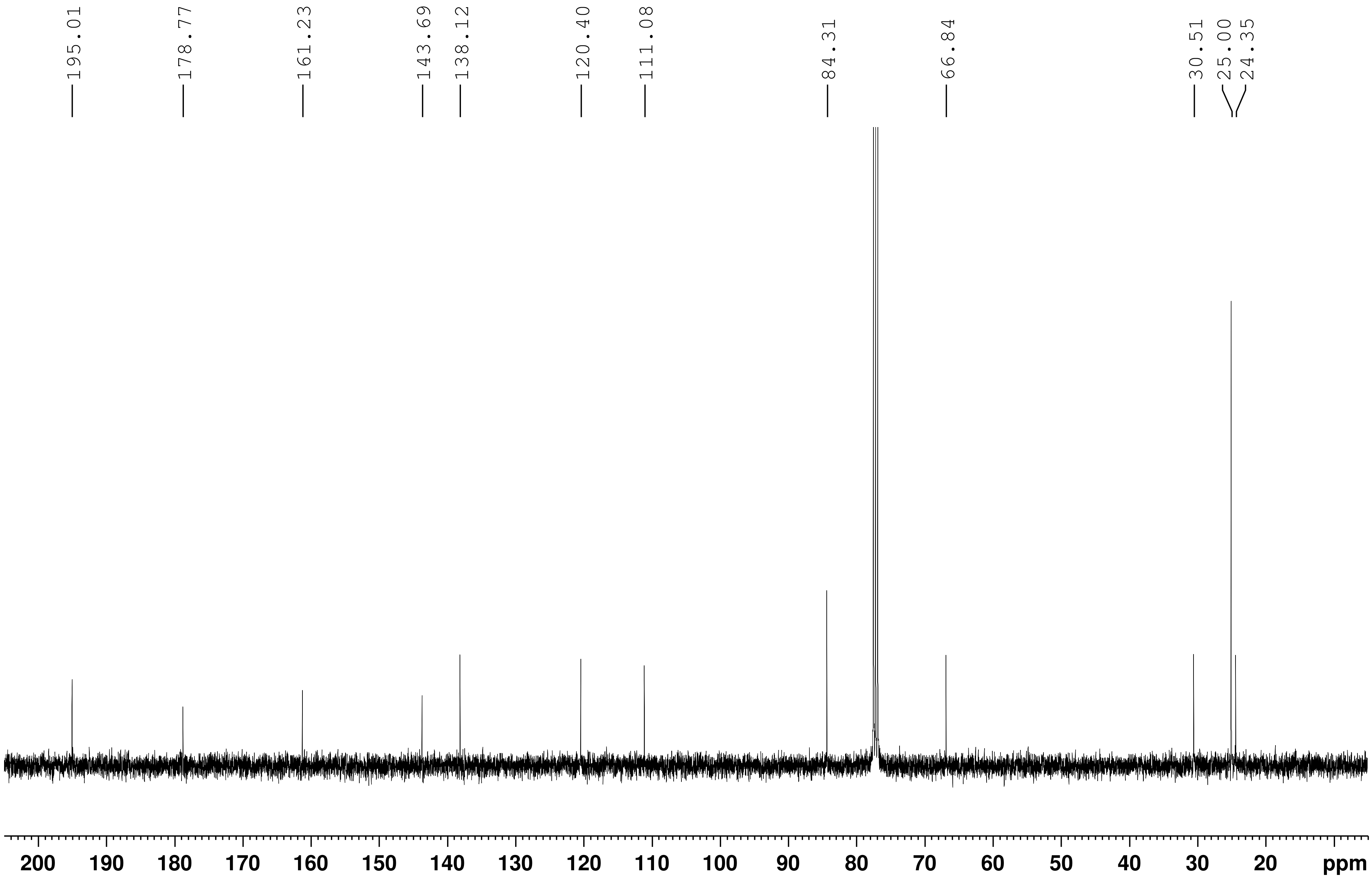

Figure S30. ^{13}C NMR spectrum of **3a** in $CDCl_3$.

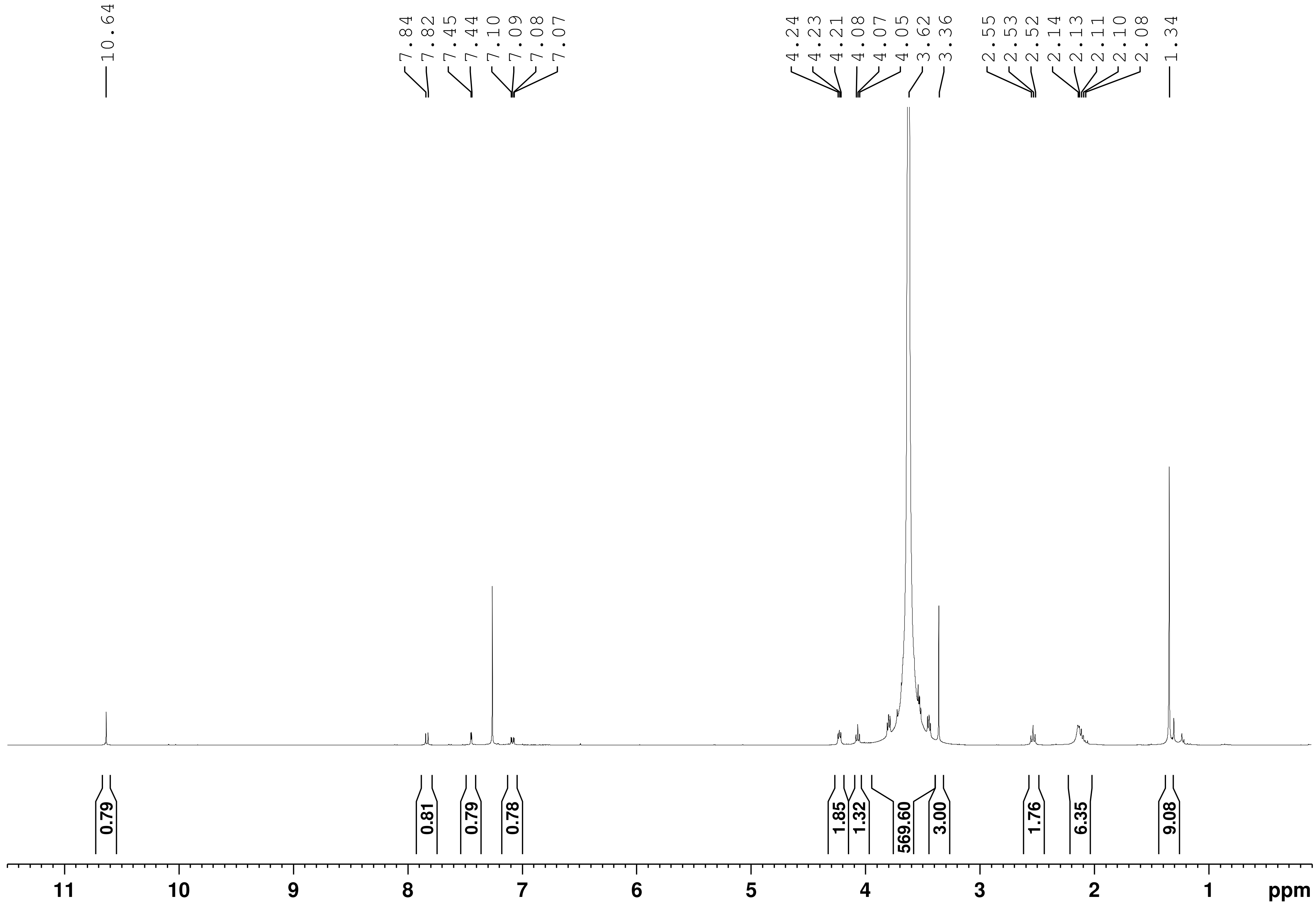

Figure S31. ^{1}H NMR spectrum of **4a** in $CDCl_3$.

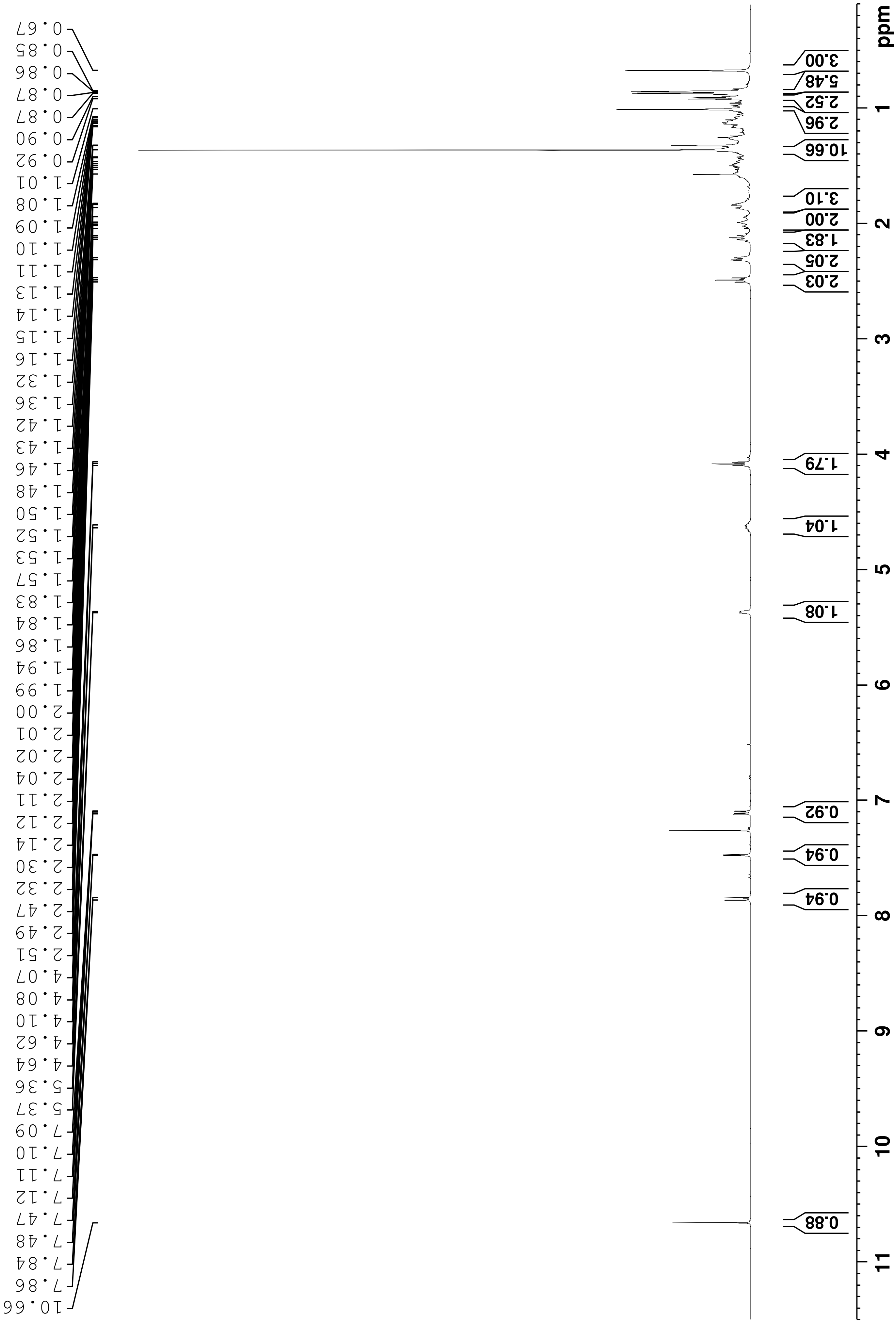

Figure S32. ^{1}H NMR spectrum of **5a** in $CDCl_3$.
